# Nucleophilic reactivity of the gold atom in a diarylborylgold(i) complex toward polar multiple bonds†

**DOI:** 10.1039/d0sc05478j

**Published:** 2020-11-18

**Authors:** Akane Suzuki, Xueying Guo, Zhenyang Lin, Makoto Yamashita

**Affiliations:** Department of Molecular and Macromolecular Chemistry, Graduate School of Engineering, Nagoya University Furo-cho, Chikusa-ku Nagoya 464-8603 Aichi Japan makoto@oec.chembio.nagoya-u.ac.jp; Department of Chemistry, The Hong Kong University of Science and Technology Clear Water Bay Kowloon Hong Kong chzlin@ust.hk

## Abstract

A di(*o*-tolyl)borylgold complex was synthesized *via* the metathesis reaction of a gold alkoxide with tetra(*o*-tolyl)diborane(4). The resulting diarylborylgold complex exhibited a Lewis acidic boron center and a characteristic visible absorption that arises from its HOMO–LUMO excitation, which is narrower than that of a previously reported dioxyborylgold complex. The diarylborylgold complex reacted with isocyanide in a stepwise fashion to afford single- and double-insertion products and a C–C coupled product. Reactions of this diarylborylgold complex with C=O/N double bond species furnished addition products under concomitant formation of Au–C and B–O/N bonds, which suggests nucleophilic reactivity of the gold metal center. DFT calculations provided details of the underlying reaction mechanism, which involves an initial coordination of the C=O/N bond to the boron vacant p-orbital of the diarylboryl ligand followed by a migration of the gold atom from the tetracoordinate sp^3^-hybridized boron center, which is analogous to the reactivity of the conventional sp^3^-hybridized borate species. The DFT calculations also suggested a stepwise mechanism for the reaction of this diarylborylgold complex with isocyanide, which afforded three different reaction products depending on the applied reaction conditions.

## Abstract 2

A di(*o*-tolyl)borylgold complex added to C=O/N double bond to form Au–C and B–O/N bonds. DFT calculations revealed a two-step mechanism consisting of the coordination of O/N atom to B atom followed by nucleophilic migration of Au atom.

## Graphical abstract

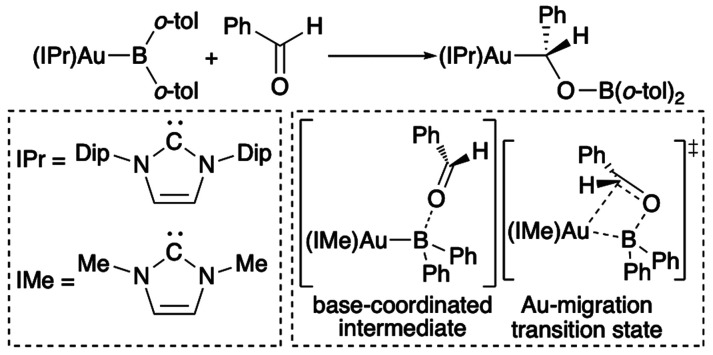

## Introduction

### Property of transition-metal–boryl complexes: (a) synthetic equivalent to boron nucleophile

**Scheme 1:**
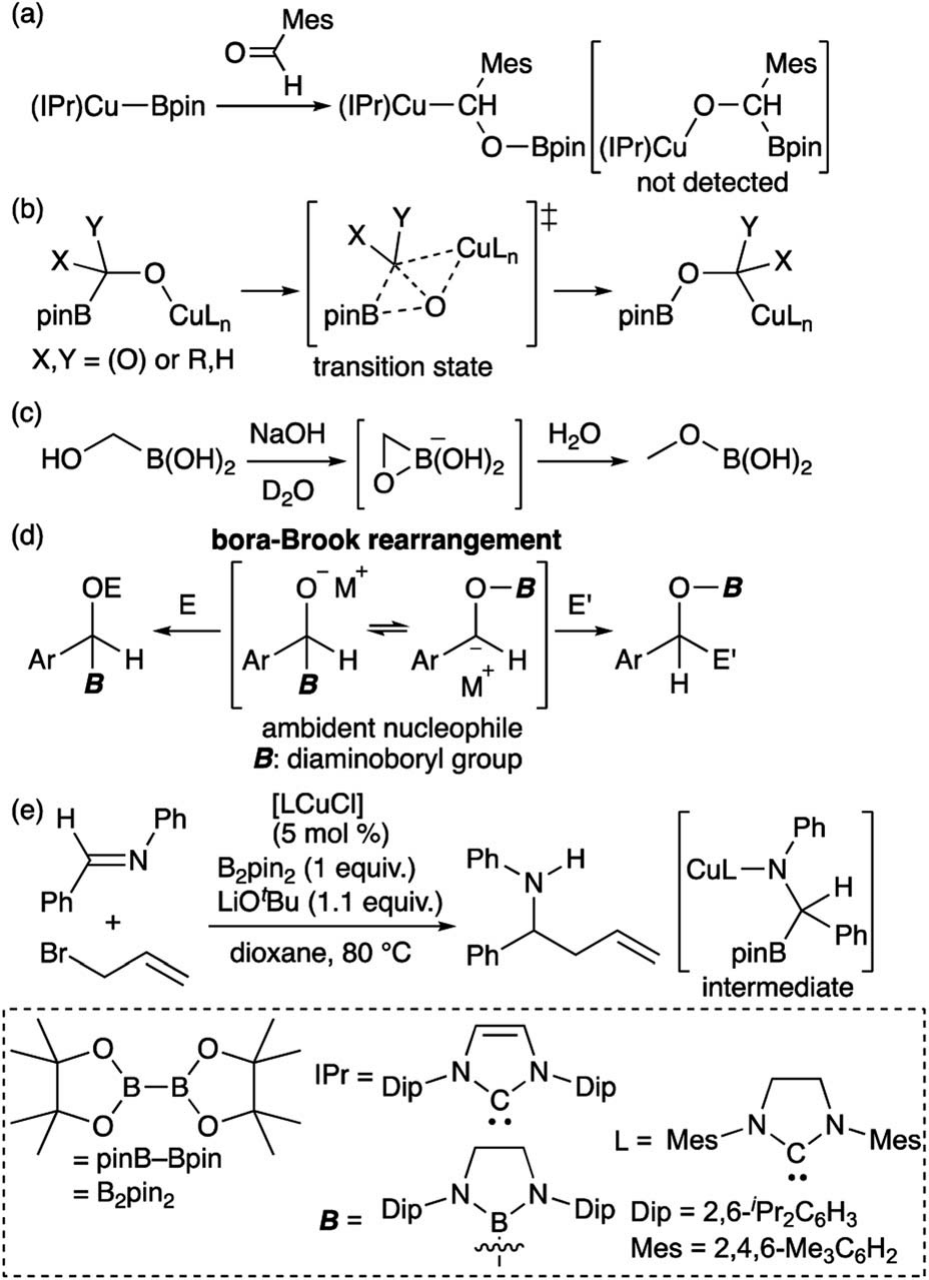
(a) Reaction of isolated borylcopper complex with aldehyde; (b) DFT-calculated mechanism of boryl migration from the carbon to the oxygen atom; (c) boryl migration from the carbon to the oxygen atom of an alkoxide; (d) bora-Brook rearrangement; and (e) catalytic addition of allyl bromide to an imine.

Transition metal (TM)–boryl complexes with a three-coordinate boron atom as an X-type ligand on the TM center have been identified as key intermediates in TM-catalyzed borylation reactions of organic molecules.^1^ In most hitherto reported TM–boryl complexes, oxygen and nitrogen substituents have been used to enhance the stability of the complex through pπ–pπ interactions between the heteroatoms and the boron center. Due to the polarized character of the TM^δ+^–B^δ−^ bond, TM–boryl complexes have been widely applied as “boron nucleophiles”^2^ since the discovery of this reactivity of *in situ*-generated borylcopper reagents.^3^ It should be noted here that the reaction of the isolated (IPr)Cu–Bpin complex with aldehyde affords the boroxybenzylcopper complex (Scheme 1a), rather than the borylbenzyloxycopper complex.^4^ Considering the nucleophilic character of boryl ligand in the TM–boryl complex,^3^ this reactivity would be strange because “nucleophilic” boryl group attached to the oxygen atom. Subsequent theoretical calculations have indicated that the reaction proceeds in a two-step fashion (Scheme 1b), *i.e.*, *via* a nucleophilic attack of the Cu-bonded “anionic” Bpin moiety on the carbonyl carbon of the aldehyde, followed by the migration of the Bpin group from the carbon atom to the oxygen atom through a transition state that involves a three-membered BCO ring.^5^ Thus, ambiphilic character of the boryl ligand arising from the TM–B bonding electrons and the vacant p-orbital of the boron atom would lead to this unusual two-step reaction. A similar migration of the boryl group has been reported for the reaction of hydroxymethylborane with NaOH to form the corresponding methoxyborane product (Scheme 1c).^6^ We have reported a detailed kinetic study on a similar migration of a boryl group from the carbon to the oxygen atom *via* a “bora-Brook rearrangement” (Scheme 1d).^7^ In the Cu-catalyzed allylation of imine in the presence of B_2_pin_2_ and allyl bromide would also involve the nucleophilic attack of Bpin to form borylamido-Cu intermediate followed by a boryl migration (Scheme 1e).^8^

### Property of transition-metal–boryl complexes: (b) Lewis acidity at the boron atom

**Scheme 2:**
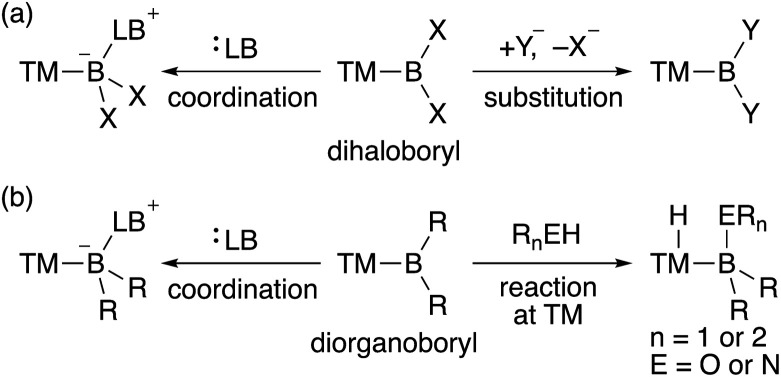
Reactivity of (a) TM–dihaloboryl and (b) –diorganoboryl complexes (TM: transition metal; X: halogen; Y: nucleophile; LB: Lewis base; R: alkyl or aryl).

In contrast to the dioxy- and diamino-boryl–TM complexes, one can expect that boryl–TM complexes with no donor substituents exhibit strong Lewis acidity on the boron center. Although several theoretical studies on the properties of terminal TM–BH_2_ (dihydroboryl) complexes have been published,^9^ reports on their synthesis are not available, probably due to the almost complete lack of steric hindrance. Additionally, TM–dihaloboryl^10^ and –diorganoboryl^10*a*–*d*,*g*,*ad*,11^ complexes would be classified as a strongly Lewis acidic TM–boryl complex (Scheme 2). The boron center of these electrophilic TM–boryl complexes can accept a lone pair of electrons to form the corresponding base-stabilized boryl complexes (Scheme 2a, left; Scheme 2b, left).^10*k*,*l*,*o*,*p*,*ad*^ Dihaloboryl complexes could also undergo substitution at the boron center through elimination of halides (Scheme 2a, right).^10*o*,*t*,*v*,*y*,*ab*,12^ In addition to the monodentate diorganoboryl complexes, diorganoboryl-based pincer complexes have been reported to exhibit characteristic reactivity in organometallic reactions and catalysis.^13^ Notably, a diarylboryl-based PBP-pincer Ir complex reacted with O–H and N–H bonds to form iridium hydride complexes *via* protonation on the Ir center (Scheme 2b, right), indicating the strong Brønsted basicity of the Ir center due to the strongly σ-donating diarylboryl ligand. So far, a DFT study has been reported to demonstrate the electrophilicity of a Bpin ligand in Pt-catalyzed diboration of electron-poor alkenes.^14^ However, reports on the nucleophilic reactivity of the TM center in TM–boryl complexes remain extremely rare and/or elusive.

### Property of transition-metal alumanyl complexes: Lewis acidity at the aluminium atom and nucleophilicity at the transition metal center

**Scheme 3:**
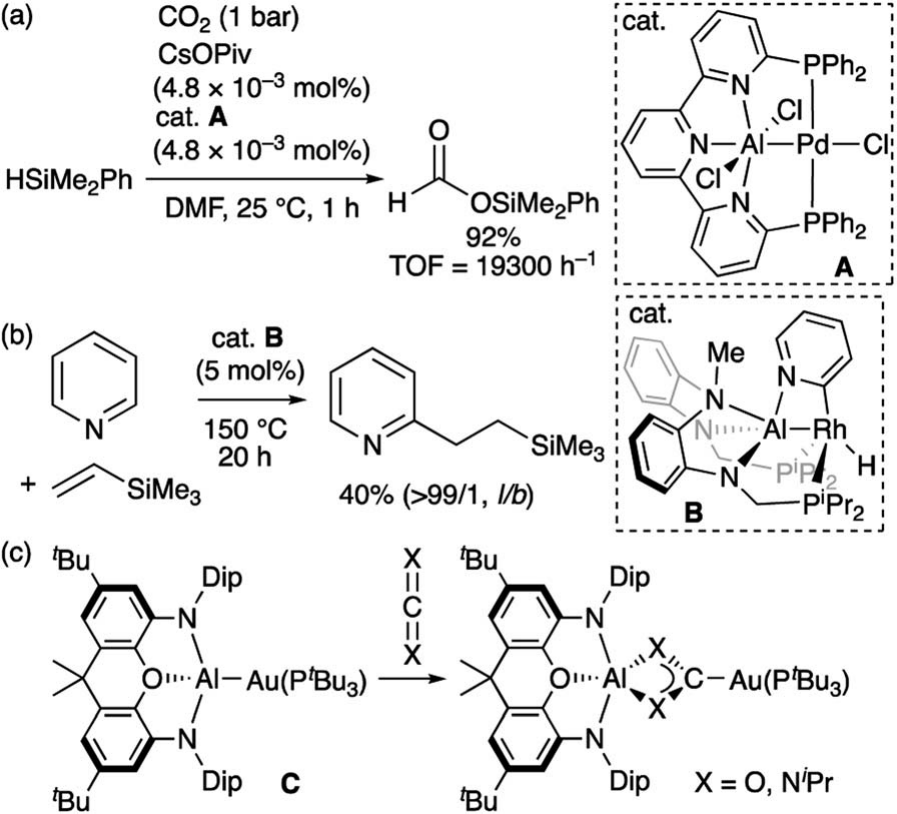
(a)–(c) Characteristic reactions of TM-alumanyl complexes in which a Lewis base is coordinated to the Al atom.

Compared to the chemistry of TM–B complexes, that of TM–Al complexes is rather limited. Although there are only five examples of isolated TM-alumanyl complexes^15^ with a three-coordinated Al atom, a strategy involving stabilization with a Lewis base allows observing base-stabilized TM-alumanyl complexes.^16^ Two characteristic reactions have been reported using isolated complexes of this type. One is the catalytic transformation of CO_2_ or pyridine into silylformate or alkylpyridines using two different PAlP-pincer complexes (A and B; Scheme 3a and b).^16*f*,*h*^ The other is based on the “nucleophilic” reactivity of the gold center in Au–Al complex C toward CO_2_ and carbodiimide to form Au–C bond (Scheme 3c).^16*j*^ Theoretical calculations on A have indicated that the Al atom is more positively charged than the Pd atom.^16*f*^ Similarly, DFT calculations suggested that B exhibits the reverse polarization of the Rh^δ−^–Al^δ+^ bond.^17^ Due to the higher electronegativity of the gold atom (2.04) compared to that of the aluminium atom (1.61),^18^ the similarly polarized Au^δ−^–Al^δ+^ bond in C is proposed by calculations. These X-type anionic Al ligands thus afford an electron-rich Al-bonded TM center. This is in stark contrast to the chemistry of TM–boryl complexes as noted above.

Herein, we report the synthesis and electronic properties of a diarylborylgold complex. The reaction of the diarylborylgold complex with one or two equivalents of isocyanide furnished, depending on the reaction conditions, one of three products with a stepwise reaction mechanism supported by DFT calculations. In the reaction of this diarylborylgold complex with C=X (X = O or N) bonds, the formation of an Au–C bond was observed in all cases. A DFT-based mechanistic study revealed that the products were formed *via* the initial coordination of the X center of the C=X bond to the diarylboryl ligand followed by a migration of the gold center.

## Results and discussion

### Synthesis and properties of diarylborylgold complex 2

**Scheme 4:**
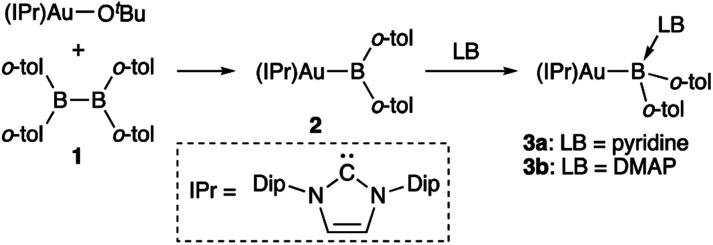
Synthesis of diarylborylgold complex 2 and its complexation with a Lewis base (LB: pyridine or DMAP) at the B atom.

**Fig. 1:**
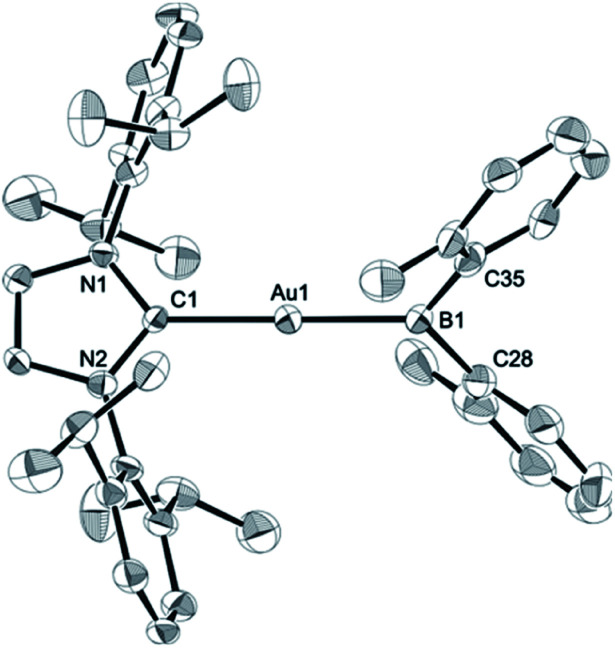
Molecular structures of 2 with thermal ellipsoids at 50% probability; disordered *o*-tolyl groups, one of two independent molecules, and hydrogen atoms omitted for clarity.

**Fig. 2:**
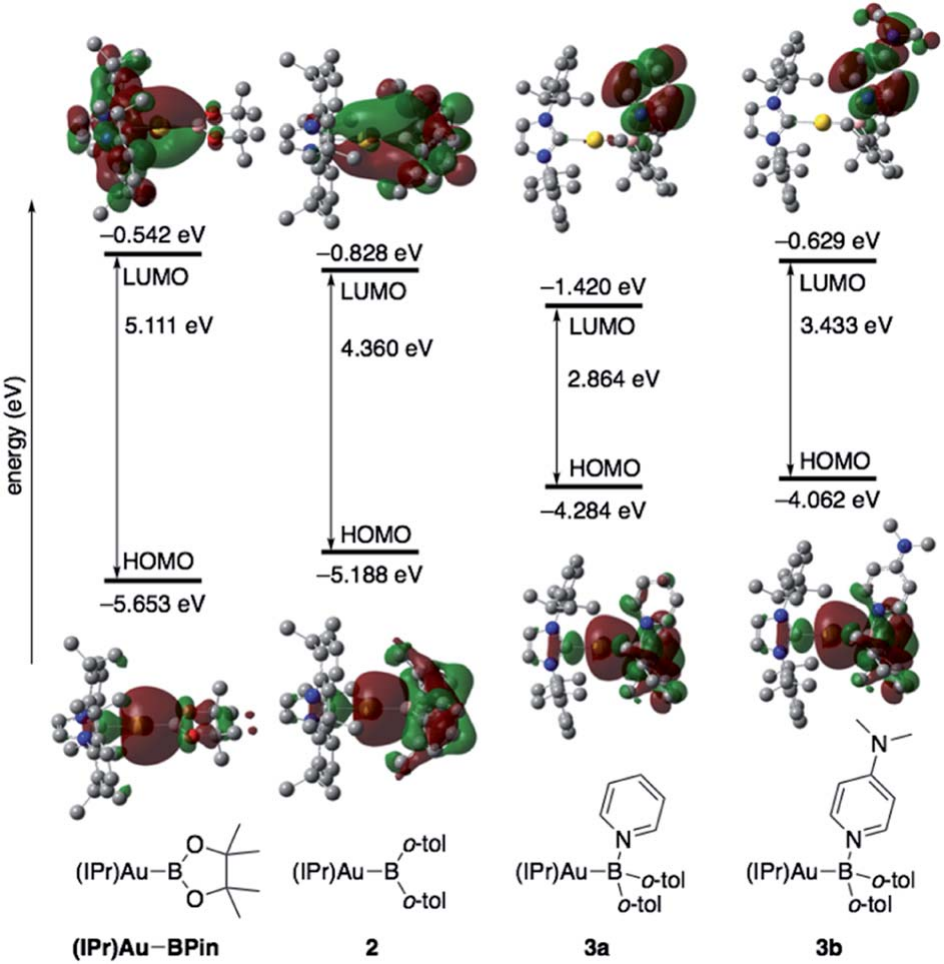
Comparison of the frontier molecular orbitals of (IPr)Au–Bpin,^19^2, 3a, and 3b together with their energy levels.

**Fig. 3:**
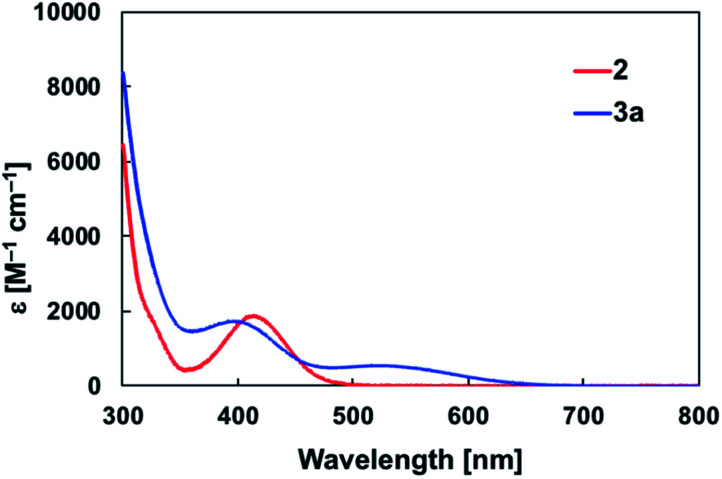
UV-Vis spectra of 2 and 3a in toluene solution at room temperature.

The diarylborylgold(i) complex 2 was obtained in the form of orange crystals in 68% yield from the reaction of 1 with (IPr)Au–O^*t*^Bu under concomitant elimination of (*o*-tol)_2_BO^*t*^Bu *via* a metathesis reaction (Scheme 4). The ^1^H and ^13^C NMR spectra of 2 reflect its *C*_2v_ symmetrical structure. The ^11^B NMR signal of 2 (*δ*_B_ 109 ppm) is downfield shifted compared to that of 1 (*δ*_B_ 89 ppm),^19^ which supports the formation of a B–Au bond. A single-crystal X-ray diffraction analysis revealed a monomeric structure without intermolecular aurophilic interactions, probably due to the steric hindrance. The linear C–Au–B arrangement (177.7(3)°/179.4(3)°) and the B–Au bond lengths (2.068(6)/2.068(9) Å) in 2 (Fig. 1) are similar to those observed in previously reported dioxyborylgold complexes^19^ and diaminoborylgold complexes.^20^ In contrast, the Au–C bond (2.104(7)/2.095(7) Å) in 2 is longer than that of (IPr)Au–Bpin (2.084(4) Å), which contains the same N-heterocyclic carbene (NHC) ligand.^19^ The longer Au–C bond reflects the stronger *trans* influence of the B(*o*-tol)_2_ ligand compared to that of the Bpin ligand due to the lower electronegativity of the C atoms of the *o*-tolyl substituents compared to that of the oxygen atoms in the Bpin ligand. Based on DFT calculations at the PBE0/SDD(Au)/6-311G(d)(all others) level of theory, the frontier orbitals of 2 reflect the characteristics of the diarylboryl ligand (Fig. 2). The HOMO of 2 exhibits significant contributions from the Au–B σ-bond and the π-orbitals of the *o*-tolyl groups, while the LUMO consists of the vacant p- and d-orbitals on the B and Au atoms, the π*-orbitals of the *o*-tolyl groups, and the vacant p-orbital on the carbon atom in the IPr ligand. Compared to the frontier orbitals of (IPr)Au–Bpin (ref. 19) which were independently calculated at the same level of theory in this study, the HOMO and LUMO of 2 are higher and lower respectively, which indicates stronger σ-donor and π-acceptor properties for the diarylboryl ligand in 2 relative to those of the Bpin ligand in (IPr)Au–Bpin. As a result of the narrower HOMO–LUMO gap of 2 compared to those of colorless dioxyboryl- and diaminoboryl-gold complexes, the UV-Vis absorption spectrum of 2 shows an absorption maximum at 413 nm (*ε* 1860) as illustrated in Fig. 3. The visible absorption of 2 was attributed to the transition from the occupied B–Au bonding orbital to the vacant p-orbital of the B atom, similar to that of a recently reported dialkylalumanyl-yttrium complex.^15*e*^

### Complexation of diarylborylgold complex 2 with pyridine and DMAP

The addition of pyridine or 4-dimethylaminopyridine (DMAP) to 2 led to the formation of 3a and 3b (ref. 21) *via* the coordination of pyridine or DMAP to the Lewis-acidic boron center (Scheme 4). Upon coordination, the ^11^B NMR signals of 3a (*δ*_B_ 15) and 3b (*δ*_B_ 11) shift to higher field compared to that of 2, supporting the sp^3^-hybridization of the B atom in 3a and 3b. It should be noted here that the previously reported complex (IPr)Au–Bpin (ref. 19) does not react with DMAP, which was independently confirmed in this study. The crystallographic analysis of 3a and 3b confirmed the sp^3^-hybridization of the B atom in 3a and 3b (Fig. S43 and S44†). The B–Au bond (3a: 2.160(4) Å; 3b: 2.158(8) Å) and the B–C bond (3a: 1.621(5)/1.638(4) Å; 3b: 1.630(9)/1.634(9) Å) are longer than those of 2, reflecting the larger size of the sp^3^-hybridized B center in 3a and 3b relative to that on 2. The B–N bonds (3a: 1.679(4) Å; 3b: 1.648(6) Å) are similar to or longer than those of the B–N(pyridine) bonds in previously reported TM–boryl complexes (1.596(2)–1.639(10) Å).^10*k*,*o*,*y*,13*a*,22^ The HOMO of 3a, which has the Au–B σ-bond character, lies energetically higher than that of 2, which reflects the coordination of pyridine to the B atom (Fig. 2). In contrast, the LUMO of 3a consists almost purely of the π*-orbital of the coordinated pyridine ring, leading to a narrower HOMO–LUMO gap. This elevated HOMO level and lowered LUMO level are directly related to the reactivity of 2 toward C=O/C=N bonds (*vide infra*). The slightly stronger donor ability of DMAP raises both the energy levels of the HOMO and LUMO of 3b compared to those of 3a; this effect was even stronger for the LUMO with π*-character. The UV-Vis absorption spectrum of 3a showed two absorption maxima at 398 nm (*ε* 1740) and 523 nm (*ε* 550) (Fig. 3), whereby the latter was attributed to a charge-transfer-type absorption resulting from the spatial separation of the HOMO and LUMO in 3a.

**Scheme 5:**
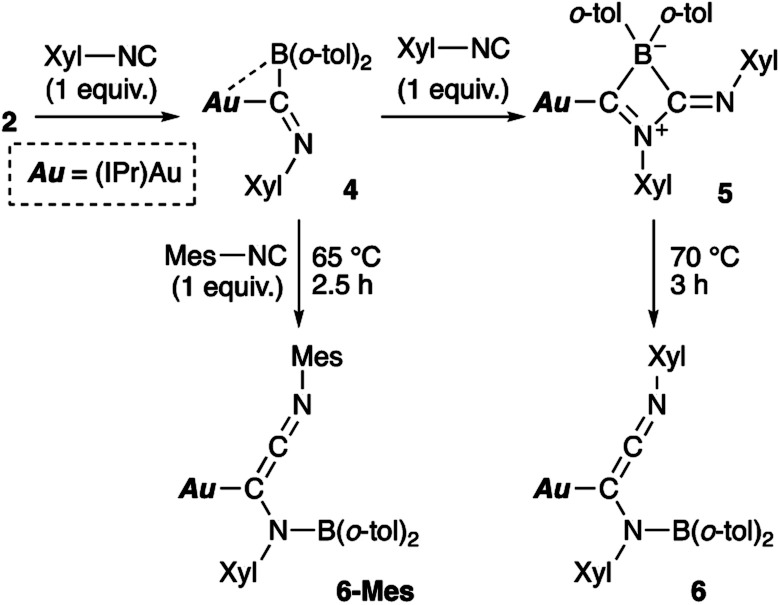
Reaction of 2 with Xyl-substituted isocyanide.

### Reaction of diarylborylgold complex 2 with isocyanide

Then, we examined the reaction of diarylborylgold(i) complex 2 with Lewis-basic isocyanides (Scheme 5). Treatment of 2 with one equivalent of 2-xylyl-substituted isocyanide (Xyl-NC) afforded insertion product 4. Similar insertions of isocyanides into TM–B bonds have been reported for Cu,^23^ Fe,^24^ and Au^25^ complexes. The reaction of 4 with an additional equivalent of Xyl-NC afforded 5, which contains a four-membered ring, *via* insertion of the second equivalent of isocyanide. This behavior is consistent with the previously reported reaction of a Mn–BCl_2_ complex with *tert*-butylisocyanide.^24*b*^ The ^11^B NMR signal of 5 (*δ*_B_ 4) was shifted upfield, which supports the formation of a tetrahedral borate. The relative sharpness of the ^1^H NMR signals corresponding to the methyl groups indicates flexibility for the *o*-tol and Xyl groups. Gentle heating of 5 induced a skeletal rearrangement to form azaallenylgold complex 6 through C=C bond formation. It is noteworthy here that aminoborylene–Cr and Fe complexes and Lewis-acidic diboranes(4) undergo similar C–C-bond-forming reactions to generate isocyanide dimers.^24*a*,26^ The ^11^B NMR signal of 6 (*δ*_B_ 43) was downfield shifted relative to that of 5, reflecting the regeneration of the sp^2^-hybridized B center. Similar to the case of 5, the ^1^H NMR spectrum of 6 exhibited sharp signals for methyl groups, indicating a flexible molecular structure. Treatment of 5 with Mes-substituted isocyanide furnished 6-Mes with a mesityl group on the terminal N atom of the azaallene moiety (Scheme 5), which was confirmed by a crystallographic analysis. This result indicates that the N≡C moiety in the second isocyanide molecule was converted into the terminal N=C bond of the azaallene functionality.

**Fig. 4:**
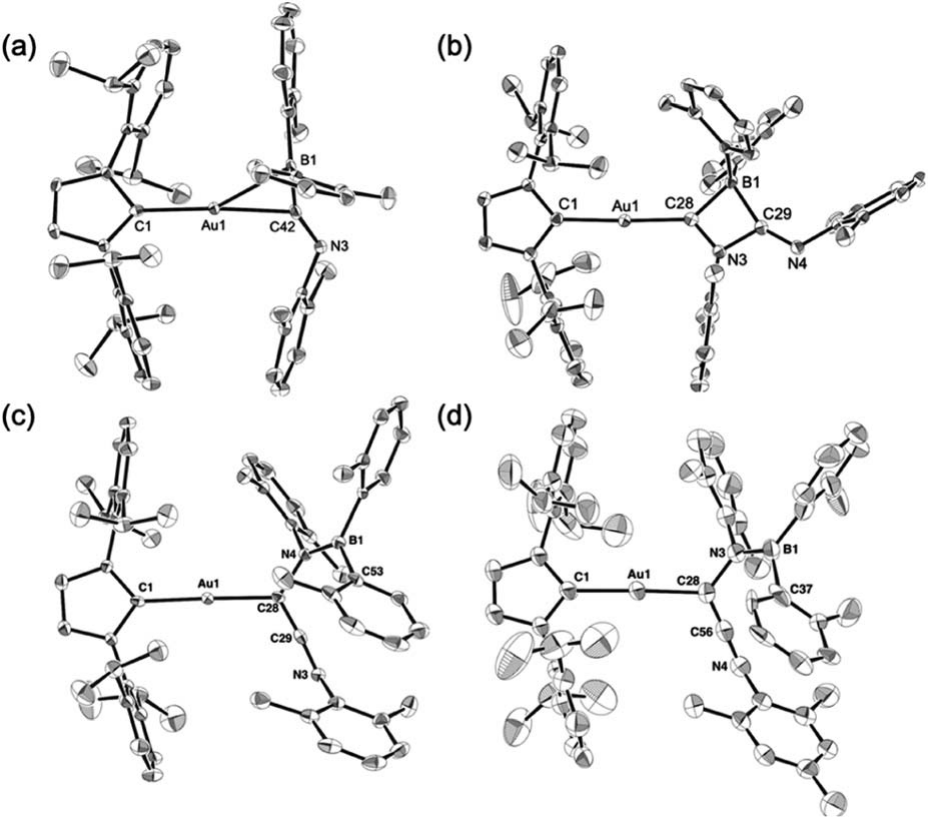
Molecular structures of (a) 4, (b) 5, (c) 6, and (d) 6-Mes with thermal ellipsoids at 50% probability; hydrogen atoms omitted for clarity.

Compounds 4–6 and 6-Mes were structurally characterized using single-crystal X-ray diffraction analyses (Fig. 4). The crystal structure of 4, in which the unit cell contains two independent molecules, exhibits interesting close contacts between the Au and B centers (2.428(3)/2.512(4) Å) leading to a small Au–C–B angles (81.32(19)°/85.4(2)°). Considering the planar structure of the B atoms (∑∠B = 359.7°/360.0°), the interactions between the Au and B atoms can be expected to be relatively weak, as quantum theory of atoms in molecules (QTAIM) analysis of 4 did not indicate any bond paths between the Au and B atoms (for details, see Fig. S43†). In other words, the sterically undemanding diarylboryl substituent in 4 reduces the steric repulsion between the Xyl group and the Dip substituent of the IPr ligand on the Au atom. The Xyl substituent on the N atom in the imidoyl moiety (C=N: 1.262(4)/1.257(4) Å) leans toward the Au atom, probably due to the crystal-packing forces.

The four-membered ring in 5 is almost planar (∑∠_internal_: 359.94°) and slightly distorted due to the large covalent radius of the sp^3^-hybridized B atom (C–B: 1.656(4)/1.696(4) Å) and the short endocyclic C=N double bond (1.322(3) Å). The exocyclic C=N double bond (1.265(3) Å) is comparable to that in 4. The two Au–C bonds differ slightly in length (Au–C_IPr_: 2.034(2) Å; Au–C_alkenyl_: 2.003(2) Å), indicating the covalent character for the Au–C_alkenyl_ bond.

In the structure of the rearrangement product 6, the azaallenyl moiety exhibits short C=C (1.310(3) Å) and C=N (1.233(3) Å) double bonds. The substituents of the trigonal planar B and N atoms in the newly formed B–N bond are almost coplanar (∠C–N–B–C: 10.2(3)°), reflecting the double bond character between the B and N atoms (B–N: 1.404(3) Å). Similar to those in 5, the two Au–C bonds in 6 slightly differ in length. All the structural parameters of 6-Mes are almost identical to those of 6.

### Reaction of diarylborylgold complex 2 with compounds that contain C=O or C=N double bonds

**Scheme 6:**
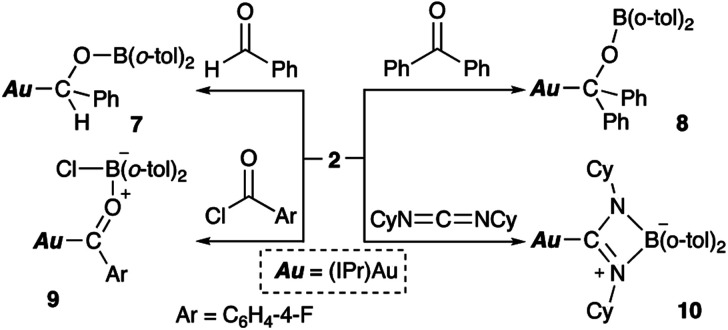
Reaction of 2 with C=X multiple bonds (X = N, O).

Complex 2 also reacts with compounds that contain C=O or C=N double bonds (Scheme 6). Reaction with benzaldehyde affords the simple adduct 7*via* the formation of Au–C and O–B bonds. The observation of a characteristic benzyl proton signal with a relatively downfield chemical shift (*δ*_H_ 5.72 ppm) and signals of the six methyl groups of the *o*-tolyl and Dip groups in the ^1^H NMR spectrum of 7 supports the existence of a chiral center at the benzylic position in 7. Benzophenone also reacts with 2, furnishing a similar adduct (8), albeit with a more symmetrical structure, which is supported by ^1^H NMR spectroscopy. The reaction of *p*-fluorobenzoyl chloride with 2 results in the formation of *p*-fluorobenzoylgold–chloroborane complex 9*via* the formation of Au–C, O–B, and B–Cl bonds. The reaction of 2 with cyclohexyl-substituted carbodiimide produced Au-substituted (amidinato)boron compound 10. The ^11^B NMR spectra of alkoxy-substituted 7 and 8 exhibit broad signals that correspond to their sp^2^-hybridized B atom and that are downfield shifted (7: *δ*_B_ 47 ppm; 8: *δ*_B_ 45 ppm) compared to those of 9 and 10 (9: *δ*_B_ 10 ppm; 10: *δ*_B_ 7 ppm), which contain an sp^3^-hybridized B atom.

**Fig. 5:**
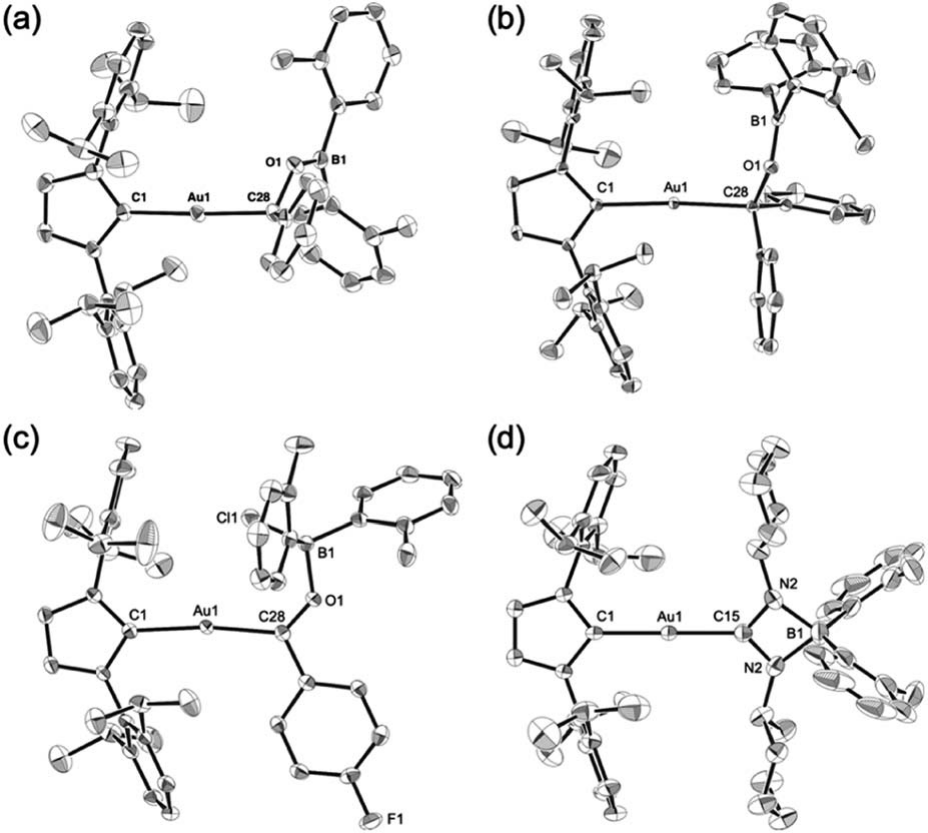
Molecular structures of (a) 7, (b) 8, (c) 9, and (d) 10 with thermal ellipsoids at 50% probability; hydrogen atoms are omitted for clarity.

Complexes 7–10 were structurally characterized by single-crystal X-ray diffraction analyses, and were all found to contain Au–C bonds (Fig. 5). In the structure of 7, the length of the Au–C_carbene_ bond (2.031(3) Å) is similar to those in 5 and 6, while the length of its Au–C_alkyl_ bond (2.075(3) Å) is longer than those in 5 and 6 due to the sp^3^-hybridization of the C atom. The substituents on the newly formed B–O bond adopt a coplanar alignment (∠C–O–B–C: −1.5(5)°), indicating double bond character between the B and O atoms (1.346(4) Å). The structural features of the benzophenone adduct 8 are very similar to those of 7. In the crystal structure of 9, the Au–C_acyl_ bond (2.032(2) Å) is shorter than those in 7 and 8, which is probably due to the sp^2^-hybridization of the acyl C atoms in 7 and 8. As the B atom in 9 is sp^3^-hybridized due to the coordination of the chloride, its B–O bond, which is longer (1.552(3) Å) than those in 7 and 8, can be considered to be a single bond. The four-membered borate structure with a metal substituent and relatively short C–N (1.339(4) Å; partial double bond) and long B–N (1.603(6) Å; single bond) bonds in 10 is similar to that of the previously reported CpFe(CO)_2_-substituted amidinate–borate.^27^

### Mechanistic studies based on DFT calculations

#### Reaction of 2 with compounds that contain C=O or C=N double bonds

**Scheme 7:**
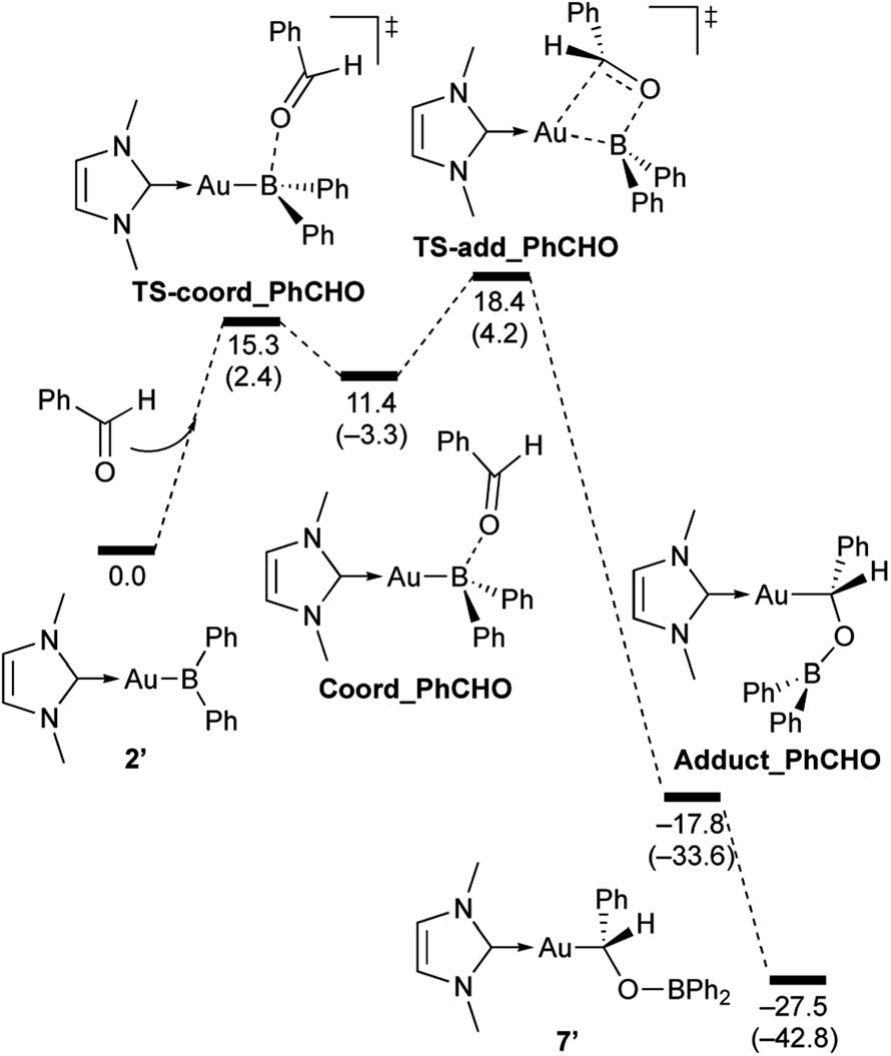
Calculated energy profile for the reaction of model compound 2′ with PhCHO to form 7′. Relative free energies and electronic energies (in parentheses) are given in kcal mol^−1^.

**Scheme 8:**
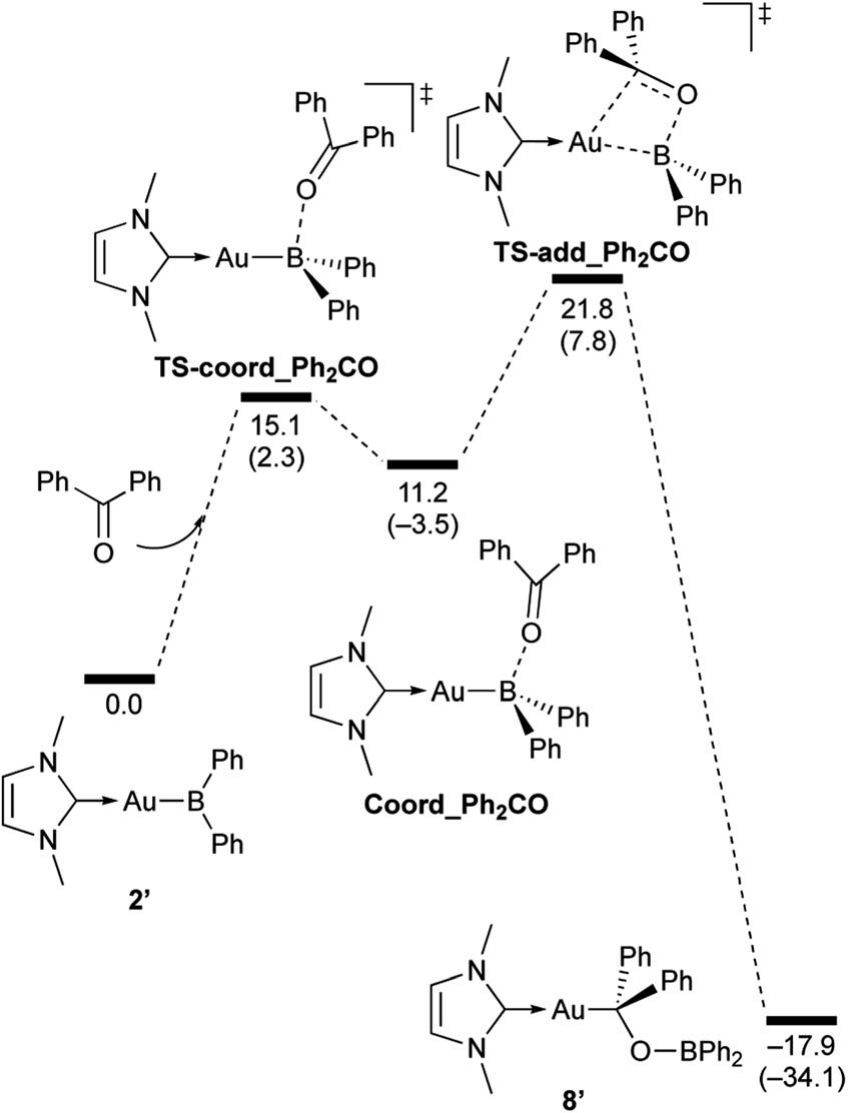
Calculated energy profile for the reaction of model compound 2′ with benzophenone to form 8′. Relative free energies and electronic energies (in parentheses) are given in kcal mol^−1^.

**Scheme 9:**
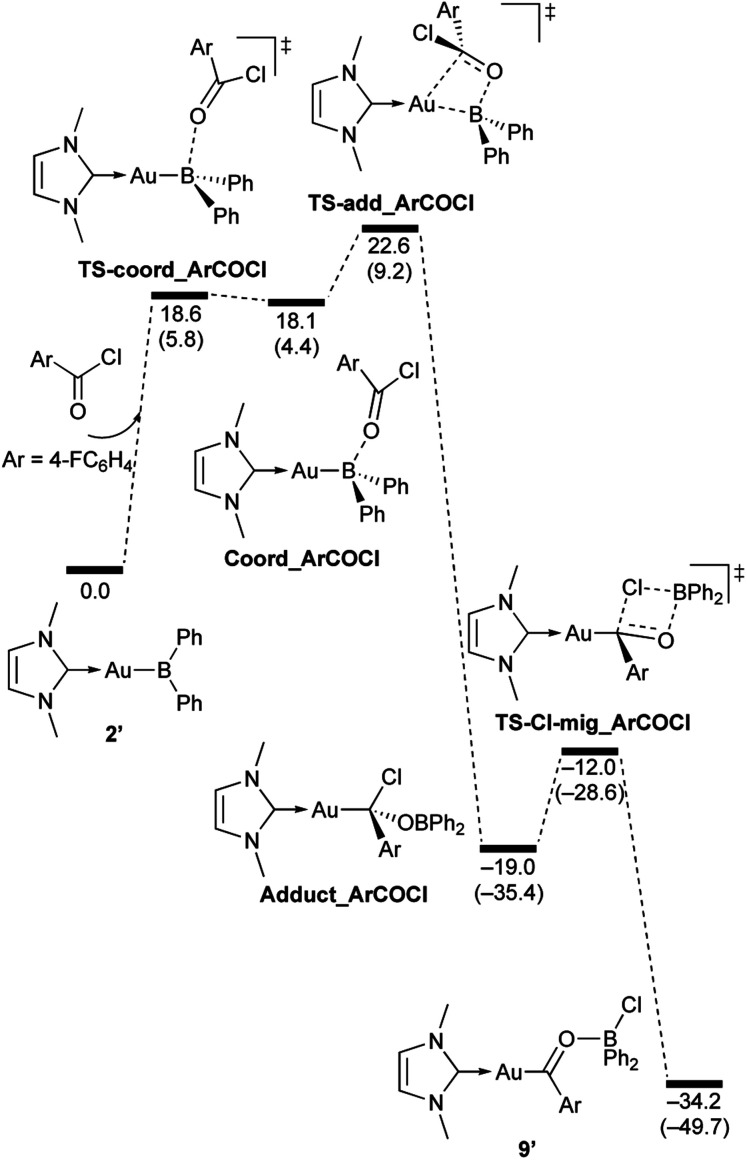
Calculated energy profile for the reaction of model compound 2′ with *p*-FC_6_H_4_COCl to form 9′. Relative free energies and electronic energies (in parentheses) are given in kcal mol^−1^.

**Scheme 10:**
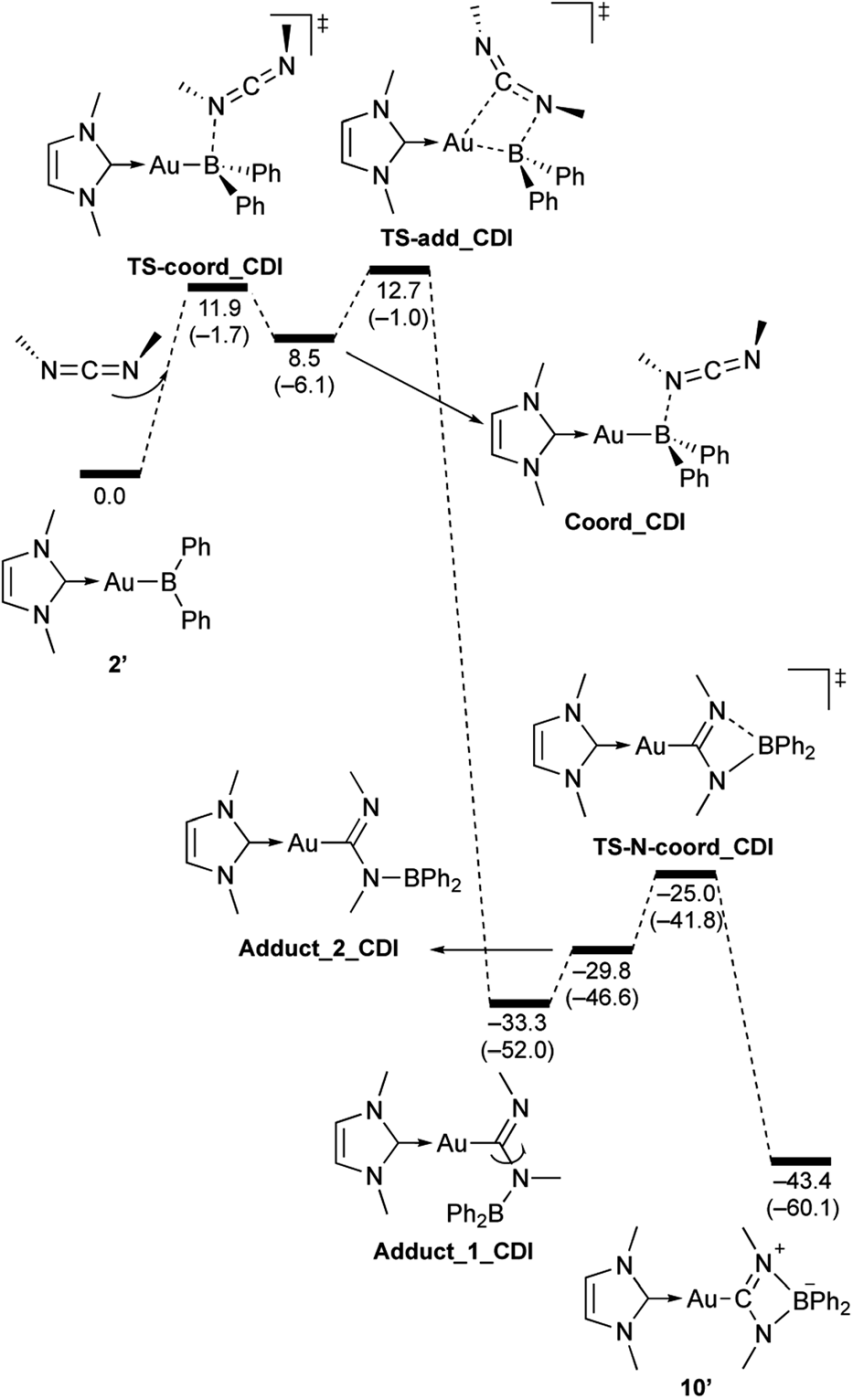
Calculated energy profile for the reaction of model compound 2′ with *N*,*N*-dimethylcarbodiimide to form 10′. Relative free energies and electronic energies (in parentheses) are given in kcal mol^−1^.

As described in the Introduction (*vide supra*), transition-metal and main-group metal boryl complexes generally exhibit nucleophilicity at the B center in reactions with polar functional groups such as C=O and C=N bonds to form a B–C bond, even though in some cases, the introduced boryl group subsequently migrates to the O or N atoms. In other words, the formation of the B–C bonds in 7–10*via* reactions of 2 with C=O and C=N species differs from the conventional reactivity of metal boryl compounds. Therefore, we performed DFT calculations at the PBE0/SDD (Au)/6-31G(d,p) (all other atoms) level of theory to analyze the reaction mechanism. This level of theory reproduces and explains well the experimental findings reported above, especially related to the relative reactivity of the diarylborylgold(i) complex toward different unsaturated substrates (see ESI† for more comments on the DFT method). For that purpose, a slightly simplified model complex (2′) with a methyl-substituted NHC ligand and two phenyl substituents instead of the 2,6-^*i*^Pr_2_C_6_H_3_-substituted NHC and two *o*-tolyl substituents in 2 were used. The calculated reaction mechanisms are illustrated in Schemes 7–10.

In the reaction of 2′ with benzaldehyde (Scheme 7), the initial coordination of benzaldehyde to 2′ affords the thermodynamically unstable intermediate Coord_PhCHO. The subsequent addition of the Au atom to the carbonyl carbon occurs *via* the four-membered ring transition state TS-add_PhCHO to form the stable intermediate Adduct_PhCHO under concomitant formation of Au–C and B–O bonds. This nucleophilic addition of Au is caused by enhanced reactivity of the Au–B bonding electrons and electrophilicity of B-coordinated carbonyl group as found in the HOMO/LUMO level of experimentally isolated pyridine adducts 3a,b. Barrierless C–O bond rotation of the resulting Adduct_PhCHO led to 7′, which is a model species for the experimentally obtained complex 7. It should be noted that the pathway for the addition of the B atom to the carbonyl carbon leads to a significantly higher activation energy than that for the pathway in Scheme 7 (Fig. S49†). The reaction of 2′ with benzophenone (Scheme 8) was also calculated to be a two-step reaction involving the initial coordination of the carbonyl group to the boron atom to form the intermediate Coord_Ph_2_CO and subsequent addition of the Au atom to the carbonyl carbon through the transition state TS-add_Ph_2_CO, which has a slightly higher activation energy than the corresponding step in Scheme 7. With *p*-fluorobenzoyl chloride as the substrate, the reaction becomes a three-step reaction (Scheme 9), involving an additional chloride migration as the third step; the overall activation energy is similar to that of the reaction in Scheme 8. In this reaction, the intermediate (Coord_ArCOCl) and transition state (TS-add_ArCOCl) similar to those in the reactions shown in Schemes 7 and 8 were found as stationary points. In the reaction with methyl-substituted carbodiimide (Scheme 10), a three-step reaction consisting of an initial coordination event to form an intermediate (Coord_CDI), the addition of the Au atom to the C=N carbon through a transition state (TS-add_CDI), and the formation of a B-containing four-membered ring was found. It should be noted here that the activation energy shown in Scheme 10 is remarkably lower than those of the reactions in Schemes 7–9, which reflects the much stronger coordination of the carbodiimide to the boron center compared to that of the carbonyl. Thus, all of the four reactions are initiated by the formation of relatively unstable C=O- and C=N-coordinated intermediates, followed by the migration of the nucleophilic gold center to attack the carbon atom of the carbonyl or carbodiimide functionality. This reactivity is similar to that of the release of one organic substituent from sp^3^-hybridized organoborate compounds.

#### Electronic character of the C=O- and C=N-coordinated intermediates and transition states prior to subsequent addition of Au atom to C=O and C=N carbons

**Fig. 6:**
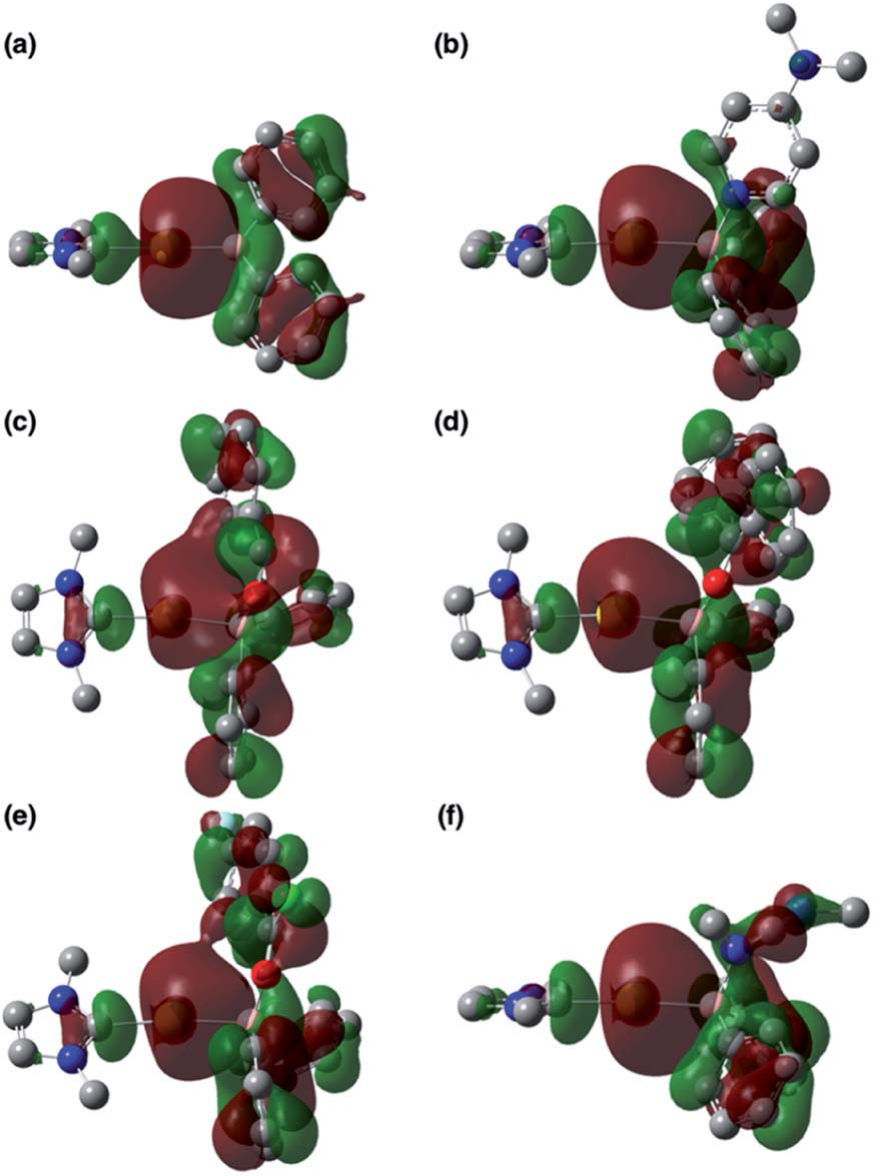
HOMOs of (a) 2′, (b) 3b′, (c) Coord_PhCHO, (d) Coord_Ph_2_CO, (e) Coord_ArCOCl, and (f) Coord_CDI.

**Fig. 7:**
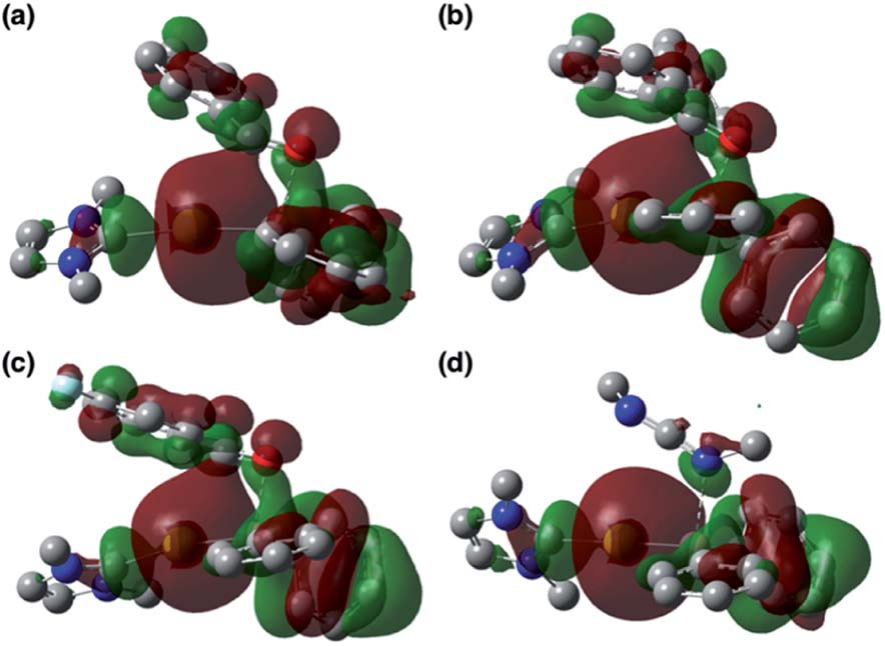
HOMOs of (a) TS_add_PhCHO, (b) TS_add_Ph_2_CO, (c) TS_add_ArCOCl, and (d) TS_add_CDI.

To clarify the origin of the characteristic reactivity of 2 toward C=O and C=N double bonds as an Au-centered nucleophile, the electronic properties of the reaction intermediates and the transition states were further analyzed using DFT calculations. The shapes of the HOMOs of the C=O- and C=N-coordinated intermediates Coord_X (X: PhCHO, Ph_2_CO, ArCOCl, and CDI) and those of model compounds 2′ and 3b′ are shown in Fig. 6, and the energy levels of the frontier orbitals are summarized in Table 1. As was confirmed in calculations on the experimental complex 2 (*vide supra*), the HOMO of the model diphenylborylgold complex 2′ contains a significant contribution from the Au–B σ-bond (Fig. 6a). The coordination of DMAP to 2′ to form the Lewis acid–base adduct 3b′ raises the energy level of the HOMO while retaining its Au–B σ-bond character (Fig. 6b and Table 1). These results indicate that the Au–B σ-bond plays an important role in the subsequent migration of the nucleophilic Au center. The higher reactivity of the Au–B σ-bond relative to the σ-B–C bonds in the Coord_X intermediates can be rationalized in terms of the higher energy of the B–Au bond relative to that of the B–C bond, because the Au(6s) orbital, which is used to form the Au–B bond, is much higher in energy than the C(2s/2p) orbitals. The narrower HOMO–LUMO gap in the intermediates Coord_X when compared with those of 2′ and 3b′ (Table 1) would induce the following nucleophilic addition of the Au atom. The addition of the Au atom to the C=O or C=N double bond occurs in a simple concerted process *via* the transition state TS_add_X (X: PhCHO, Ph_2_CO, ArCOCl, and CDI). The HOMOs of these transition states are illustrated in Fig. 7 and can be considered as the merger of the Au–B σ-bonding orbital and the π*-antibonding orbital of the C=O or C=N double bond, indicating a donor–acceptor interaction from the former to the latter. Thus, the Au center acts as an anionic nucleophile that attacks to the C atom of the C=O or C=N double bond in this transition state.

**Table 1:** Energy levels (a.u.) of the frontier orbitals and the corresponding energy gaps for 2′, 3b′, and the Lewis acid–base adducts

	HOMO	LUMO	Gap
2′ (DFT)	−0.19272	−0.03741	0.15531
3b′ (DFT)	−0.14199	−0.01673	0.12526
Coord_PhCHO	−0.17414	−0.06629	0.10785
Coord_Ph_2_CO	−0.17097	−0.06135	0.10962
Coord_ArCOCl	−0.17522	−0.07742	0.09780
Coord_CDI	−0.18104	−0.02006	0.16098

#### Reaction of 2 with Xyl-substituted isocyanide

**Scheme 11:**
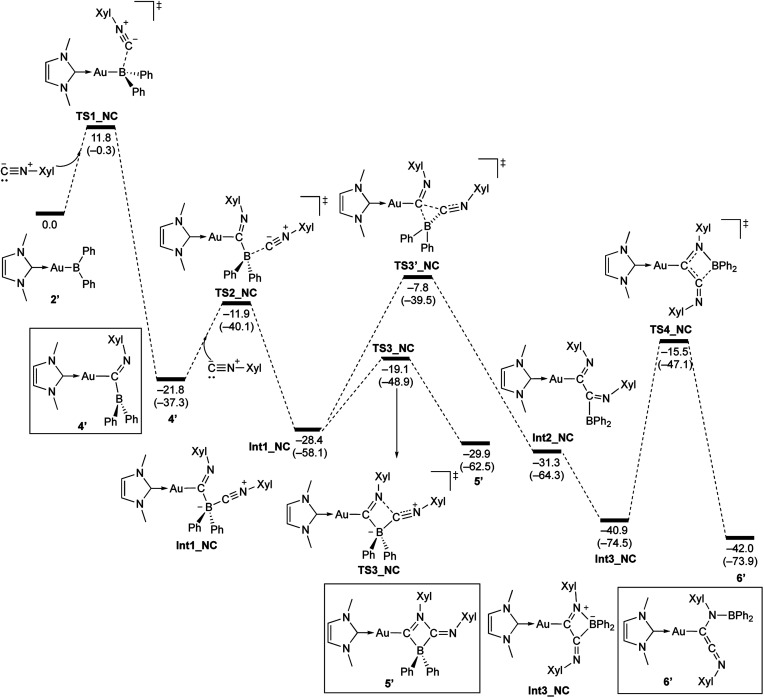
Calculated energy profile for the reaction of model compound 2′ with Xyl–NC to form 4′, 5′, and 6′. Relative free energies and electronic energies (in parentheses) are given in kcal mol^−1^.

The reaction of 2 with Xyl–NC was also theoretically investigated using DFT calculations in order to clarify the reaction mechanism(s) for the formation of 4–6 (Scheme 11). In the initial step, Xyl–NC attacks the Lewis-acidic B center of 2′ to form 4′ directly without the formation of a Lewis acid–base adduct (activation energy: 11.8 kcal mol^−1^). This is different from the reactions with carbonyls and carbodiimides, which involve the initial formation of a Lewis acid–base adduct. This difference arises from the ambiphilicity of the C atom of the isocyanide. The optimized structure of the resulting 4′ is in good agreement with the experimentally observed structure of 4. The subsequent reaction of 4′ with a second equivalent of Xyl–NC affords the Lewis acid–base adduct Int1_NC as a stable intermediate. The calculated activation energy (9.9 kcal mol^−1^) to the second transition state TS2_NC would be expected to be appreciably higher in the real system due to the additional substituents but is consistent with the isolability of 4 in the absence of the second equivalent of Xyl–NC. The intermediate Int1_NC is a common intermediate for the subsequent two reactions to give 5′ and 6′. The N atom of the imine moiety in Int1_NC engages in a nucleophilic attack on the activated C atom of the second isocyanide to furnish the four-membered compound 5′*via* the four-membered transition state TS3_NC. The slightly higher stability of 5′ relative to that of Int1_NC would explain why 5 can be isolated experimentally. The activation energy for the reverse reaction from 5′ to Int1_NC is 10.8 kcal mol^−1^, and the energy difference between these reactions is small. Thus, further reaction from the common intermediate Int1_NC is possible. In fact, Int1_NC can undergo insertion of the C atom of the second isocyanide into the B–C bond in Int1_NC to form the α-diimine intermediate Int2_NC*via* the three-membered transition state TS3′_NC (activation energy: 20.6 kcal mol^−1^). In this step, the bonding pair of electrons in the preformed B–C bond would act as a nucleophile to attack the C atom of the second isocyanide. Rotation of the newly formed C–C bond in Int2_NC induces the coordination of the Au-substituted imine functionality to the released diarylboryl moiety without a significant energy barrier to give another four-membered ring intermediate (Int3_NC). The subsequent dissociation of the diarylboryl moiety from the C atom of the second isocyanide furnishes the final product 6′ (activation energy: 25.4 kcal mol^−1^) under concomitant formation of the thermodynamically favorable B–N bond. The need for heating condition in the experiment to form 6 from 5 is consistent with the larger difference in energy between 5′ and TS3′_NC compared to the energy required for the formation of 5′. It should be noted here that the present pathways are consistent with the experimental results for the formation of 6-Mes. Thus, the DFT calculations clearly describe the reaction mechanism by which 4–6 are formed and why each compound can be isolated separately.

## Conclusions

In this study, we have reported the characteristic structure and absorption properties of diarylborylgold complex 2, as well as its reactivity toward polar multiple bonds. Complex 2 was obtained from the metathesis reaction of an NHC-ligated gold alkoxide complex with the previously reported tetra(*o*-tolyl)diborane(4). The linear complex 2 exhibits an orange color due to its narrower HOMO–LUMO gap compared to that of the previously reported complex (IPr)Au–Bpin. This result can be rationalized in terms of the electronic effect of the diarylboryl ligand. The reaction of 2 with isocyanides results in the insertion of the isocyanide into the B–Au bond; the product of this reaction can be treated with a second equivalent of isocyanide. Complex 2 also reacts with C=O- or C=N-containing compounds to furnish addition products *via* the formation of Au–C and B–O/N bonds, demonstrating the nucleophilicity of the Au center. DFT calculations revealed the detailed mechanisms that underline these reactions. In the reaction of 2 with C=O-/C=N-containing compounds, the heteroatom in the C=X moiety initially coordinates to the Lewis-acidic boron center in 2, followed by a migration of the gold atom from the sp^3^-hybridized boron atom to the carbon atom of the C=X moiety. The results of these DFT calculations, *i.e.*, the sequential process confirms the experimentally observed “nucleophilicity” of the gold center. In the reaction of 2 with isocyanides, reasonable pathways to the three different products were identified, and these are consistent with the experimental results. In their entirety, the results of this study have thus revealed various intricate features of the reactivity of a diarylboryl ligand in Au complexes.

## Conflicts of interest

There are no conflicts to declare.

## Supplementary Material

SC-012-D0SC05478J-s001

SC-012-D0SC05478J-s002

SC-012-D0SC05478J-s003
