# Supplementary material for: Nucleophilic reactivity of the gold atom in a diarylborylgold(i) complex toward polar multiple bonds
Source: Chem Sci. 2020 Nov 18;12(3):917–28. doi: 10.1039/d0sc05478j (PMC8179162; doi:10.1039/d0sc05478j)
Supplement: SC-012-D0SC05478J-s002 [file SC-012-D0SC05478J-s002.pdf]

Electronic Supplementary Material (ESI) for Chemical Science.
This journal is © The Royal Society of Chemistry 2020

Supplementary Information for

Diarylborylgold(I) Complex: Nucleophilic Reactivity of Gold Atom toward Polar Multiple Bond

Akane Suzuki,[†,1] Xueying Guo,[†,2] Zhenyang Lin,*[2] Makoto Yamashita*[1]

[1] Department of Molecular and Macromolecular Chemistry, Graduate School of Engineering, Nagoya University, Furo-cho, Chikusa-ku, Nagoya, 464-8603, Aichi, Japan

[2] Department of Chemistry, The Hong Kong University of Science and Technology, Clear Water Bay, Kowloon, Hong Kong

[†] A.S. and X.G. contributed equally to this work.

Experimental Section

General

All manipulations involving the air- and moisture-sensitive compounds were carried out under an argon atmosphere using standard Schlenk and glovebox (Korea KIYON, Korea and ALS Technology, Japan) technique. THF, Et_2O and *n*-hexane were purified by passing through a solvent purification system (Grass Contour). C_6D_6 was dried by distillation over sodium/benzophenone followed by vacuum transfer. Tetra(*o*-tolyl)diborane(4) (**1**)[1] and IPrAu(O*t*Bu)[2] were synthesized according to the literature. The nuclear magnetic resonance (NMR) spectra were recorded on a JEOL ECS-400 (399 MHz for ^{1}H, 100 MHz for ^{13}C, 128 MHz for ^{11}B) or a Bruker AVANCE III HD 500 spectrometers (500 MHz for ^{1}H, 125 MHz for ^{13}C). Chemical shifts (δ) are given by definition as dimensionless numbers and relative to ^{1}H or ^{13}C NMR chemical shifts of the residual C_6D_5H for ^{1}H (δ = 7.16) and C_6D_6 itself for ^{13}C (δ = 128.0). The ^{11}B NMR NMR spectra were referenced using an external standard of $BF_3\cdot OEt_2$ (δ_B = 0). The absolute values of the coupling constants are given in Hertz (Hz). Multiplicities are abbreviated as singlet (s), doublet (d), triplet (t), quartet (q), multiplet (m) and broad (br). NMR yield was determined by ^{1}H NMR spectrum of the crude product with phenanthrene as an internal standard. Melting points were determined on MPA100 OptiMelt (Tokyo Instruments, Inc.) and were uncorrected. Elemental analyses were performed on a Perkin Elmer 2400 series II CHN analyzer. High-resolution mass spectra were measured on a Bruker microTOF II mass spectrometer with an atmospheric pressure chemical ionization (APCI) probe. The starting material, (*o*-tol)$_2$B–B(*o*-tol)$_2$ was synthesized according to the reported procedure.[1]

Synthesis of 2, [IPrAuB(*o*-tol)$_2$]

In a glovebox, a solution of IPrAu(O*t*Bu) (200 mg, 0.304 mmol) and **1** (117 mg, 0.304 mmol) in diethyl ether (1.0 mL) was stirred at room temperature for 1 min. The resulting reaction mixture was cooled down to –35 ° C for recrystallization to give **2** as orange crystals (162 mg, 0.208 mmol, 68%).

^{1}H NMR (399 MHz, C_6D_6) δ 7.31 (t, *J* = 7 Hz, 2H), 7.19-7.16 (m, 2H), 7.15-7.09 (m, 6H), 7.06-7.02 (m, 4H), 6.32 (s, 2H, vinyl-H), 2.58 (sept, *J* = 7 Hz, 4H, CH of Dip), 2.38 (s, 6H, CH_3 of *o*-tolyl), 1.29 (d, *J* = 7 Hz, 12H, CH_3 of Dip), 1.07 (d, *J* = 7 Hz, 12H, CH_3 of Dip).

^{13}C NMR (100 MHz, C_6H_6) δ 23.97 (CH_3 of Dip), 24.07 (CH_3 of *o*-tolyl), 24.60 (CH_3 of Dip), 28.98 (CH of Dip),

122.67 (CH, vinyl), 124.11 (CH), 124.79 (CH), 127.78 (CH), 128.9 (CH), 130.32 (CH), 130.39 (CH), 135.29 (4°), 140.50 (4°), 146.03 (4°), 157.21 (br, 4°), 217.25 (4°, carbene)

^{11}B NMR (128 MHz, C_6H_6) δ 109 (br)

mp: 110-115 °C (decomp.)

Anal. Calc for $C_{41}H_{50}AuBN_2$: C, 63.24; H, 6.47; N, 3.60. Found: C, 63.15; H, 6.47; N, 3.56.

Procedure for estimation of NMR yield

In a glovebox, a solution of IPrAu(O^{t}Bu) (18.5 mg, 28.1 μmol), **1** (10.8 mg, 28.1 μmol), and phenanthrene (10.0 mg, 56.1 μmol) in C_6D_6 (600 μL) was stirred at room temperature for 1 min. The ^1H NMR spectrum measurement of the crude reaction mixture was performed to estimate NMR yield (Figure S4, **2**: 98% yield).

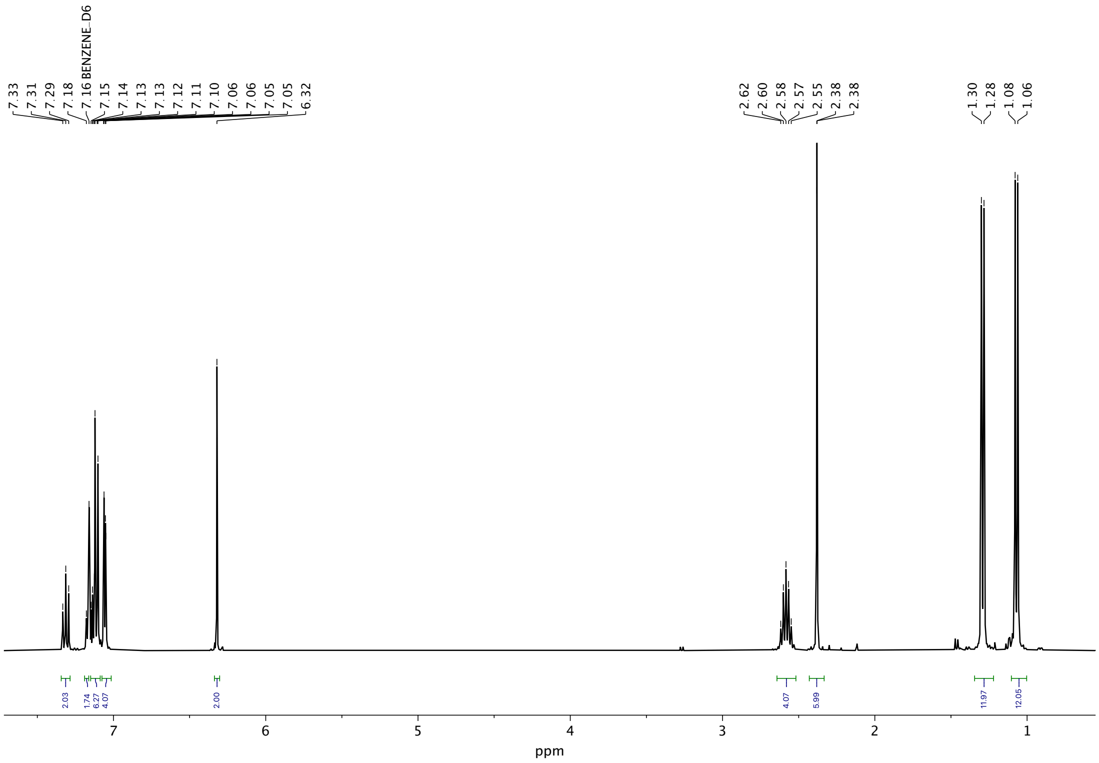

Figure S1. The ^1H NMR spectrum of **2** (†: C_6D_5H).

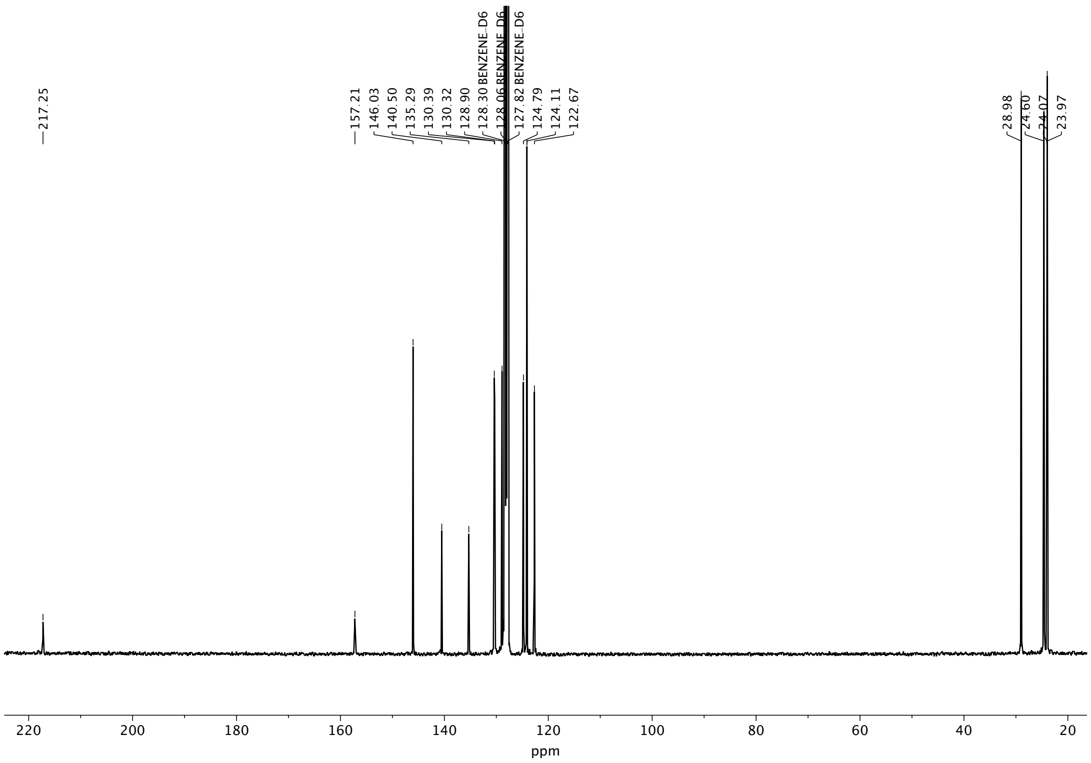

Figure S2. The ^{13}C NMR spectrum of **2**.

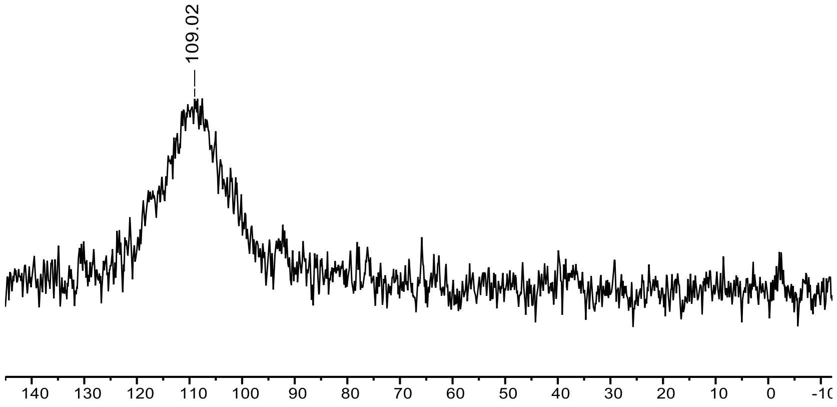

Figure S3. The ^{11}B NMR spectrum of **2**.

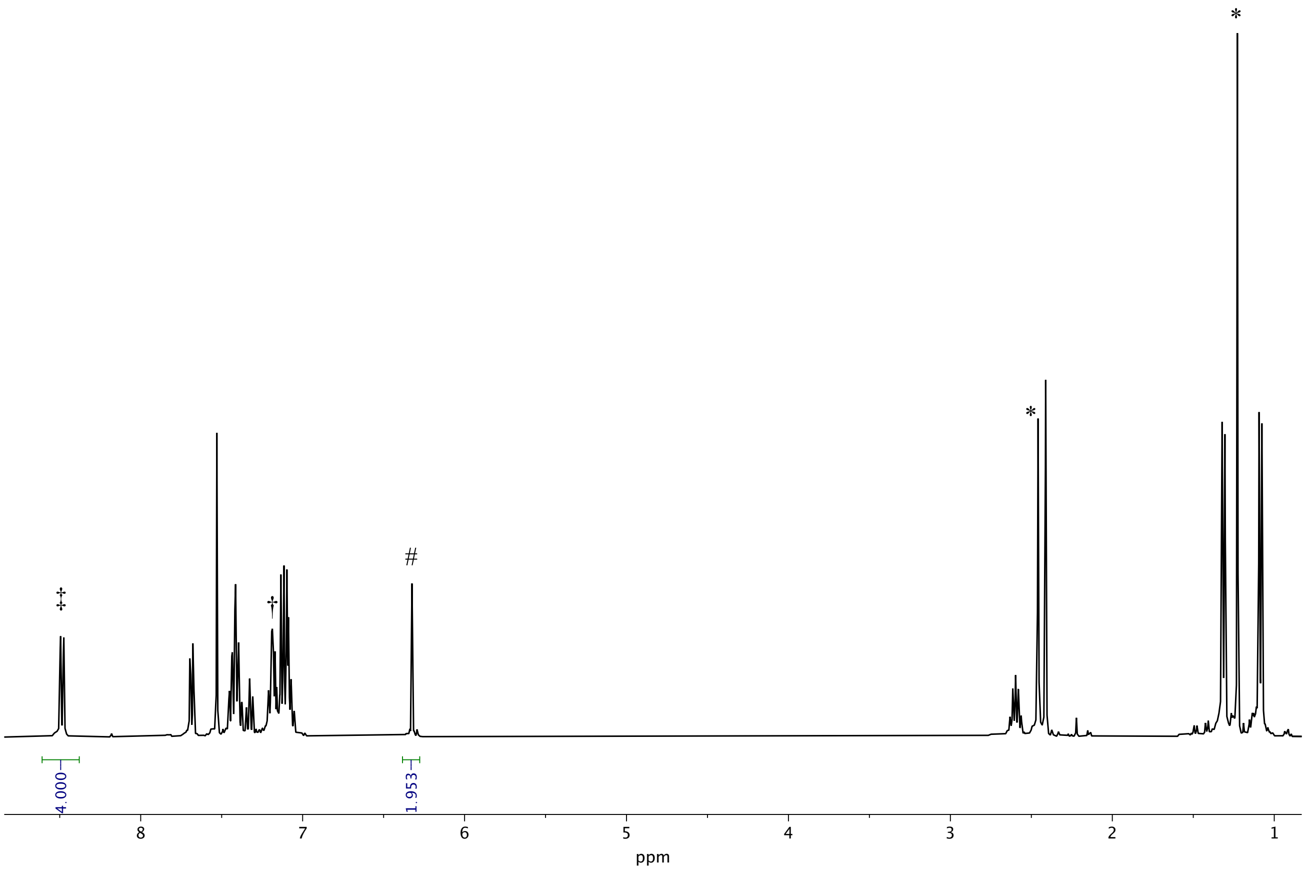

Figure S4. The ^{1}H NMR spectrum of the crude reaction mixture for the reaction of **1** with IPrAu(O^{t}Bu) to estimate NMR yield [*: n-hexane, †: C_6D_5H, #: **2** (98% yield), ‡: phenanthrene, *: B(O^{t}Bu)(*o*-tol)$_2$].

Synthesis of 3a, [IPrAu(py)(B(*o*-tol)$_2$)]

In a glovebox, benzene (1 mL) was added to a mixture of **2** (31.1 mg, 39.9 μmol) and pyridine (3.2 mg, 40 μmol). After stirring the reaction mixture at room temperature for 1 min, the resulting solution was concentrated under reduced pressure to dryness. The crude product was recrystallized by a vapor diffusion method using diethyl ether/hexane to give **3a** as purple crystals (20.1 mg, 59%).

^{1}H NMR (399 MHz, C_6D_6) δ 8.59 (d, J = 7 Hz, 2H), 7.29-7.21 (m, 4H), 7.15-7.03 (m, 8H), 6.82-6.73 (m, 2H), 6.69 (t, J = 7 Hz, 1H), 6.24-6.42 (m, 4H), 2.71 (sept, J = 7 Hz, 2H, CH of Dip), 2.70 (sept, J = 7 Hz, 2H, CH of Dip), 2.43 (s, 6H, CH_3 of *o*-tol), 1.27 (d, J = 7 Hz, 12H, CH_3 of Dip), 1.08 (d, J = 7 Hz, 12H, CH_3 of Dip).

^{13}C NMR (100 MHz, C_6H_6) δ 24.13 (CH_3, Dip), 24.30 (CH_3, Dip), 24.84 (CH_3, *o*-tolyl), 28.89 (CH, Dip), 122.05 (CH, vinyl), 123.53 (CH), 123.74 (br, CH), 123.99 (CH), 124.73 (CH), 128.35 (CH), 129.41 (CH), 129.89 (CH), 131.78 (CH), 135.91 (4°), 136.93 (br, CH), 141.47 (4°), 146.13 (4°), 150.93 (CH), 158.63 (br, 4°), 219.60 (4°, carbene)

^{11}B NMR (128 MHz, C_6H_6) δ 15 (br)

mp: 124-129 °C (decomp.)

Anal. Calc for $C_{46}H_{55}AuBN_3$: C, 64.41; H, 6.46; N, 4.90. Found: C, 64.18; H, 6.41; N, 4.86.

Procedure for estimation of NMR yield

In a glovebox, a solution of **2** (10.9 mg, 14.0 μmol), pyridine (111 μL, 126 mM, 14.0 μmol), and phenanthrene (10.0 mg, 56.1 μmol) in C_6D_6 (600 μL) was stirred at room temperature for 1 min. The ^1H NMR spectrum measurement of the crude reaction mixture was performed to estimate NMR yield (Figure S8, **3a**: 99% yield).

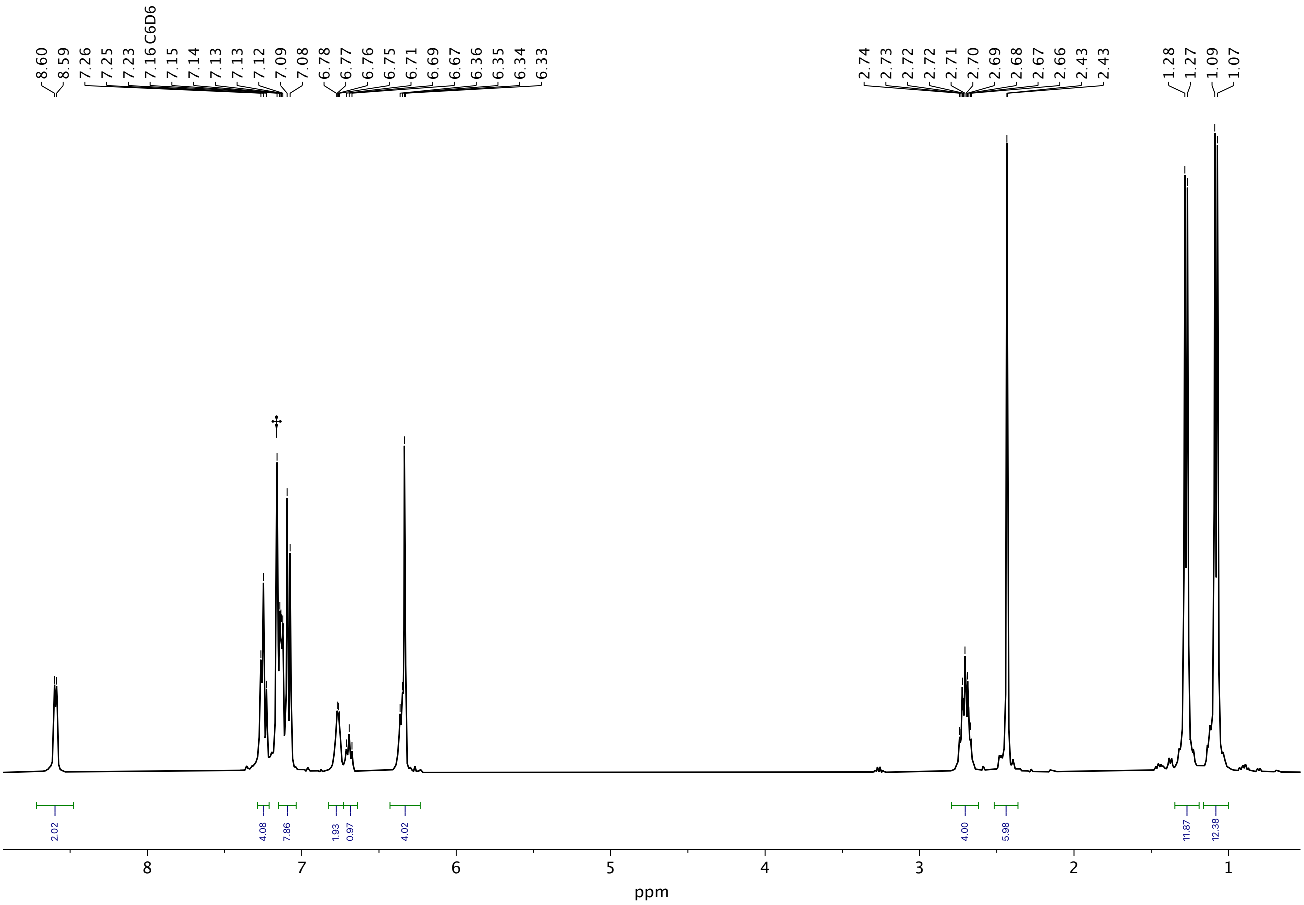

Figure S5. The ^1H NMR spectrum of **3a** (†: C_6D_5H).

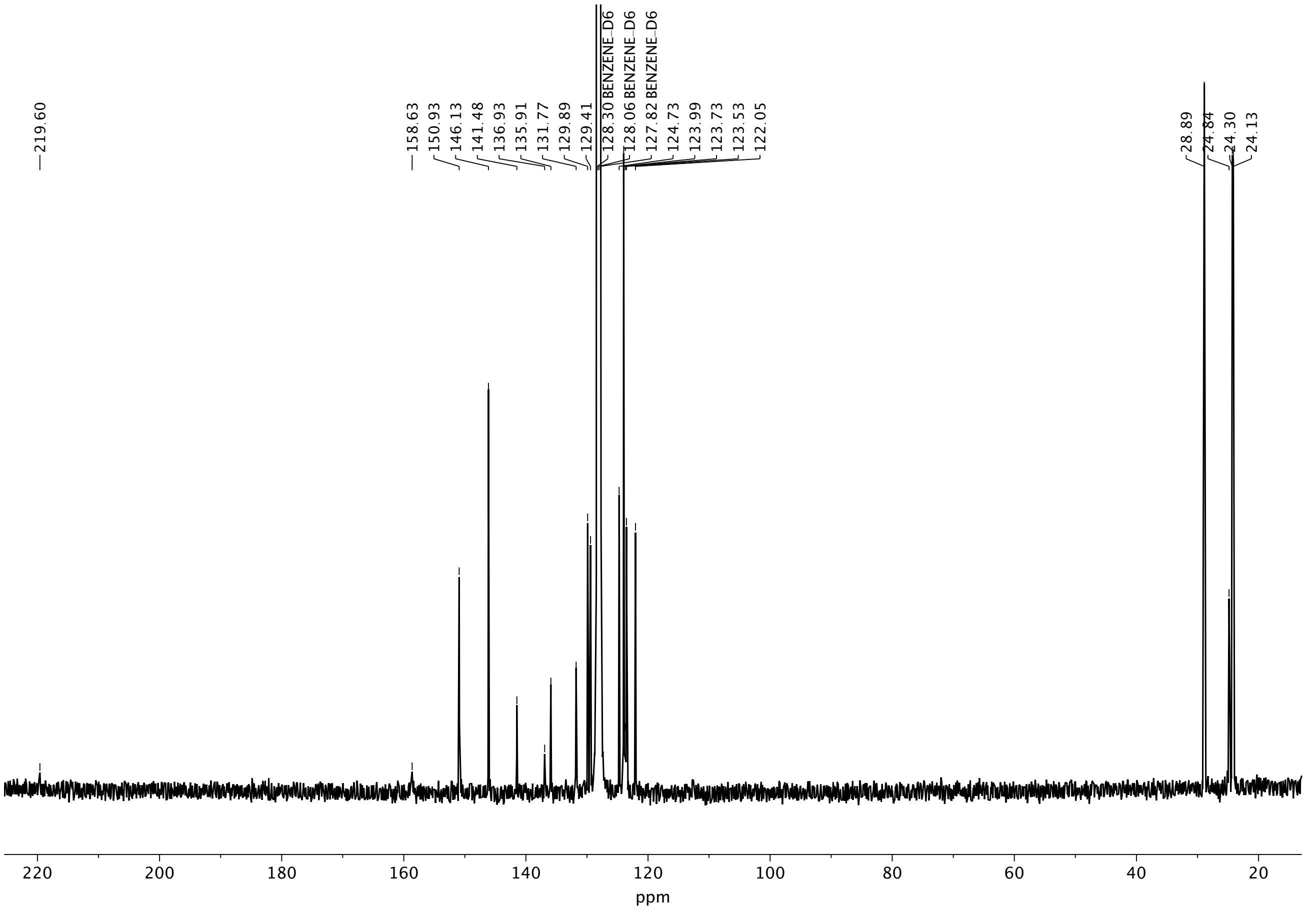

Figure S6. The ^{13}C NMR spectrum of **3a**.

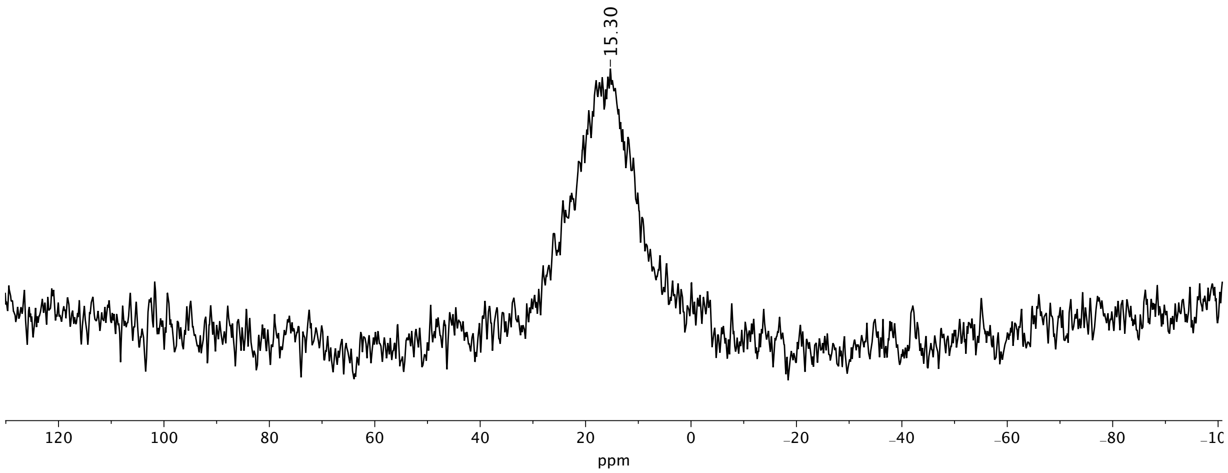

Figure S7. The ^{11}B NMR spectrum of **3a**.

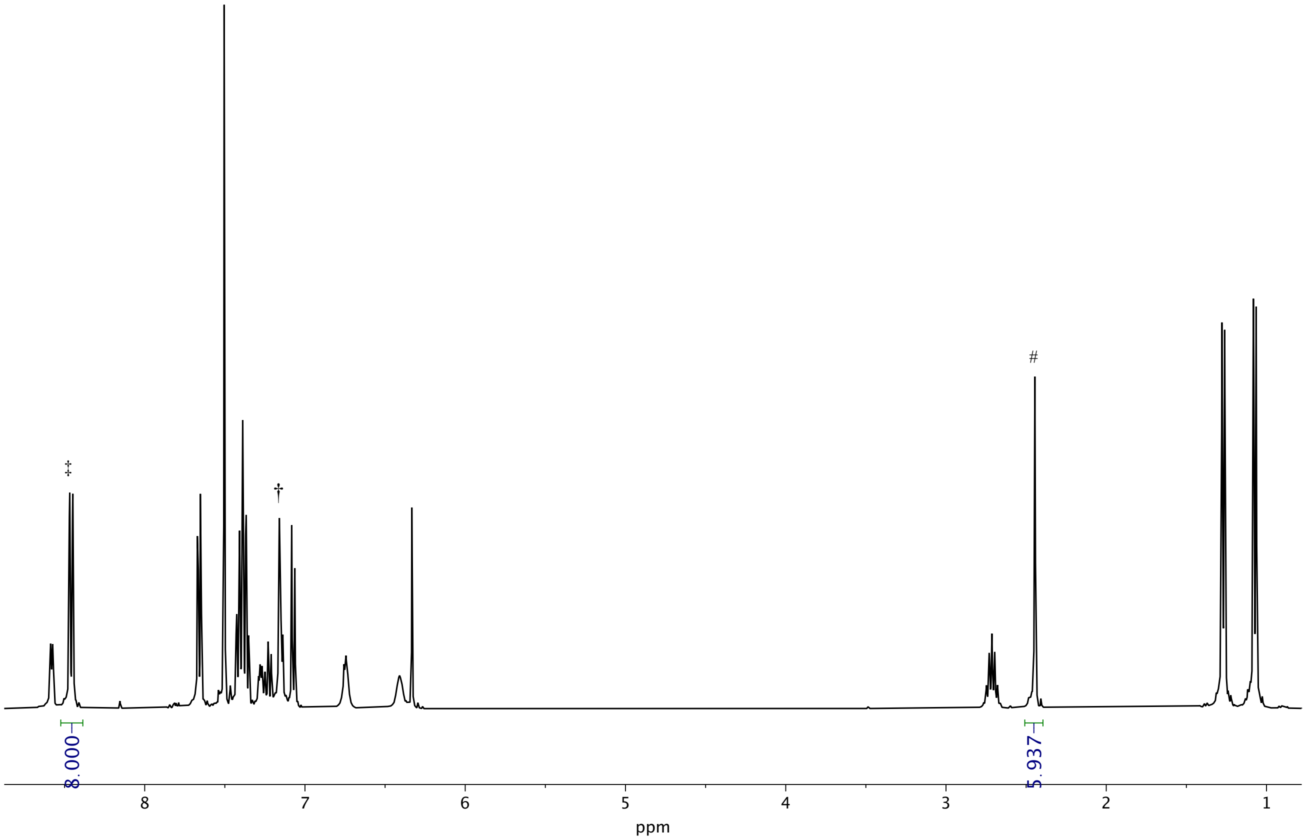

Figure S8. The ^{1}H NMR spectrum of the crude reaction mixture for the reaction of **2** with pyridine to estimate NMR yield [†: C_6D_5H, #: **3a** (99% yield), ‡: phenanthrene].

Synthesis of 3b, [IPrAu(DMAP)(B(*o*-tol)$_2$)]

In a glovebox, benzene (600 μL) was added to a mixture of **2** (15.0 mg, 19.3 μmol) and DMAP (2.4 mg, 20μmol). After stirring the reaction mixture at room temperature for 1 min, the color of the resulting reaction mixture changed from orange to red. In the first trial for recrystallization from reaction mixture by a slow evaporation of solvent, single crystals of **3b** suitable for single-crystal X-ray diffraction analysis was obtained, however, this recrystallization was not reproducible. During removal of solvent from the reaction mixture in the experiments for reproducibility, **3b** seemed to decompose. Therefore, we recorded the NMR spectra of freshly prepared **3b** in C_6D_6 (see following figures).

^{1}H NMR (399 MHz, C_6D_6) δ 8.28 (br s, 2H), 7.36-7.26 (m, 4H), 7.24-7.17 (m, 4H), 7.15-7.07 (m, 4H), 6.89 (br d, *J* = 5 Hz, 2H), 6.37 (s, 2H), 5.58 (br s, 2H), 2.80 (sept, *J* = 7 Hz, 4H, CH of Dip), 2.55 (s, 6H, NMe_2), 2.05 (br s, 6H, CH_3 of *o*-tol), 1.36 (d, *J* = 7 Hz, 12H, CH_3 of Dip), 1.11 (d, *J* = 7 Hz, 12H, CH_3 of Dip).

^{11}B NMR (128 MHz, C_6H_6) δ 11 (br)

Procedure for estimation of NMR yield

In a glovebox, a solution of **2** (10.9 mg, 14.0 μmol), DMAP (172 μL, 819 mM, 14.0 μmol), and phenanthrene (10.0 mg, 56.1 μmol) in C_6D_6 (600 μL) was stirred at room temperature for 1 min. The ^{1}H NMR spectrum measurement of the crude reaction mixture was performed to estimate NMR yield (Figure S11, **3b**: 88% yield).

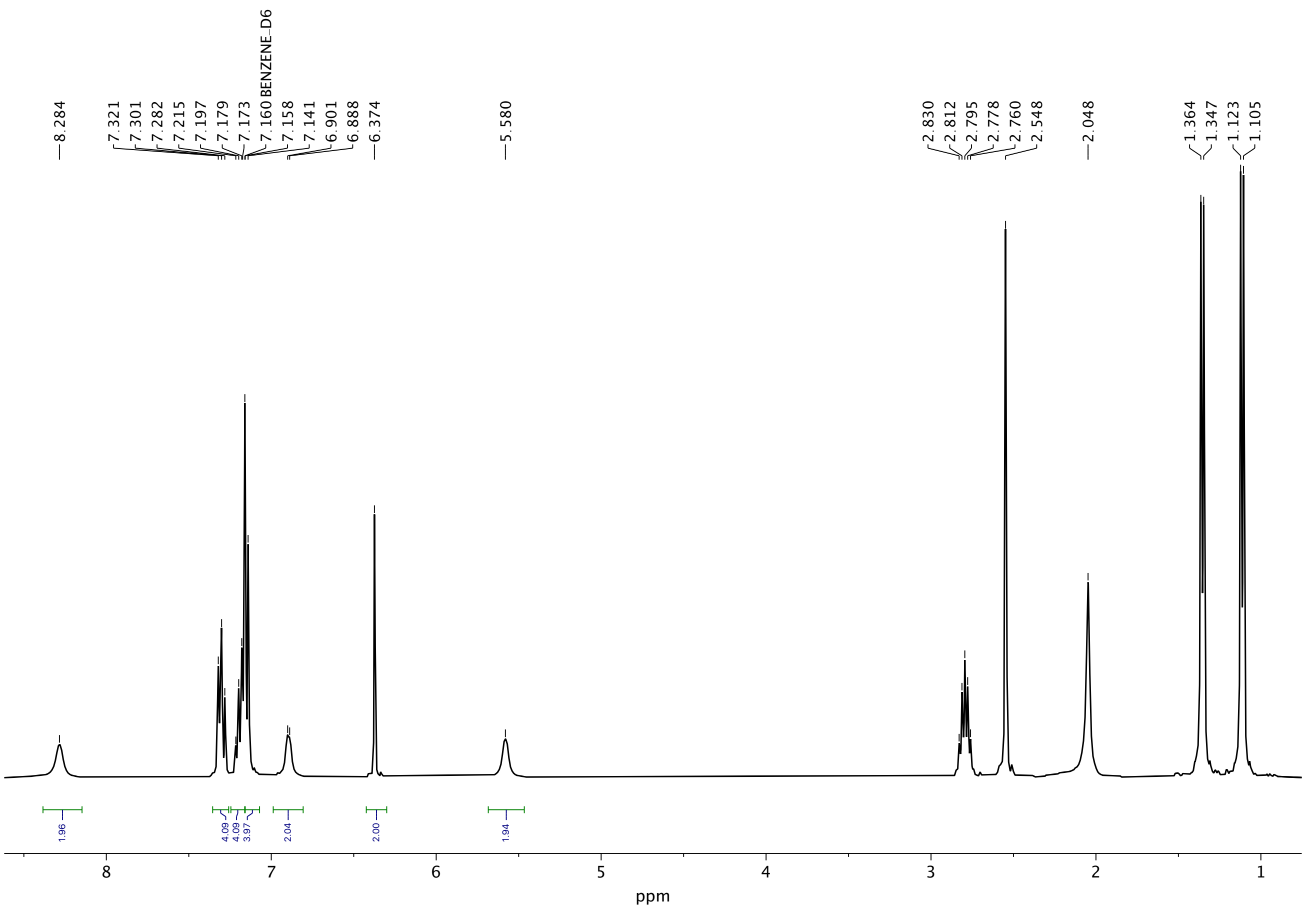

Figure S9. The ^{1}H NMR spectrum of *in-situ* generated **3b**.

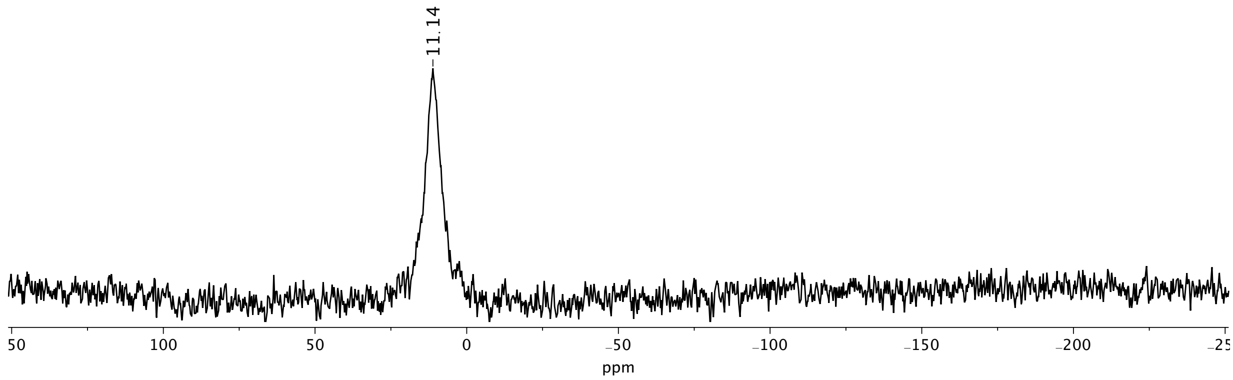

Figure S10. The ^{11}B NMR spectrum of *in-situ* generated **3b**.

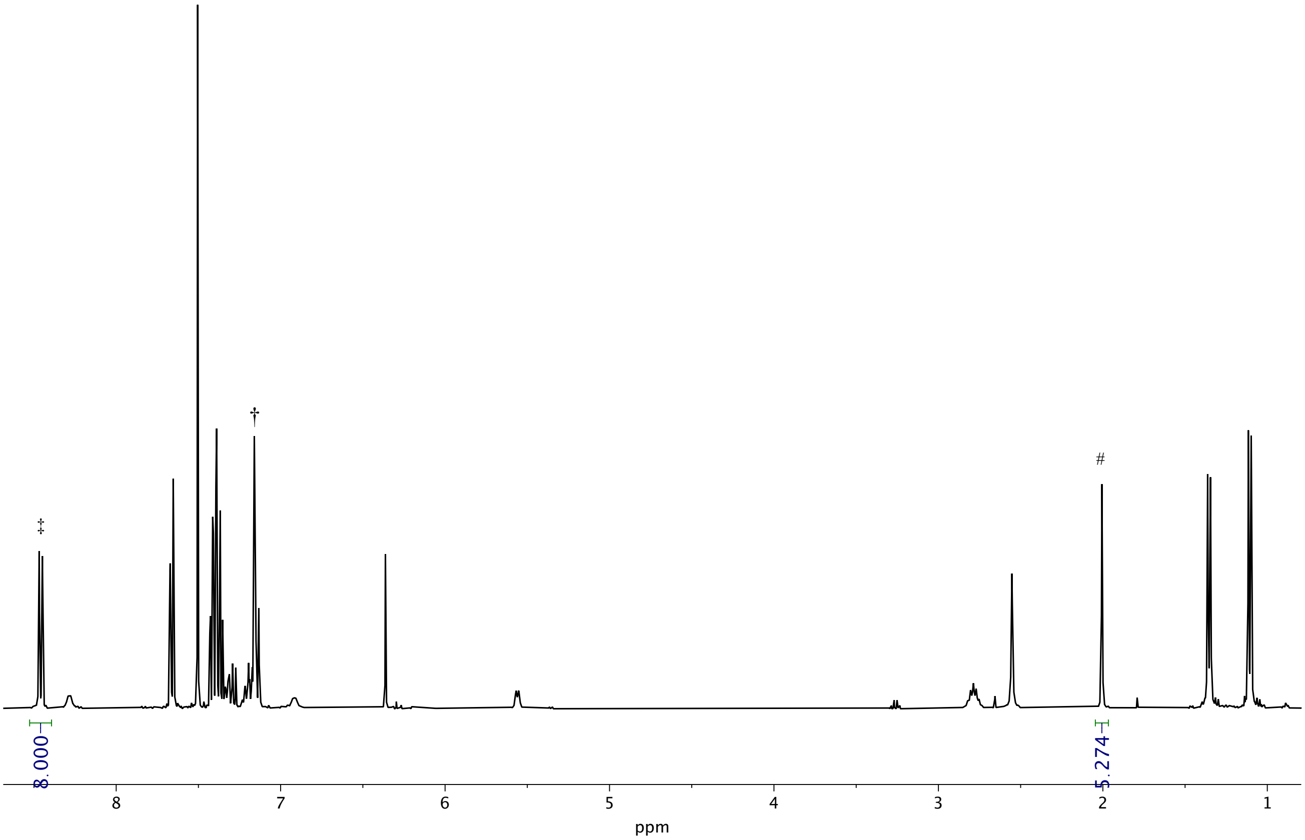

Figure S11. The ^{1}H NMR spectrum of the crude reaction mixture for the reaction of **2** with DMAP to estimate NMR yield [†: C_6D_5H, #: **3b** (88% yield), ‡: phenanthrene].

Synthesis of 4

In a glovebox, a benzene (500 µL) solution of 2,6-dimethylphenyl isocyanide (3.8 mg, 29 µmol) was slowly dropped to a benzene (500 µL) solution of **2** (22.6 mg, 29.0 µmol) at room temperature. After stirring the reaction mixture at room temperature for 1 min, the resulting solution was concentrated under reduced pressure and the crude product was recrystallized by a vapor diffusion method using diethyl ether/hexane to give **4** as yellow crystals (22.3 mg, 84%).

^{1}H NMR (399 MHz, C_6D_6) δ 7.29 (t, *J* = 7 Hz, 2H), 7.24-6.17 (m, 4H), 7.11-7.07 (m, 2H), 7.07-7.04 (m, 4H), 7.04-6.99 (m, 2H), 6.89-6.83 (m, 2H), 6.82-6.75 (m, 1H), 6.19 (s, 2H, vinyl-H), 2.48 (s, 6H, CH_3 of Xyl), 2.36 (sept, *J* = 7 Hz, 4H, CH of Dip), 1.93 (s, 6H, CH_3 of *o*-tol), 1.03 (d, *J* = 7 Hz, 12H, CH_3 of Dip), 0.95 (d, *J* = 7 Hz, 12H, CH_3 of Dip).

^{13}C NMR (100 MHz, C_6H_6) δ 18.92 (CH_3 of Xyl), 23.95 (CH_3 of *o*-tolyl), 24.10 (CH_3 of Dip), 24.16 (CH_3 of Dip), 29.02 (CH of Dip), 121.54 (CH), 123.06 (CH), 124.47 (CH), 125.92 (4°), 126.69 (CH), 127.12 (4°), 128.18 (CH), 128.35 (4°), 129.33 (CH), 130.88 (CH), 134.51 (4°), 137.49 (CH), 142.74 (4°), 145.52 (4°), 152.05 (4°), 153.61 (4°, C=N), 197.69 (4°, carbene).

^{11}B NMR (128 MHz, C_6H_6) δ 11 (br)

mp: 118-120 °C (decomp.)

Anal. Calc for $C_{50}H_{59}AuBN_3$: C, 66.01; H, 6.54; N, 4.62. Found: C, 66.05; H, 6.37; N, 4.51.

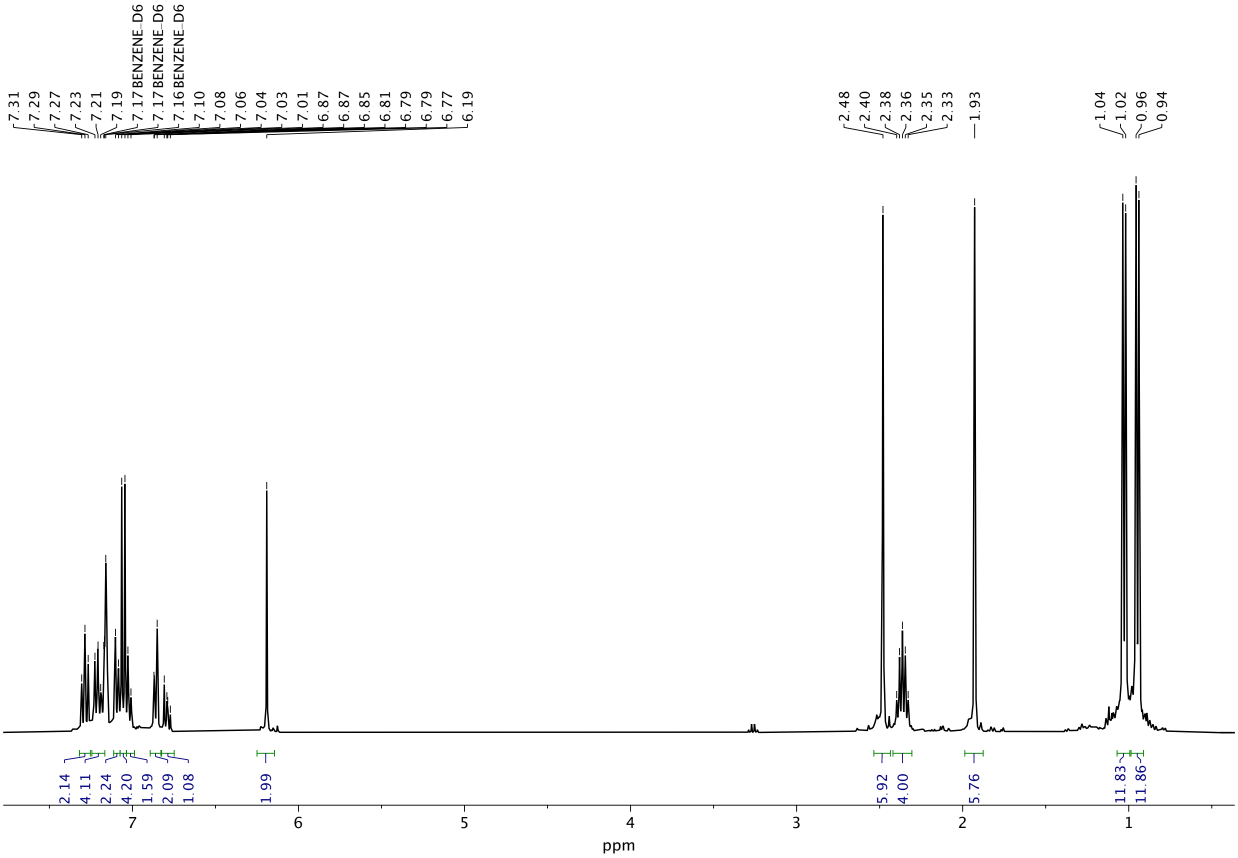

Figure S12. The ^{1}H NMR spectrum of **4**.

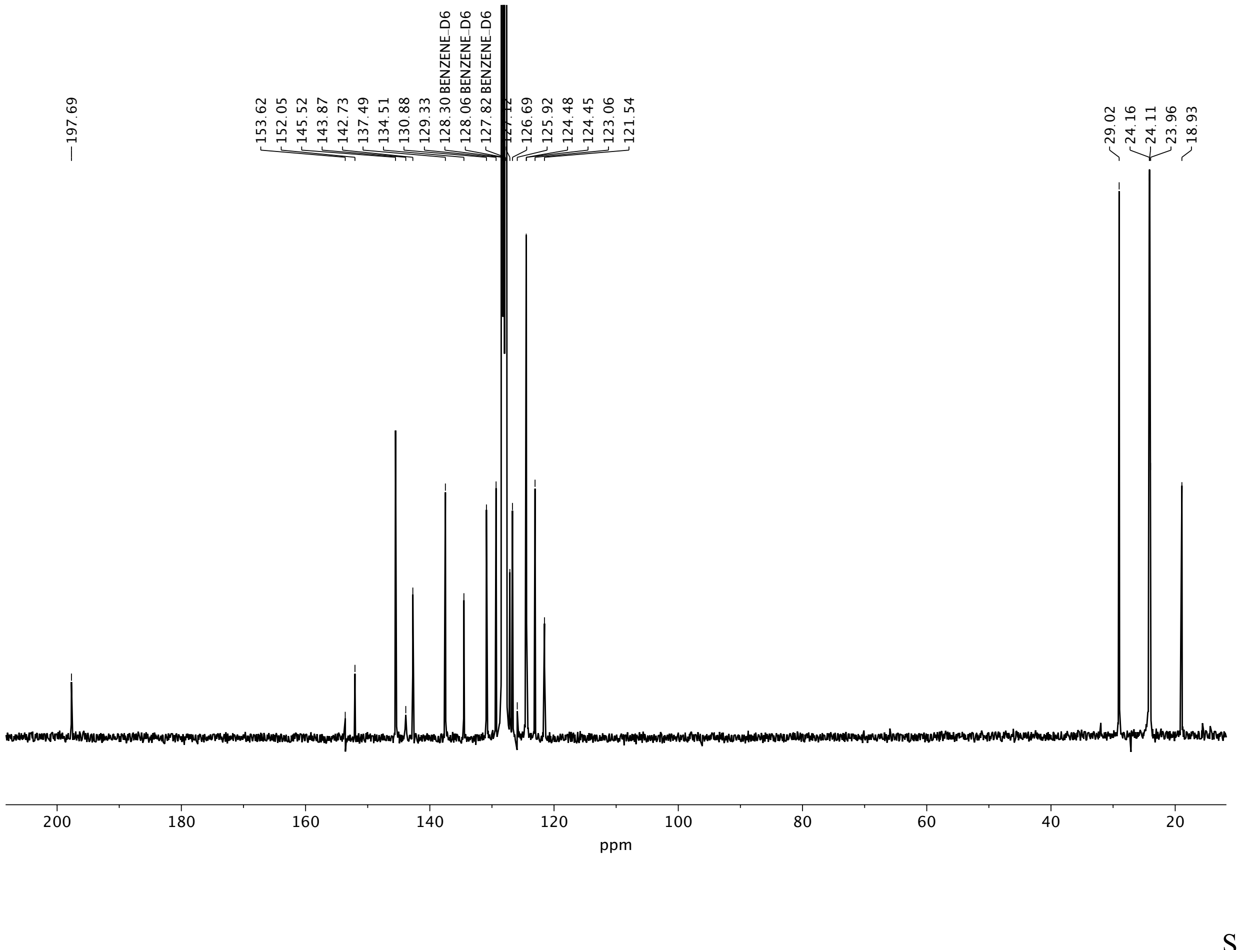

Figure S13. The ^{13}C NMR spectrum of **4**.

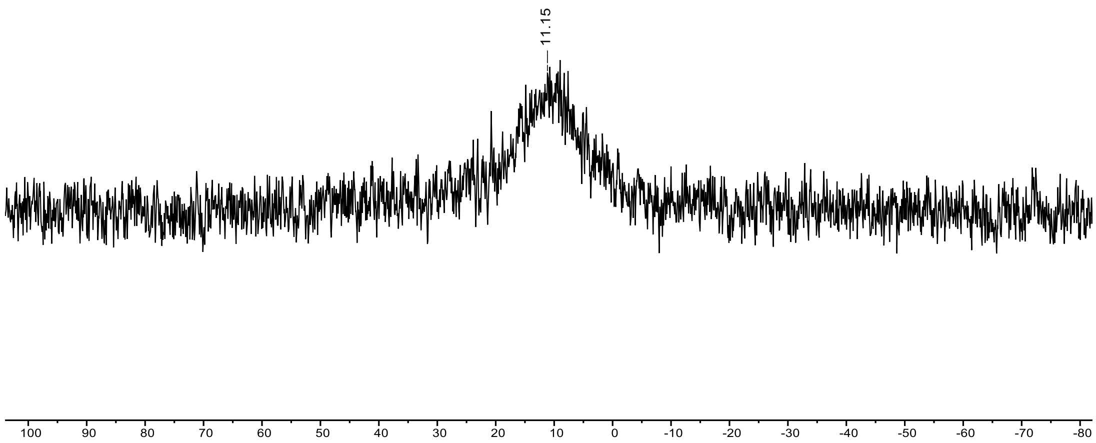

Figure S14. The ^{11}B NMR spectrum of **4**.

Synthesis of 5

In a glovebox, a diethyl ether (500 μL) solution of 2,6-dimethylphenyl isocyanide (11.3 mg, 86.2 μmol) was added dropwise slowly to a diethyl ether (500 μL) solution of **2** (33.5 mg, 43.0 μmol) at room temperature. After stirring the solution at room temperature for 1 min, the generated precipitates were washed with hexane and concentrated to give **5** as yellow powder (15.2 mg, 16.7 mmol, 39%).

^{1}H NMR (399 MHz, C_6D_6) δ 7.81 (t, J = 5 Hz, 2H), 7.23-7.18 (m, 6H), 7.01 (t, J = 5 Hz, 2H), 6.98-6.91 (m, 7H), 6.85 (t, J = 8 Hz, 1H), 6.80 (d, J = 8 Hz, 2H), 6.15 (s, 2H), 2.33 (sep, J = 7 Hz, 4H), 2.13 (s, 6H), 2.12 (s, 6H), 1.81 (s, 6H), 1.09 (d, J = 7 Hz, 12H), 0.99 (d, J = 7 Hz, 12H).

^{13}C NMR (100 MHz, C_6H_6) δ 18.59 (CH_3 of Xyl), 18.80 (CH_3 of Xyl), 23.65 (CH_3 of *o*-tolyl), 23.81 (CH_3 of Dip), 24.42 (CH_3 of Dip), 28.90 (CH of Dip), 122.60 (CH), 123.18 (CH), 124.18 (CH), 124.39 (CH), 125.16 (CH), 127.48 (CH), 127.57 (4°), 128.28 (CH), 128.35 (CH), 129.28 (CH), 130.92 (CH), 134.18 (4°), 134.53 (4°), 134.63 (CH), 141.76 (4°), 142.78 (4°), 145.41 (4°), 147.48 (4°), 194.27 (4°, carbene)

^{11}B NMR (128 MHz, C_6H_6) δ 4 (br)

Anal. Calc for $C_{59}H_{68}AuBN_4{\cdot}Et_2O$: C, 67.86; H, 7.05; N, 5.02. Found: C, 67.79; H, 6.73; N, 5.00.

Procedure for estimation of NMR yield

In a glovebox, a C_6D_6 solution of 2,6-dimethylphenyl isocyanide (368 μL, 76.2 mM, 28.1 μmol) was slowly added dropwise to a C_6D_6 (232 μL) solution of **2** (10.9 mg, 14.0 μmol) and phenanthrene (10.0 mg, 56.1 μmol) at room temperature. After stirring the reaction mixture at room temperature for 1 min, the ^{1}H NMR spectrum measurement of the crude reaction mixture was performed to estimate NMR yield (Figure S18, **5**: 99% yield).

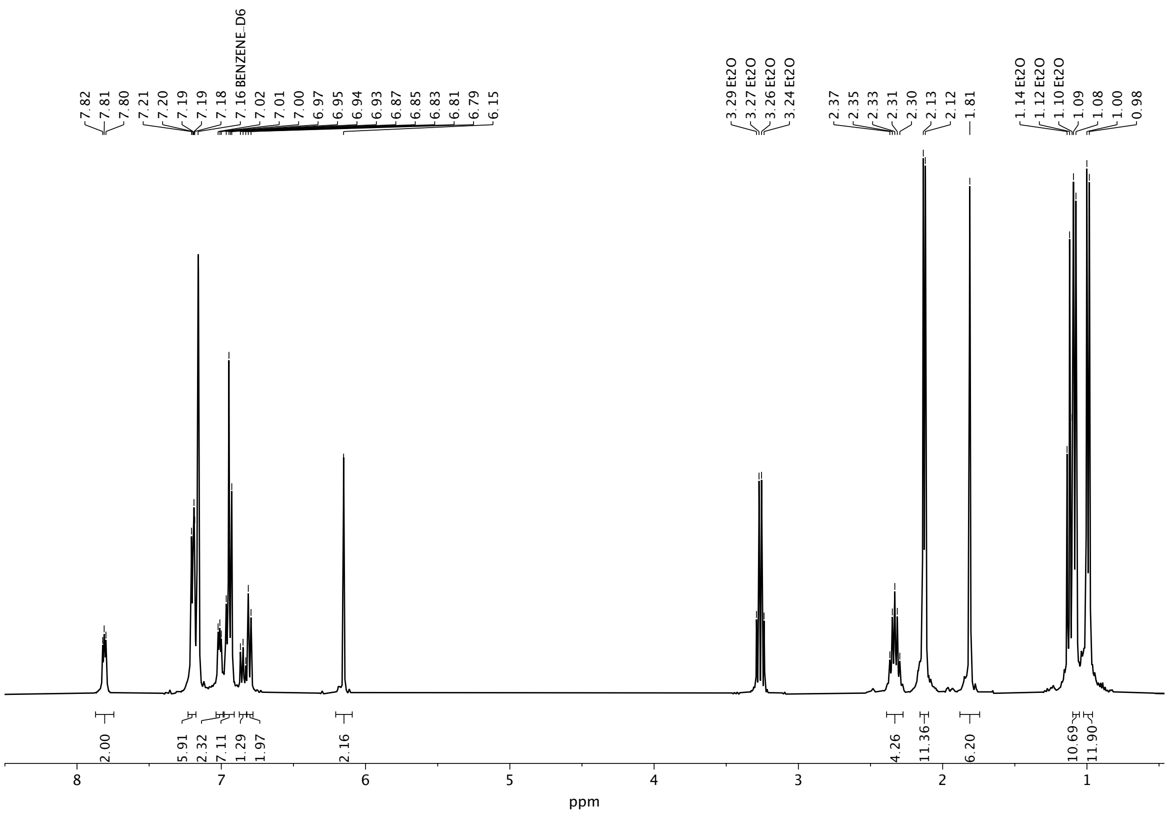

Figure S15. The ^{1}H NMR spectrum of **5**.

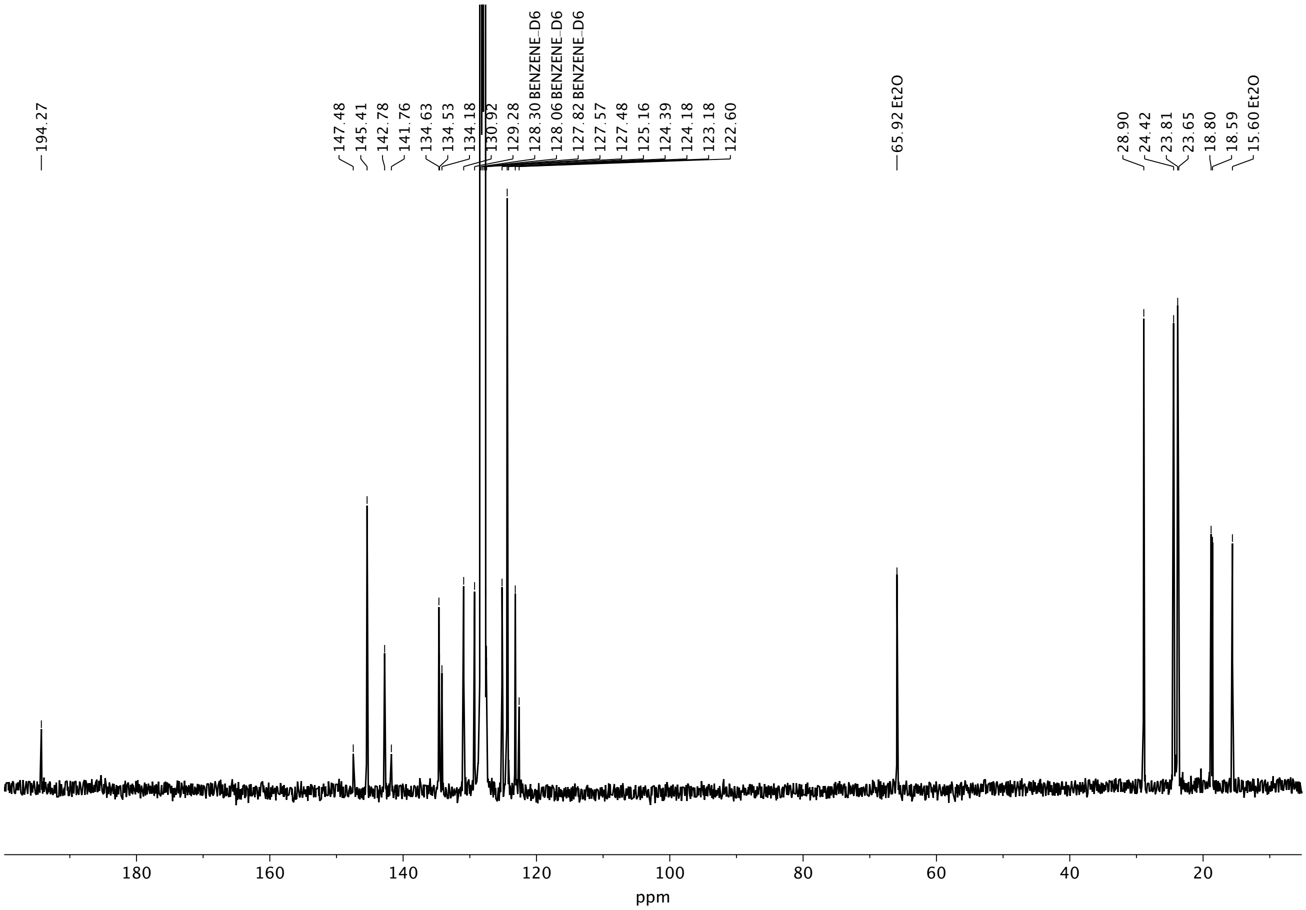

Figure S16. The ^{13}C NMR spectrum of **5**.

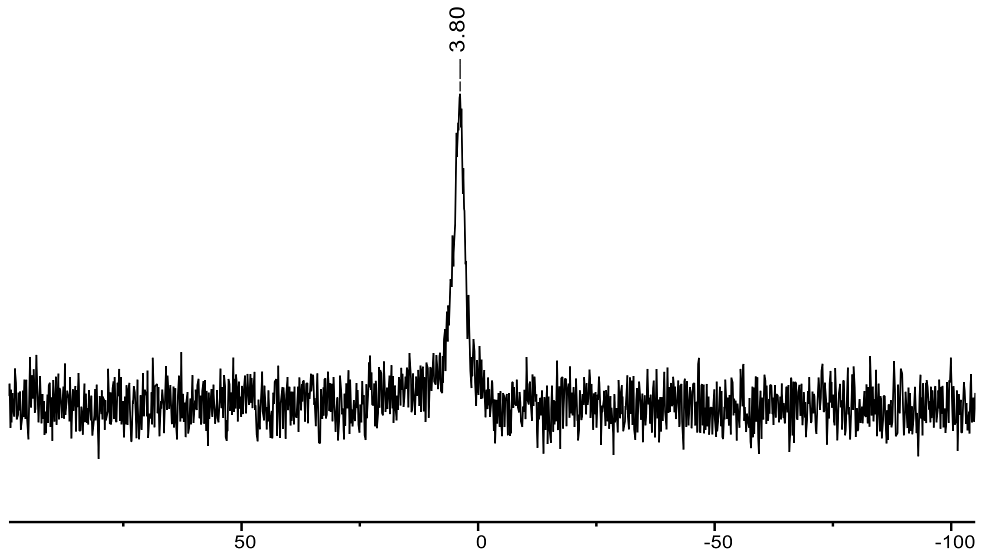

Figure S17. The ^{11}B NMR spectrum of **5**.

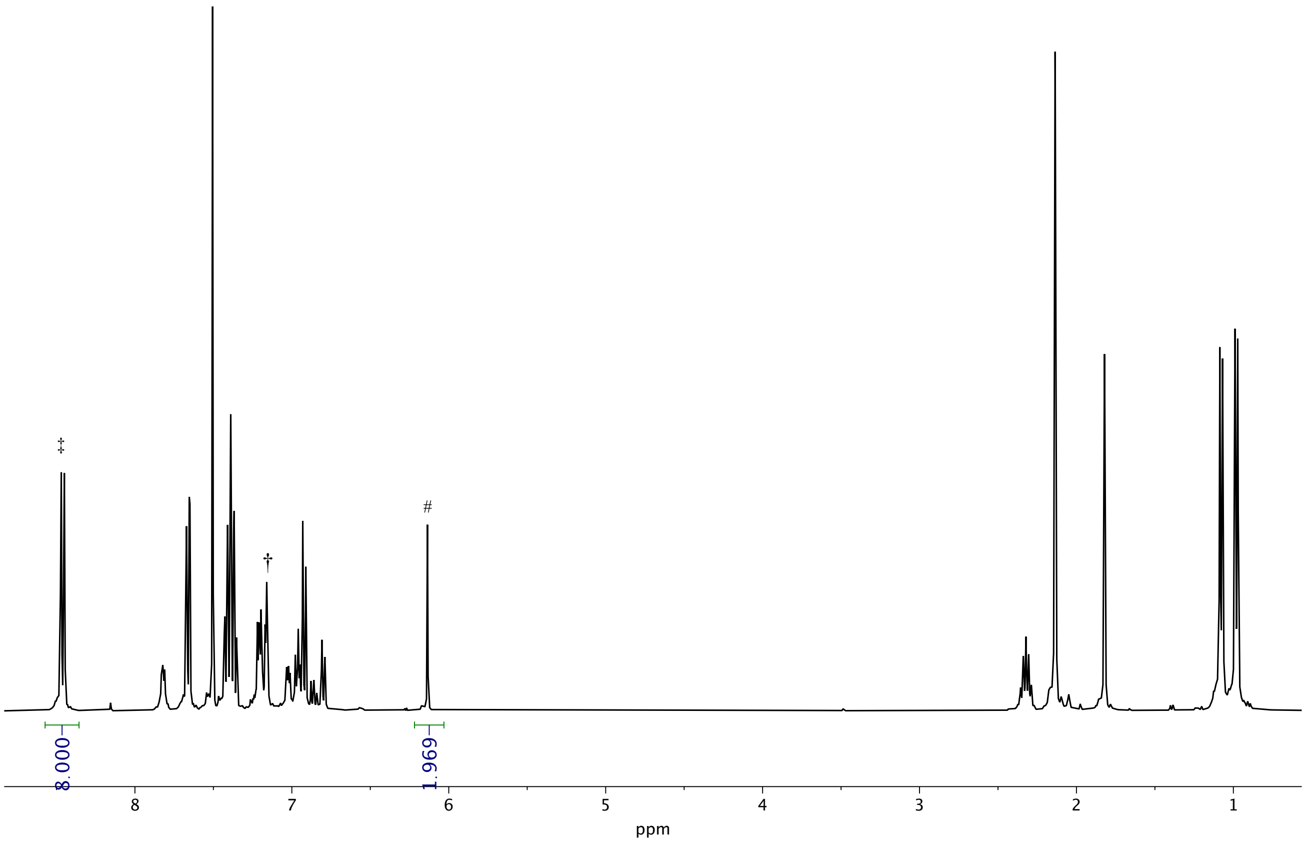

Figure S18. The ^{1}H NMR spectrum of the crude reaction mixture for the reaction of **2** with 2 eq. XylNC [†: C_6D_5H, #: **5** (99% yield), ‡: phenanthrene].

Synthesis of 6

In a glovebox, 10 mL J young tube was charged with **5** (29.4 mg, 28.2 μmol) and benzene (2.0 mL). After stirring the solution at 70 °C for 3 h, the resulting reaction mixture was concentrated under reduced pressure and the crude product was recrystallized by a vapor diffusion method with toluene to give **6** as green crystals (11.1 mg, 10.7 μmol, 38%).

^{1}H NMR (399 MHz, C_6D_6) δ 7.48 (d, *J* = 7 Hz, 1H), 7.25-7.17 (m, 2H), 7.16-7.11 (m, 4H), 6.98 (d, *J* = 8 Hz, 4H), 6.89-6.76 (m, 11H), 6.70 (d, *J* = 7 Hz, 2H), 6.20 (s, 2H), 2.54 (sept, *J* = 7 Hz, 4H), 2.48 (s, 6H), 2.32 (s, 3H), 2.16 (s, 3H), 1.94 (s, 6H), 1.29 (d, *J* = 7 Hz, 12H), 1.03 (d, *J* = 7 Hz, 12H).

^{13}C NMR (100 MHz, Toluene-d_8, -30 °C) δ 19.04 (CH_3 of Xyl), 20.31 (CH_3 of Xyl), 23.03 (CH_3 of o-tol), 23.83 (CH_3 of o-tol), 23.88 (CH_3 of Dip), 24.35 (CH_3 of Dip), 29.01 (CH of Dip), 97.43 (4°, C=*C*=N), 122.65 (CH), 123.28 (CH), 124.09 (CH), 124.25 (CH), 124.56 (CH), 124.69 (CH), 126.45 (CH), 127.46 (CH), 128.07 (CH), 128.40 (CH), 128.46 (CH), 128.92 (CH), 129.65 (CH), 130.65 (CH), 130.76 (4°), 133.12 (CH), 133.50 (CH), 134.99 (4°), 135.56 (4°), 139.15 (4°), 140.92 (4°), 142.25 (4°), 145.61 (4°), 148.75 (4°), 193.13 (4°, Au–*C*=C). 193.94 (4°, carbene).

^{11}B NMR (128 MHz, C_6H_6) δ 43 (br)

mp: 160-162 °C (decomp.)

Anal. Calc for $C_{59}H_{68}AuBN_4$: C, 68.07; H, 6.58; N, 5.38. Found: C, 68.36; H, 6.22; N, 5.42.

Procedure for estimation of NMR yield

In a glovebox, a C_6D_6 solution of 2,6-dimethylphenyl isocyanide (368 μL, 76.2 mM, 28.1 μmol) was slowly dropped to a C_6D_6 (232 μL) solution of **2** (10.9 mg, 14.0 μmol) and phenanthrene (10.0 mg, 56.1 μmol) at room temperature. After stirring the reaction mixture at 70 °C for 3 h, the ^{1}H NMR spectrum measurement of the crude reaction mixture was performed to estimate NMR yield (Figure S22, **6**: 77% yield).

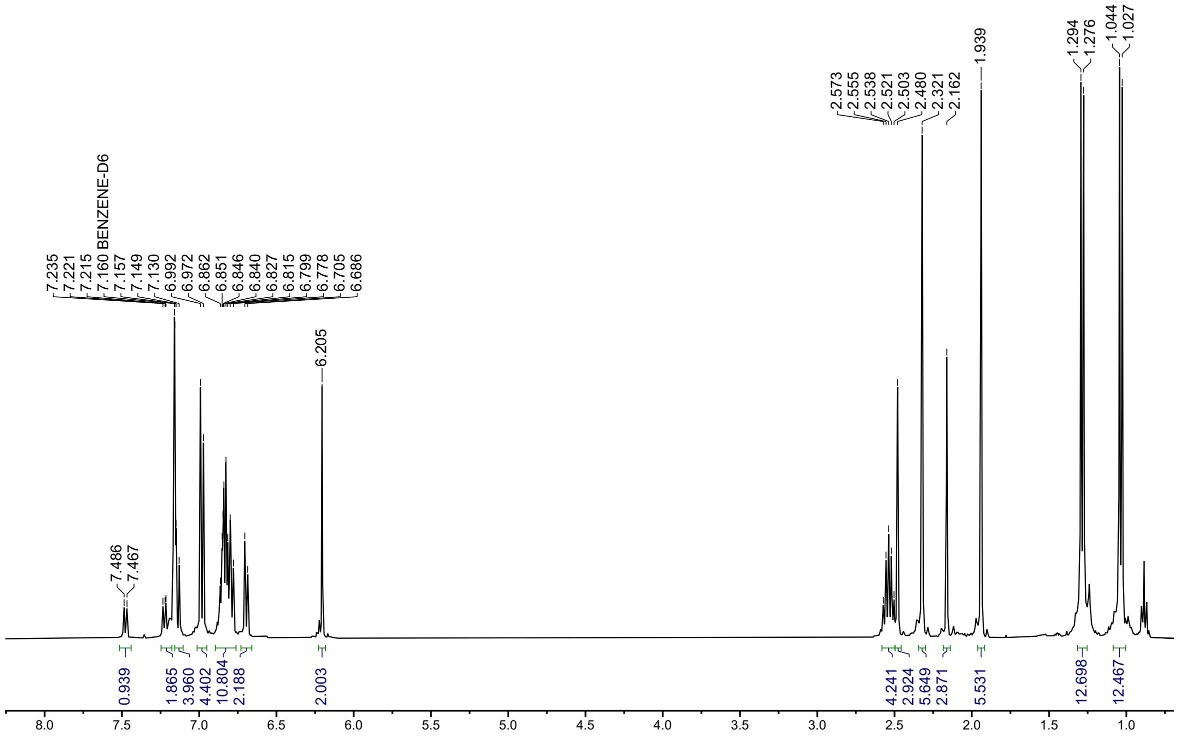

Figure S19. The ^{1}H NMR spectrum of **6**.

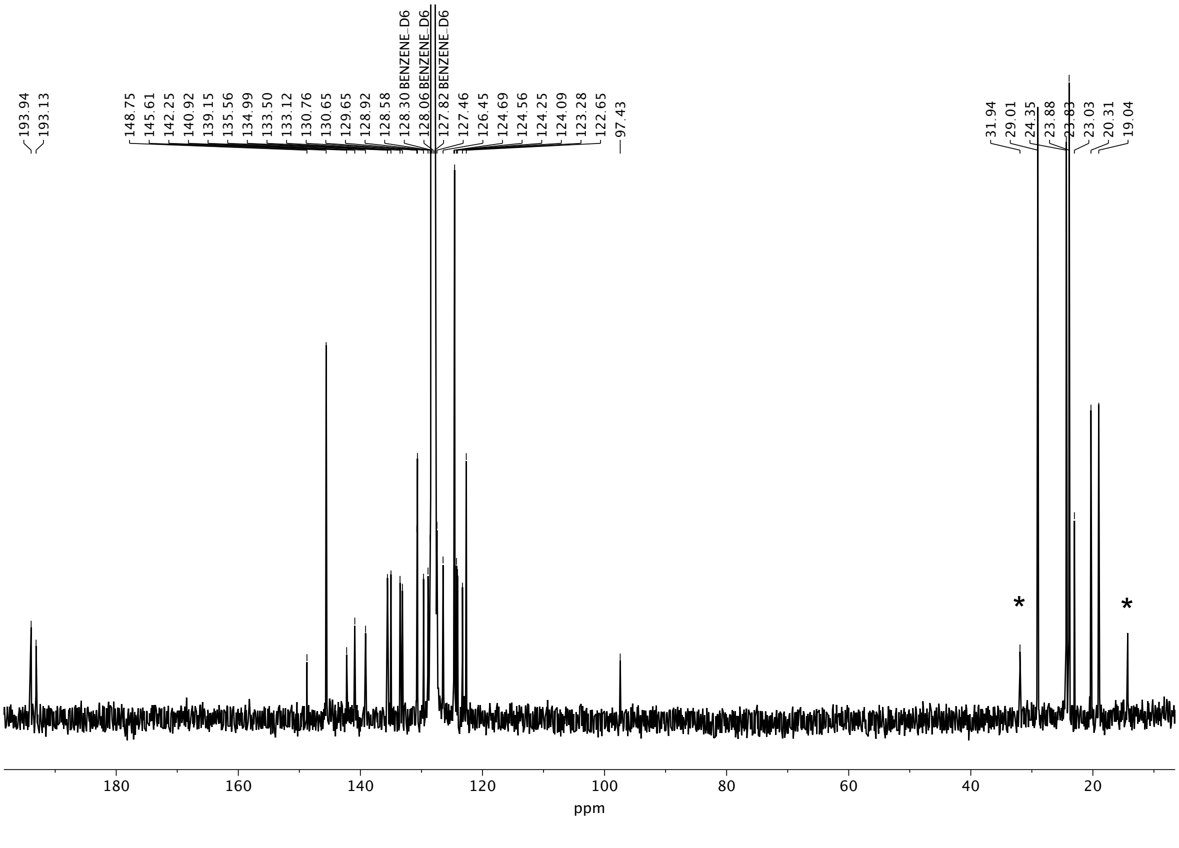

Figure S20. The ^{13}C NMR spectrum of **6** (*: hexane).

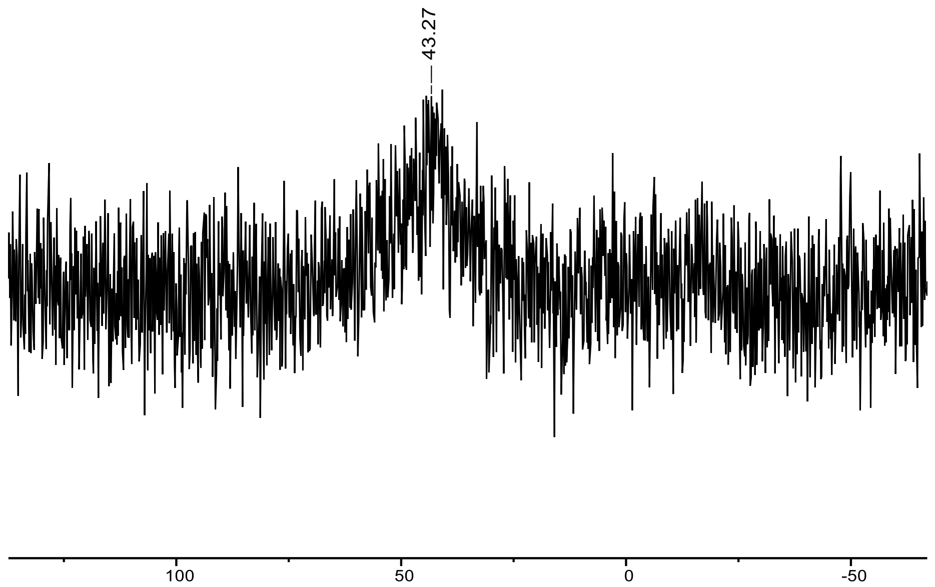

Figure S21. The ^{11}B NMR spectrum of **6**.

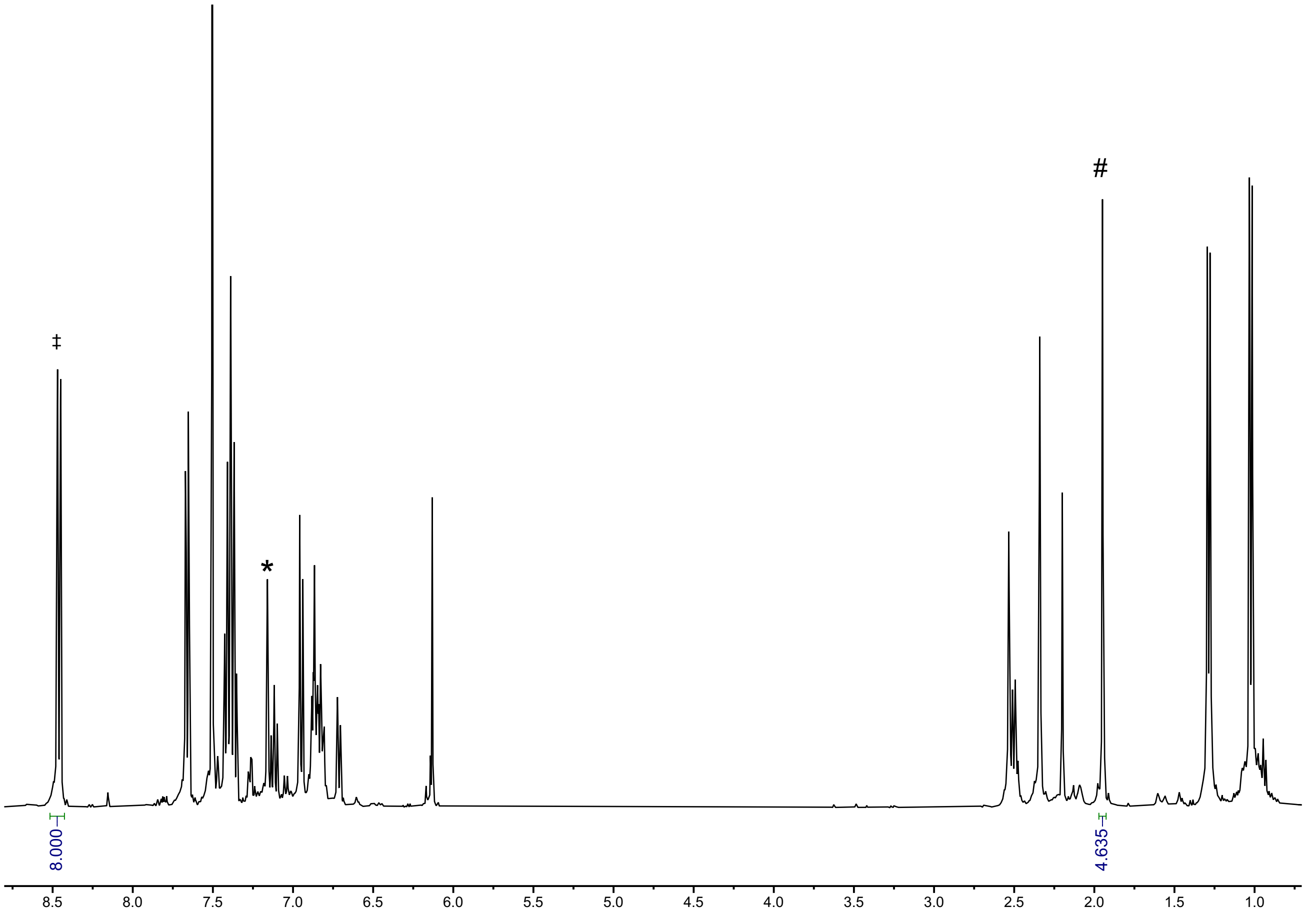

Figure S22. The ^{1}H NMR spectrum of the crude reaction mixture for the reaction of **2** with 2 eq. XylNC to estimate NMR yield [*: C_6D_5H, #: **6** (77% yield), ‡: phenanthrene].

Synthesis of 6-Mes

In a glovebox, a J young NMR tube was charged with **4** (18.6 mg, 20.5 μmol) and C_6D_6 (300 μL). To the resulting solution, a solution of Mes-NC (3.0 mg, 21 μmol) in C_6D_6 was added dropwise at room temperature. After heating the solution at 65 °C for 2.5 h, ^{1}H NMR spectrum of the crude reaction mixture was measured to check the reaction proceeded. Then the resulting reaction mixture was concentrated under reduced pressure and the crude product was recrystallized by a slow evaporation method with benzene to give **6-Mes** as green crystals (14.9 mg, 14.1 μmol, 69%), which are suitable for X-ray crystallographic analysis. However, it was difficult to obtain a sufficient amount of the analytically pure crystals of **6-Mes**, therefore, NMR spectra with a small amount of impurities are shown in Figures S23-S25.

^{1}H NMR (399 MHz, C_6D_6) δ 7.50 (d, *J* = 7 Hz, 1H), 7.29-7.23 (m, 1H), 7.21-7.10 (m, 3H), 6.98 (d, *J* = 8 Hz, 4H), 6.91-6.78 (m, 8H), 6.71 (d, *J* = 7 Hz, 2H), 6.67 (s, 2H), 6.15 (s, 2H), 2.53 (sept, *J* = 7 Hz, 4H), 2.53 (s, 3H) 2.34 (s, 6H), 2.22 (s, 3H), 2.19 (s, 3H), 1.93 (s, 6H), 1.30 (d, *J* = 7 Hz, 12H), 1.04 (d, *J* = 7 Hz, 12H).

^{13}C NMR (100 MHz, Toluene-*d*$_8$, -30 °C) δ 19.01 (CH_3), 20.32 (CH_3), 20.97 (CH_3), 23.07 (CH_3), 23.84(CH_3 of Dip), 23.88 (CH_3), 24.40 (CH_3 of Dip), 28.97 (CH of Dip), 97.31 (4°, C=*C*=N), 122.55 (CH), 122.92 (4°), 124.12 (CH), 124.28 (CH), 124.52 (CH), 124.64 (CH), 126.27 (CH), 127.47 (4°), 127.84 (CH), 128.74 (CH), 128.89 (CH), 129.67 (CH), 130.50 (4°), 130.62 (CH), 131.81 (CH), 130.87 (4°), 133.04 (CH), 133.47 (CH), 134.93 (4°), 135.51 (4°), 139.09 (4°), 139.79 (4°), 140.88 (4°), 145.52 (4°), 148.74 (4°), 193.20 (4°, Au–*C*=C). 193.79 (4°, carbene).

^{11}B NMR (128 MHz, C_6H_6) δ 43 (br)

HRMS (APCI/TOF) m/z: $[M+H]^+$ Calcd for $C_{60}H_{70}AuBN_4$ 1054.5364; Found 1054.5380.

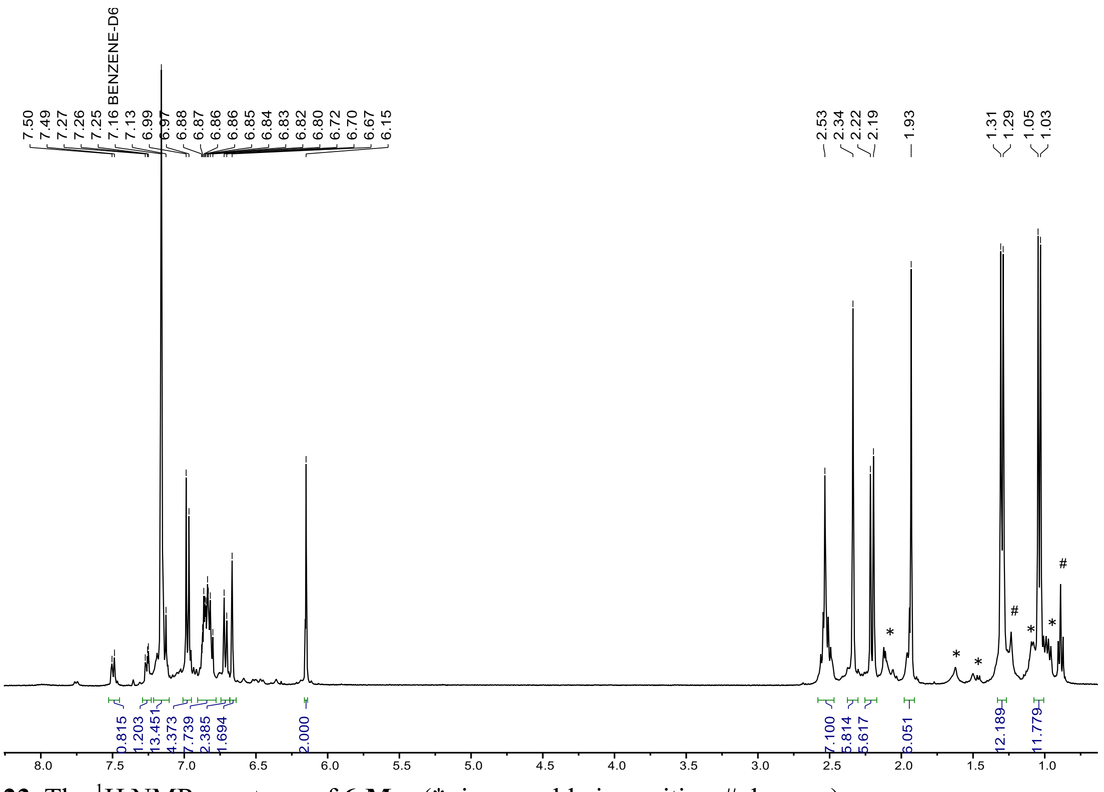

Figure S23. The ^{1}H NMR spectrum of **6-Mes** (*: inseparable impurities, #: hexane).

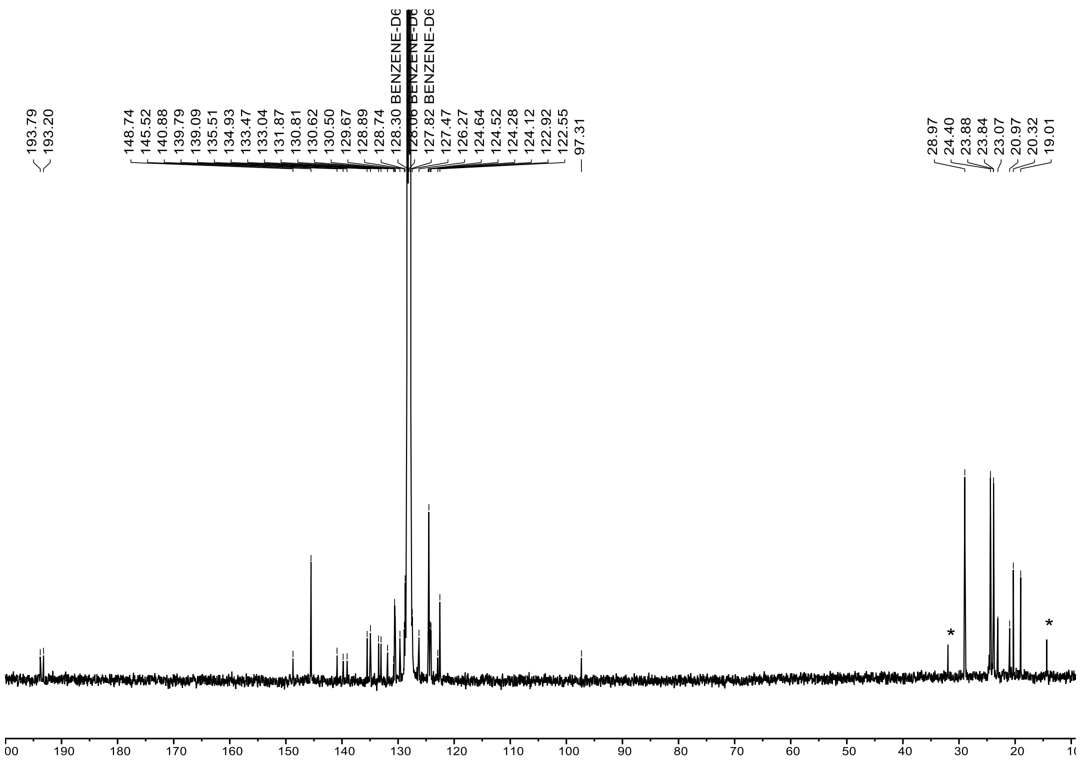

Figure S24. The ^{13}C NMR spectrum of **6-Mes**

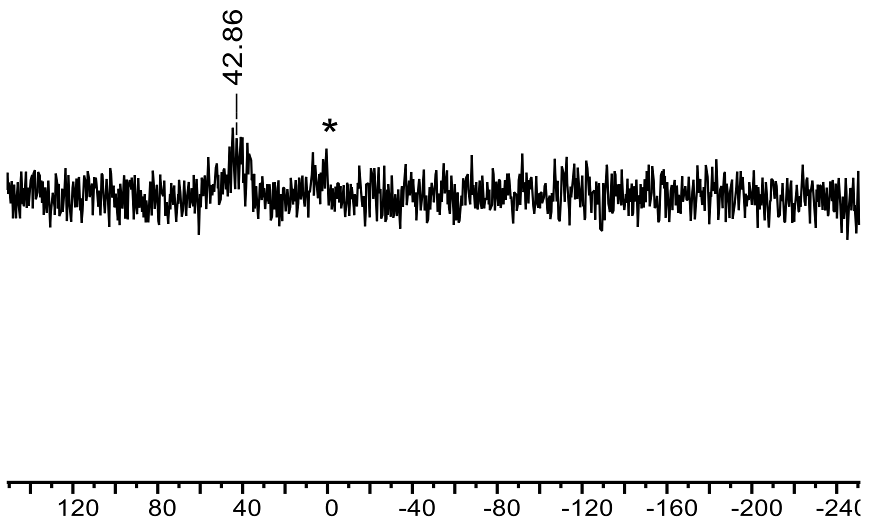

Figure S25. The ^{11}B NMR spectrum of **6-Mes** (*: byproduct)

Synthesis of 7, [IPrAuCHPh(OB(*o*-tol)$_2$)]

In a glovebox, benzene (3 mL) was added to a mixture of **2** (60.1 mg, 77.2 µmol) and benzaldehyde (8.2 mg, 77 µmol). After stirring the solution at room temperature for 1 min, the resulting reaction mixture was concentrated under reduced pressure. The crude product was reprecipitated with hexane and diethyl ether to give **7** as white powder (36.8 mg, 54%).

^{1}H NMR (500 MHz, Toluene-d_8, –30 °C) δ 7.59 (d, J = 6 Hz, 1H), 7.30-7.10 (m, 9H), 7.08-7.02 (m, 4H), 7.01-6.96 (m, 2H), 6.93 (d, J = 6 Hz, 3H), 6.85 (d, J = 6 Hz, 2H), 6.05 (s, 2H, vinyl-H), 5.78 (br s, 1H, CH), 2.85 (s, 3H, CH_3 of o-tol), 2.38 (sept, J = 7 Hz, 4H, CH of Dip), 2.11 (s, 3H, CH_3 of o-tol), 1.29 (d, J = 7 Hz, 6H, CH_3 of Dip), 1.28 (d, J = 7 Hz, 6H, CH_3 of Dip), 1.05 (d, J = 7 Hz, 6H, CH_3 of Dip), 1.03 (d, J = 7 Hz, 6H, CH_3 of Dip).

^{13}C NMR (100 MHz, Toluene-d_8, –30 °C) δ 22.35 (CH_3 of o-tolyl), 23.51 (CH_3 of Dip), 24.51 (CH_3 of o-tolyl), 24.85 (CH_3 of Dip), 24.94 (CH_3 of Dip), 28.75 (CH of Dip), 92.44 (br, CH–O), 121.67 (CH), 121.98 (CH), 123.86 (CH), 123.89 (CH), 123.92 (CH), 124.96 (CH), 125.36 (CH), 127.10 (CH), 127.48 (CH), 128.20 (CH), 129.12 (CH), 129.89 (CH), 130.18 (CH), 130.30 (CH), 130.36 (CH), 134.49 (4°), 138.73 (4°), 138.94 (CH), 139.10 (4°), 142.43 (br, 4°), 144.96 (4°), 145.29 (4°), 145.32 (4°), 153.12 (4°), 194.76 (4°, carbene).

^{11}B NMR (128 MHz, C_6H_6) δ 45 (br)

mp: 100-102 °C (decomp.)

Anal. Calc for $C_{48}H_{56}AuBN_2O$: C, 65.16; H, 6.38; N, 3.17. Found: C, 65.16; H, 6.33; N, 3.13.

Procedure for estimation of NMR yield

In a glovebox, a solution of **2** (10.9 mg, 14.0 μmol), benzaldehyde (149 μL, 94.2 mM, 14.0 μmol), and phenanthrene (10.0 mg, 56.1 μmol) in C_6D_6 (600 μL) was stirred at room temperature for 1 min. The ^{1}H NMR spectrum measurement of the crude reaction mixture was performed to estimate NMR yield (Figure S26, **7**: 92% yield).

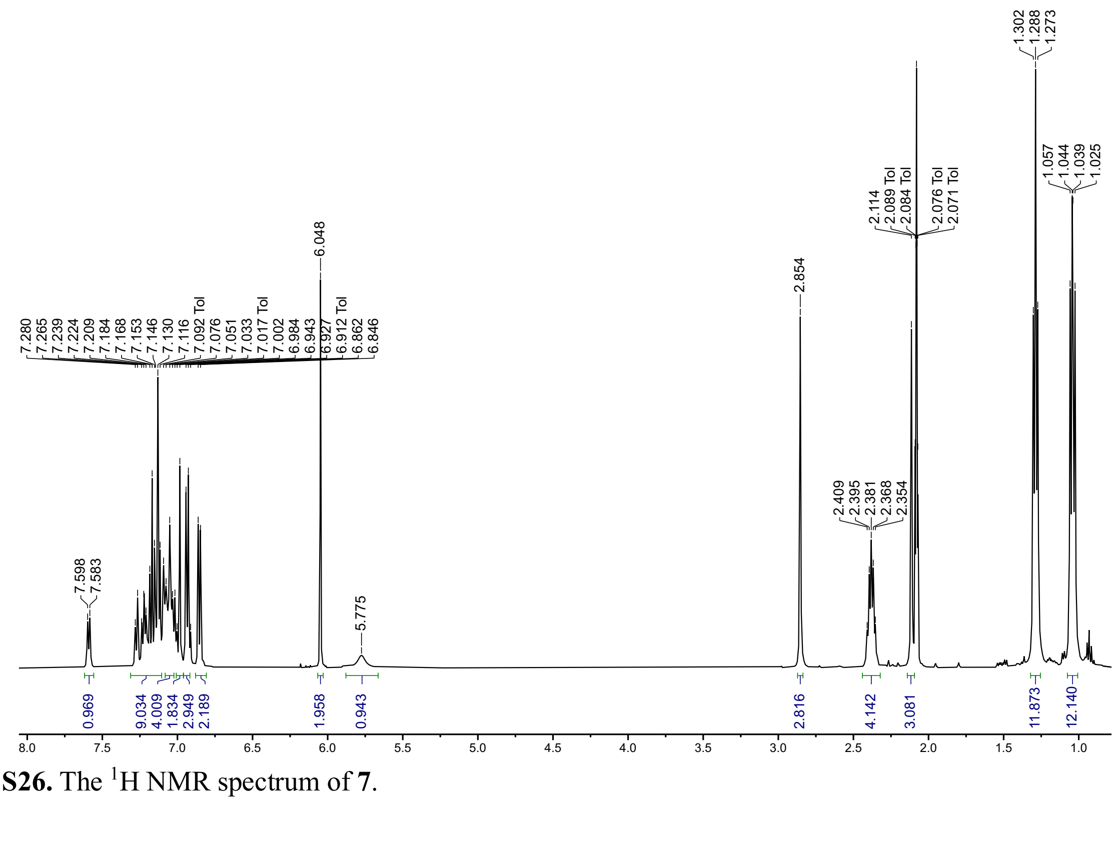

Figure S26. The ^{1}H NMR spectrum of **7**.

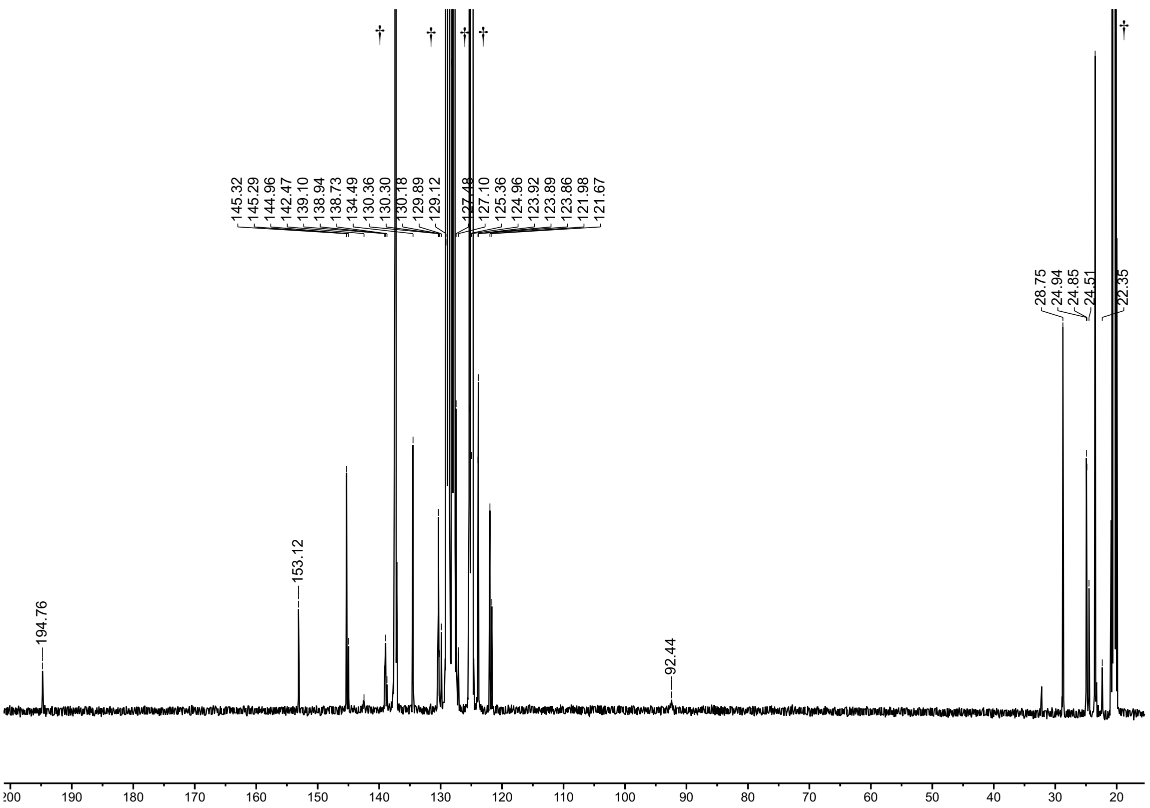

Figure S27. The ^{13}C NMR spectrum of **7** (†: toluene-d_8).

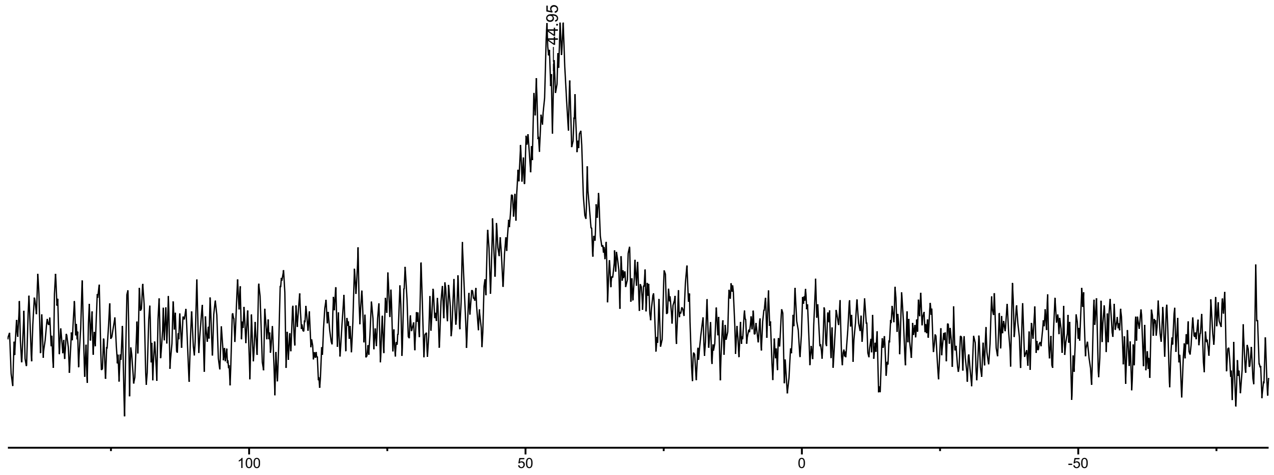

Figure S28. The ^{11}B NMR spectrum of **7**.

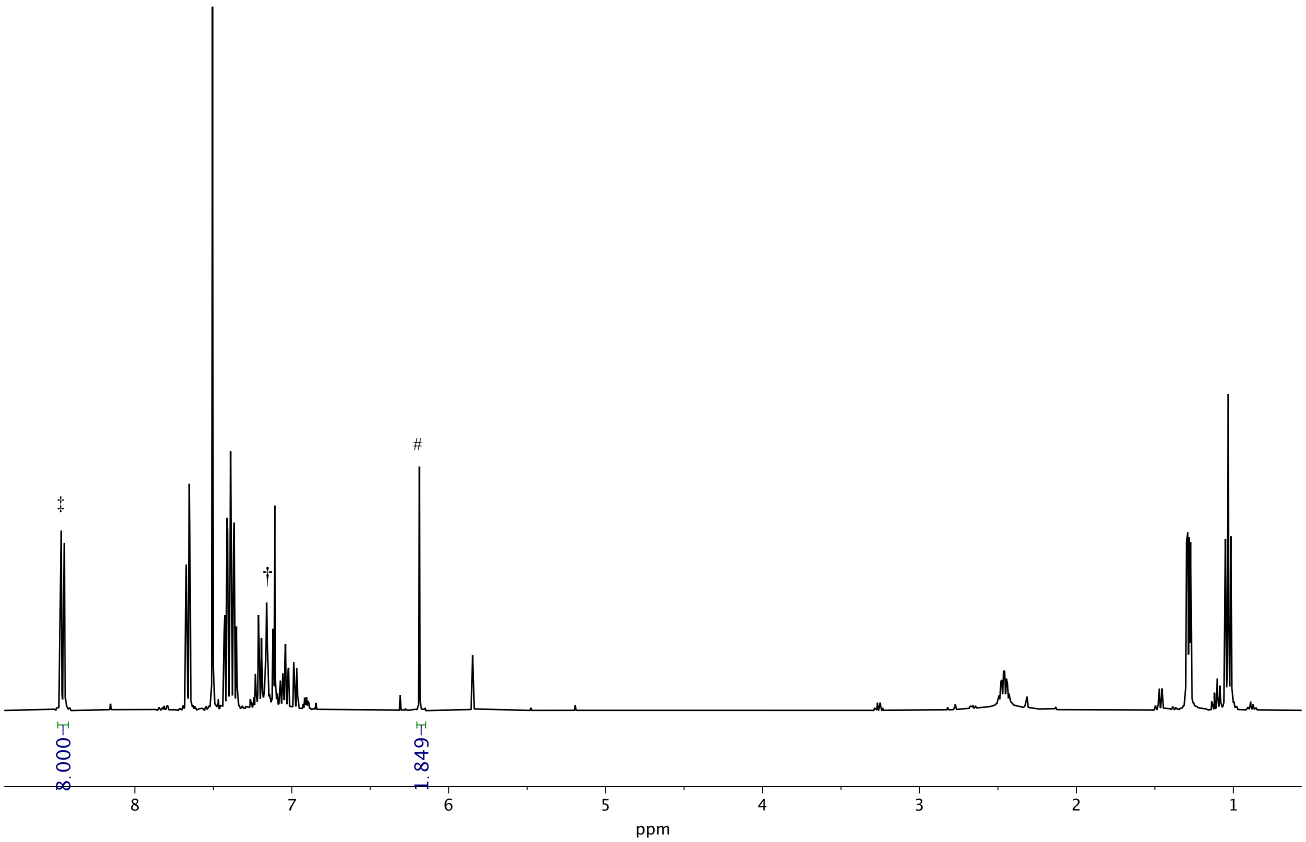

Figure S29. The ^{1}H NMR spectrum of the crude reaction mixture for the reaction of **2** with benzaldehyde to estimate NMR yield [†: C_6D_5H, #: **7** (92% yield), ‡: phenanthrene].

Synthesis of 8, [IPrAuCPh$_2$(OB(*o*-tol)$_2$)]

In a glovebox, benzene (1 mL) was added to a mixture of **2** (29.9 mg, 38.4 μmol) and benzophenone (7.0 mg, 38 μmol). After stirring the solution at room temperature for 1 min, the resulting reaction mixture was concentrated under reduced pressure and the crude product was recrystallized by a vapor diffusion method using diethyl ether/hexane to give **8** as colorless crystals (26.3 mg, 71%).

^{1}H NMR (399 MHz, C_6D_6) δ 7.43 (dd, J = 8, 1 Hz, 2H), 7.27-7.20 (m, 6H), 7.14-7.11 (m, 2H), 7.08-7.02 (m, 4H), 7.00-6.90 (m, 8H), 6.84 (tt, J = 8, 1 Hz, 2H), 6.24 (s, 2H, vinyl-H), 2.56 (sept, J = 7 Hz, 4H, CH of Dip), 2.19 (s, 6H, CH_3 of *o*-tol), 1.29 (d, J = 7 Hz, 12H, CH_3 of Dip), 1.03 (d, J = 7 Hz, 12H, CH_3 of Dip).

^{13}C NMR (100 MHz, C_6H_6) δ 23.13 (CH_3 of *o*-tolyl), 24.03 (CH_3 of Dip), 24.26 (CH_3 of Dip), 28.98 (CH of Dip), 105.39 (4°, C-O), 122.51 (CH, vinyl), 123.59 (CH), 124.38 (CH), 124.50 (CH), 127.35 (CH), 12.24 (CH), 129.63 (CH), 130.70 (CH), 133.39 (CH), 135.17 (4°), 140.88 (4°), 143.50 (4°), 145.52 (4°), 154.48 (4°), 193.80 (4°, carbene).

^{11}B NMR (128 MHz, C_6H_6) δ 45 (br)

mp: 120-125°C (decomp.)

Anal. Calc for $C_{54}H_{60}AuBN_2O$: C, 67.50; H, 6.29; N, 2.92. Found: C, 67.35; H, 6.21; N, 2.91.

Procedure for estimation of NMR yield

In a glovebox, a solution of **2** (10.9 mg, 14.0 μmol), pyridine (256 μL, 54.9 mM, 14.0 μmol), and phenanthrene (10.0

mg, 56.1 μmol) in C_6D_6 (600 μL) was stirred at room temperature for 1 min. The ^{1}H NMR spectrum measurement of the crude reaction mixture was performed to estimate NMR yield (Figure S30, **8**: 85% yield).

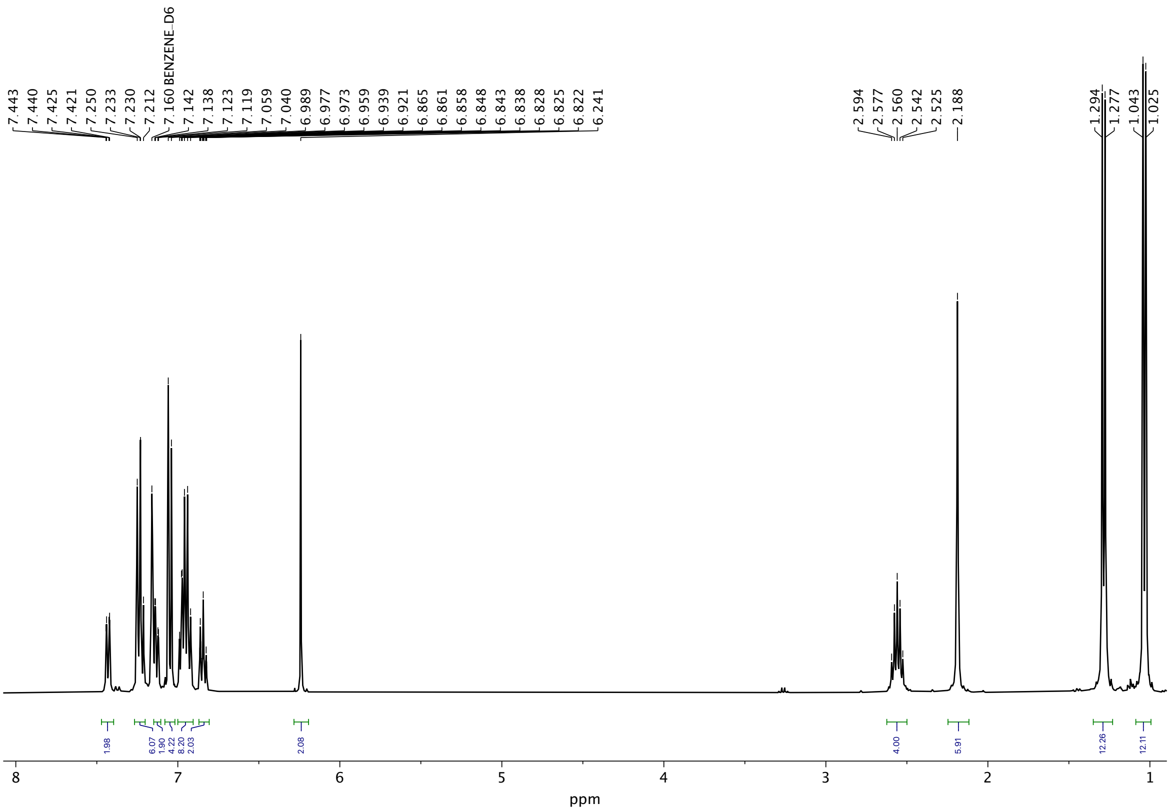

Figure S30. The ^{1}H NMR spectrum of **8**.

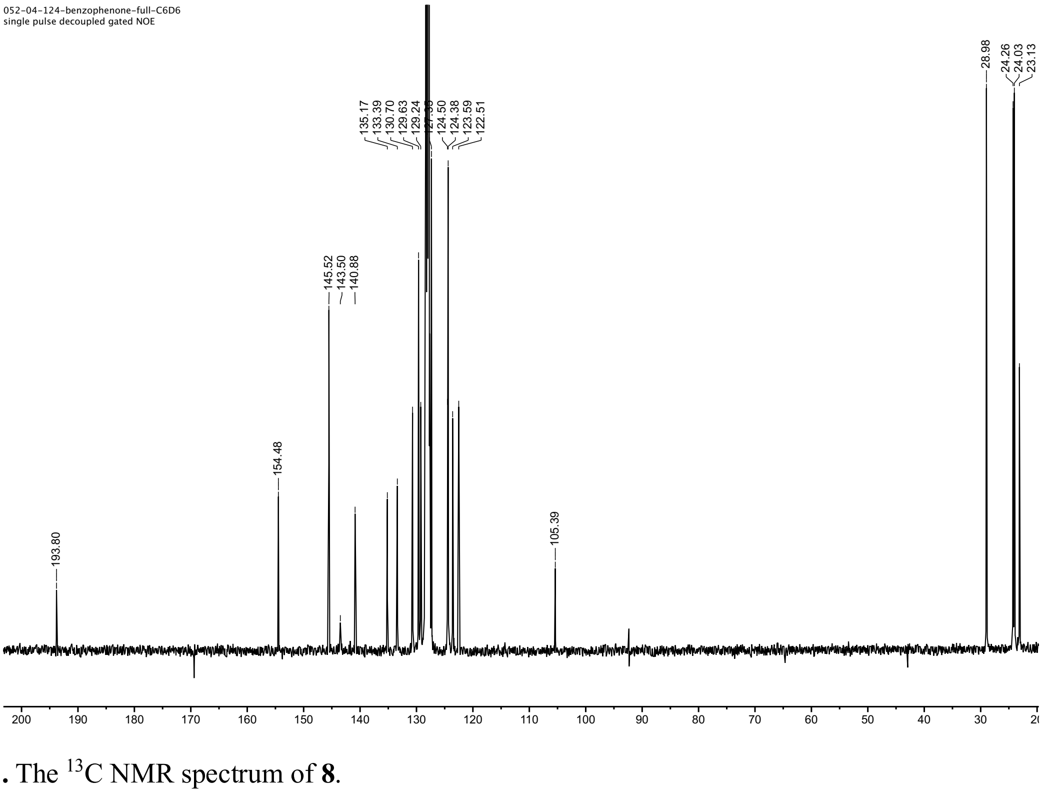

Figure S31. The ^{13}C NMR spectrum of **8**.

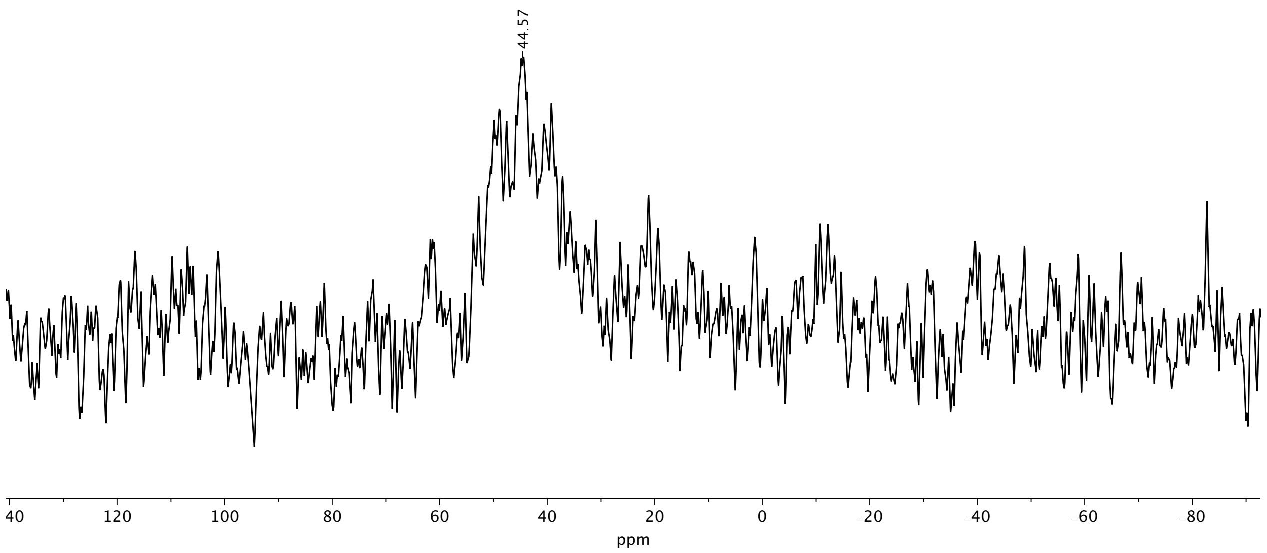

Figure S32. The ^{11}B NMR spectrum of **8**.

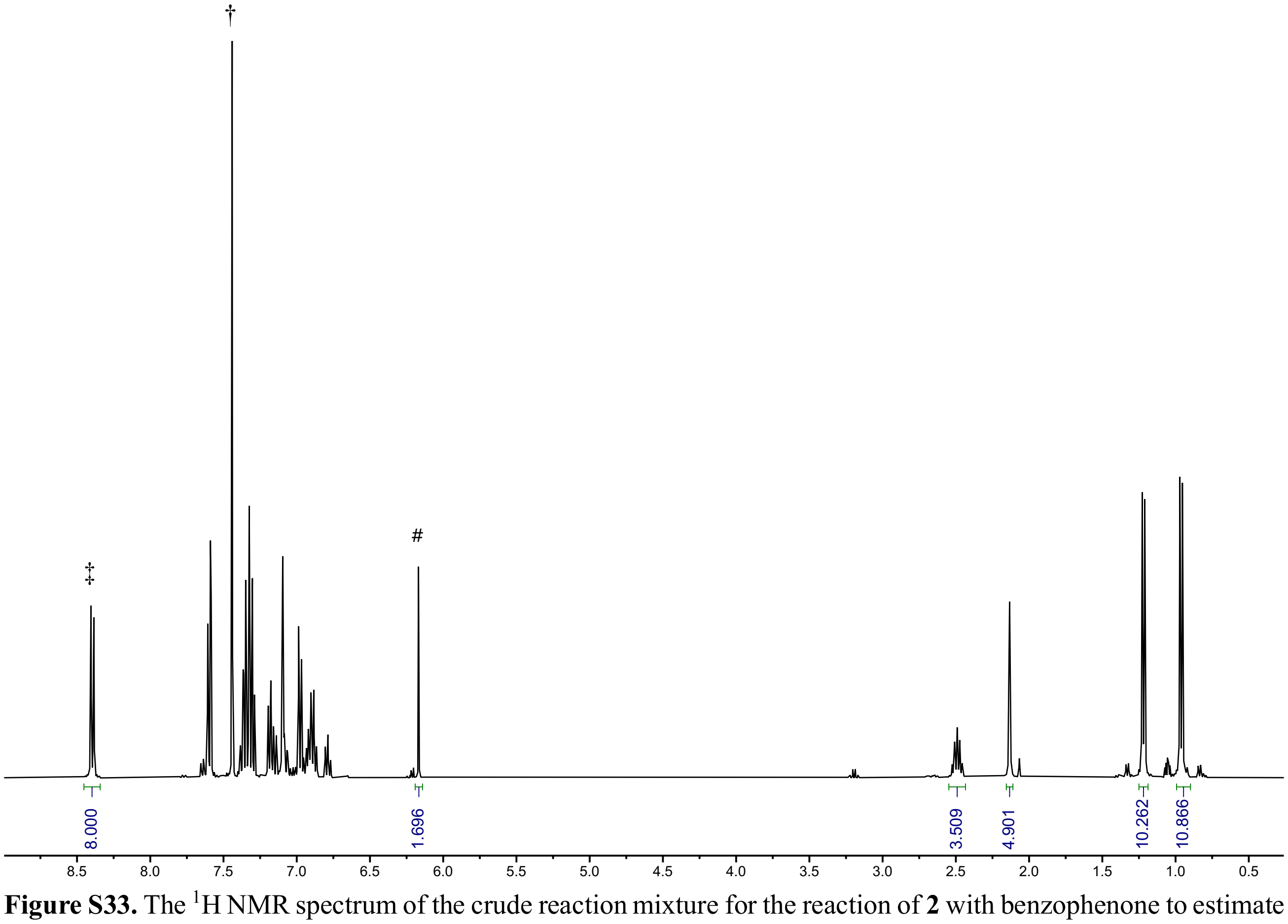

Figure S33. The ^{1}H NMR spectrum of the crude reaction mixture for the reaction of **2** with benzophenone to estimate NMR yield [†: C_6D_5H, #: **8** (85% yield), ‡: phenanthrene].

Synthesis of 9, [IPrAuC(=O$^+$–B$^-$(*o*-tol)$_2$Cl)(4-FC$_6$H$_4$)]

In a glovebox, toluene (1 mL) was added to a mixture of **2** (27.3 mg, 35.1 μmol) and 4-fluorobenzoyl chloride (5.6 mg, 35 μmol) at room temperature. After stirring the solution at room temperature for 1 min, the resulting reaction

mixture was recrystallized by vapor diffusion method using toluene/pentane to give **9** as yellow crystals (23.9 mg, 73%).

^{1}H NMR (399 MHz, C_6D_6) δ 7.84 (d, *J* = 7 Hz, 2H), 7.54 (dd, *J* = 9, 6 Hz, 2H), 7.31 (t, *J* = 8 Hz, 2H), 7.26-7.16 (m, 6H), 7.13 (d, *J* = 8 Hz, 2H), 6.56 (t, *J* = 9 Hz, 2H), 6.31 (s, 2H, vinyl-H), 2.47 (sept, *J* = 7 Hz, 4H, CH of Dip), 2.45 (s, 6H, CH_3 of *o*-tol), 1.21 (d, *J* = 7 Hz, 12H, CH_3 of Dip), 1.03 (d, *J* = 7 Hz, 12H, CH_3 of Dip).

^{13}C NMR (100 MHz, C_6H_6) δ 23.72 (CH_3 of *o*-tolyl), 24.17 (CH_3 of Dip), 24.37 (CH_3 of Dip), 29.08 (CH of Dip), 115.35 (CH), 115.57 (CH), 123.39 (CH), 124.62 (CH), 124.75(CH), 126.29 (CH), 128.35 (CH), 130.39 (CH), 130.90 (CH), 133.52 (CH), 134.87 (4°), 136.45 (CH), 136.55 (CH), 141.68 (4°), 143.81 (4°), 145.76 (4°), 148.62 (4°, C=O), 166.57 (4°, d, *J* = 256 Hz, CF), 190.85 (4°, carbene)

^{11}B NMR (128 MHz, C_6H_6) δ 11 (br)

^{19}F NMR (MHz, C_6H_6) δ 103.75

mp: 128-130 °C (decomp.)

Anal. Calc for $C_{48}H_{56}AuBClFN_2O$: C, 61.52; H, 5.81; N, 2.99. Found: C, 61.43; H, 5.94; N, 3.02.

Procedure for estimation of NMR yield

In a glovebox, a solution of **2** (10.9 mg, 14.0 μmol), 4-fluorobenzoyl chloride (222 μL, 63.1 mM, 14.0 μmol), and phenanthrene (10.0 mg, 56.1 μmol) in C_6D_6 (600 μL) was stirred at room temperature for 1 min. The ^{1}H NMR spectrum measurement of the crude reaction mixture was performed to estimate NMR yield (Figure S35, **9**: 82% yield).

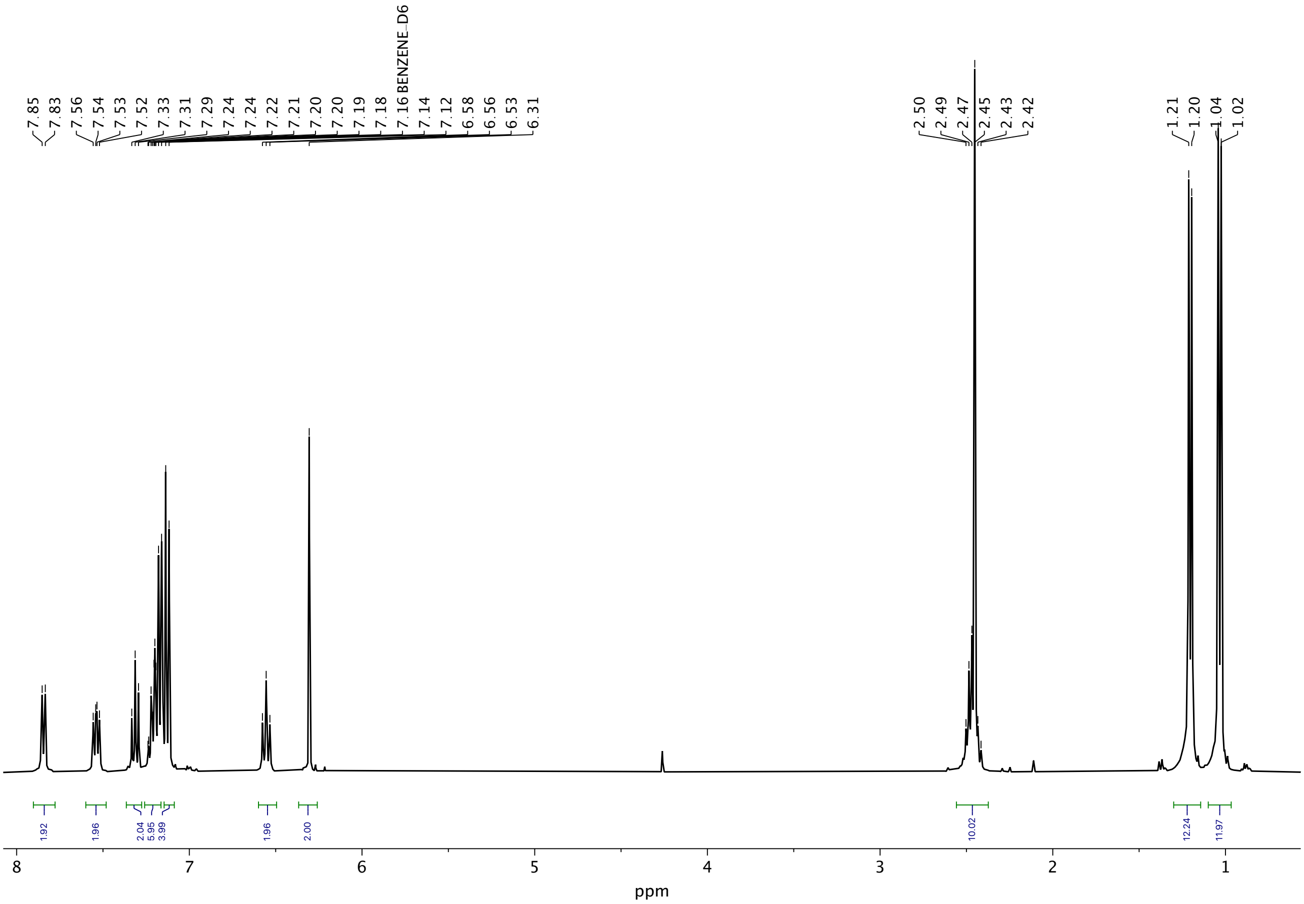

Figure S34. The ^{1}H NMR spectrum of **9**.

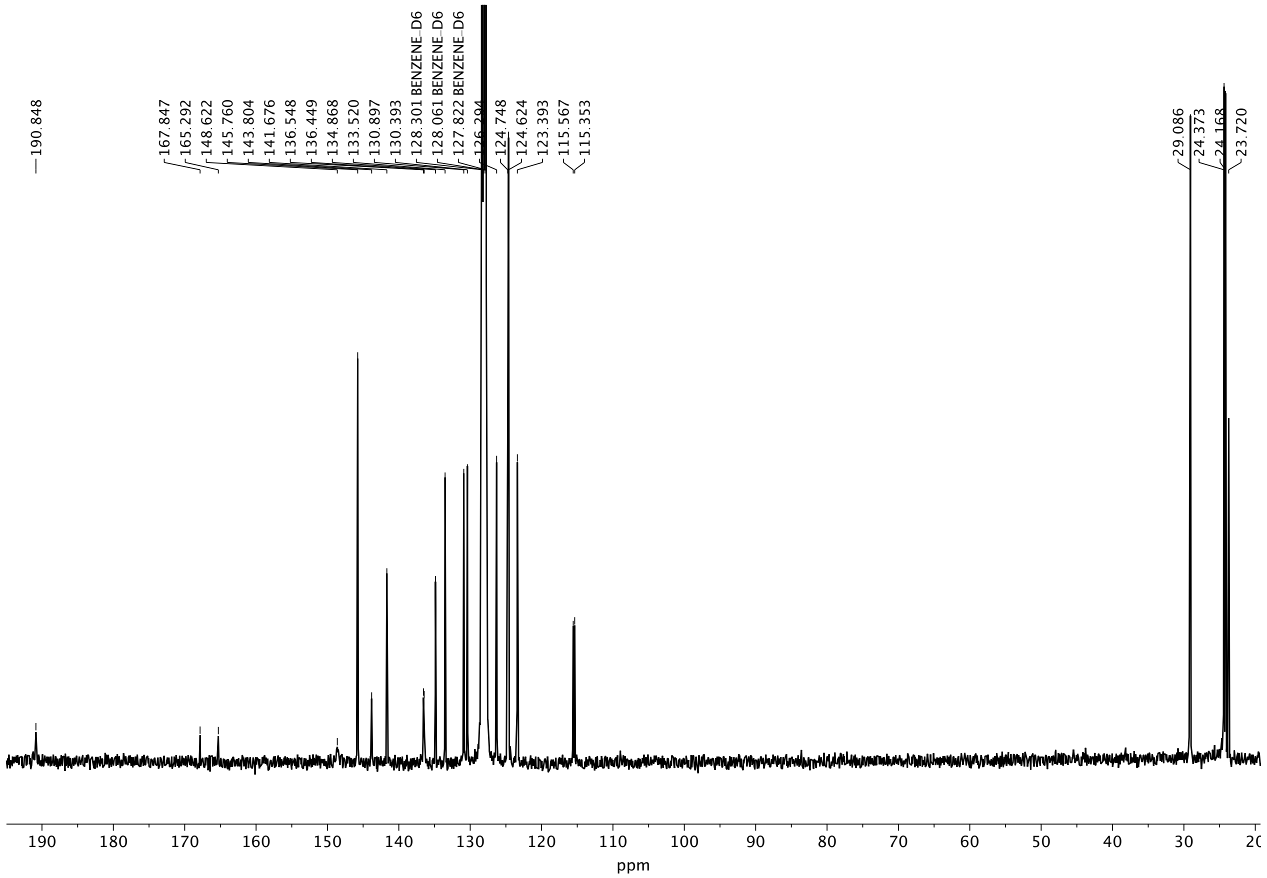

Figure S35. The ^{13}C NMR spectrum of **9**.

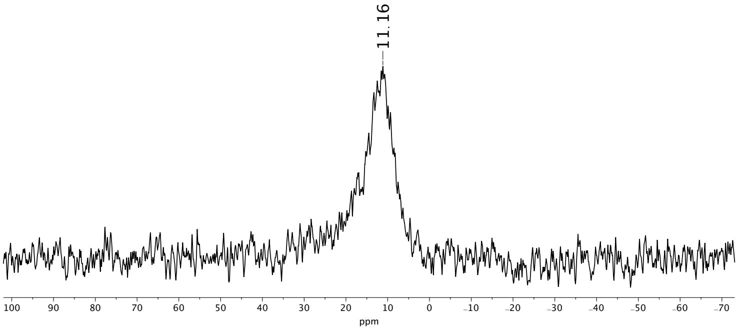

Figure S36. The ^{11}B NMR spectrum of **9**.

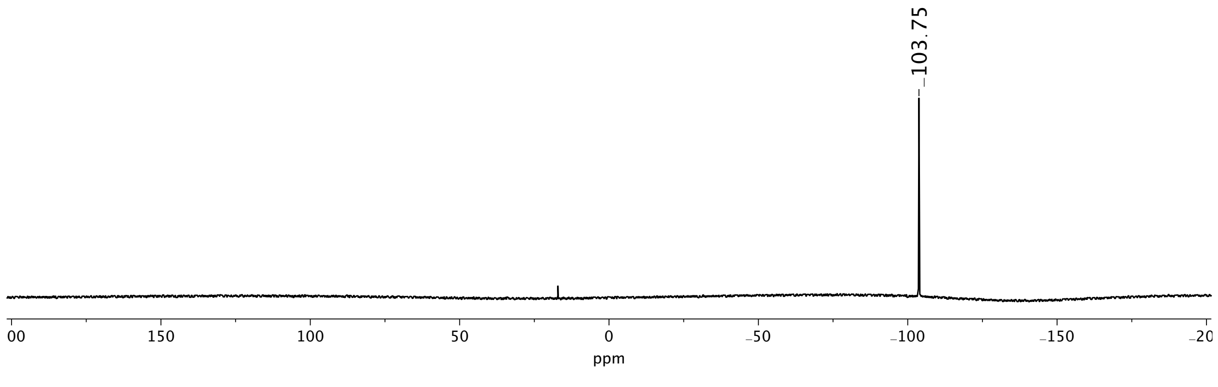

Figure S37. The ^{119}F NMR spectrum of **9**.

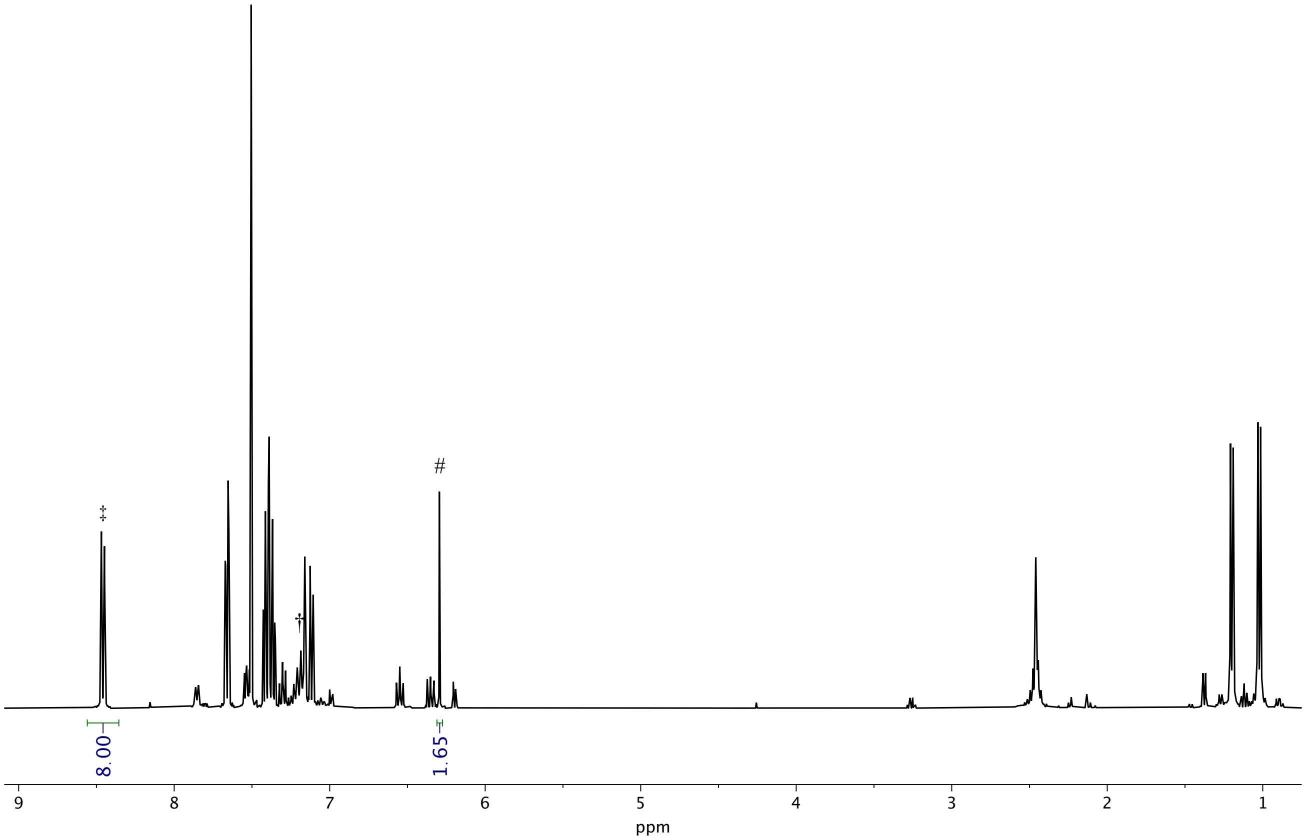

Figure S38. The ^{1}H NMR spectrum of the crude reaction mixture for the reaction of **2** with 4-fluorobenzoyl chloride [†: C_6D_5H, #: **9** (82% yield), ‡: phenanthrene].

Synthesis of 10, [IPrAuC(NCy)$_2$B(*o*-tol)$_2$]

In a glovebox, benzene (1 mL) was added to a mixture of **2** (37.7 mg, 48.4 μmol) and *N,N'*-dicyclohexylcarbodiimide (10.0 mg, 48.4 μmol) at room temperature. After stirring the solution at room temperature for 1 min, the resulting reaction mixture was concentrated and recrystallized by vapor diffusion method using diethyl ether/hexane to give **10** as colorless crystals (26.0 mg, 55%).

^{1}H NMR (399 MHz, C_6D_6) δ 8.08 (dd, *J* = 7, 1 Hz, 2H), 7.27-7.16 (m, 8H), 7.02 (d, *J* = 7 Hz, 4H), 6.21 (s, 2H, vinyl-H), 3.11 (tt, *J* = 12, 4 Hz, 2H), 2.51 (sept, *J* = 7 Hz, 4H, CH of Dip), 2.43 (s, 6H, CH_3 of *o*-tol), 1.71 (d, *J* = 11 Hz, 4H), 1.47 (t, J = 15 Hz, 6H), 1.36 (d, *J* = 7 Hz, 12H, CH_3 of Dip), 1.29 (t, *J* = 12 Hz, 4H), 1.04 (d, *J* = 7 Hz, 12H, CH_3 of Dip), 1.01-0.79 (m 6H).

^{13}C NMR (100 MHz, C_6H_6) δ 23.80 (CH_3 of *o*-tolyl), 24.16 (CH_3 of Dip), 24.62 (CH_3 of Dip), 26.21 (CH_2 of Cy), 26.78 (CH_2 of Cy), 28.99 (CH of Dip), 35.85 (CH_2 of Cy), 58.55 (CH of Cy), 123.11 (CH, vinyl), 124.41 (CH), 125.28 (CH), 125.28 (CH), 125.61 (CH), 128.35 (CH), 129.60 (CH), 130.99 (CH), 133.49 (CH), 134.53 (4°), 141.27 (4°), 145.70 (4°), 151.53 (br, 4°), 195.37 (4°, carbene), 209.77 (4°, C of NCN)

^{11}B NMR (128 MHz, C_6H_6) δ 9 (br)

mp: 185-188 °C (decomp.)

Anal. Calc for $C_{54}H_{72}AuBN_4$: C, 65.85; H, 7.37; N, 5.69. Found: C, 65.82; H, 7.23; N, 5.65.

Procedure for estimation of NMR yield

In a glovebox, a solution of **2** (10.9 mg, 14.0 μmol), *N,N'*-dicyclohexylcarbodiimide (289 μL, 48.5 mM, 14.0 μmol), and phenanthrene (10.0 mg, 56.1 μmol) in C_6D_6 (600 μL) was stirred at room temperature for 1 min. The ^1H NMR spectrum measurement of the crude reaction mixture was performed to estimate NMR yield (Figure S39, **10**: 92% yield).

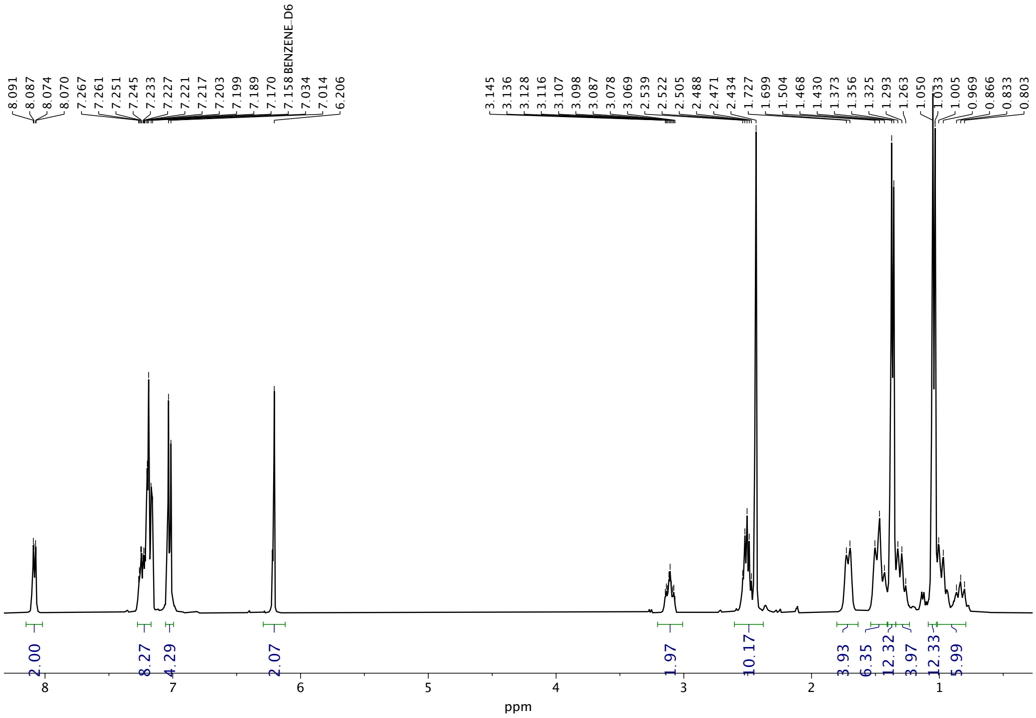

Figure S39. The ^1H NMR spectrum of **10**.

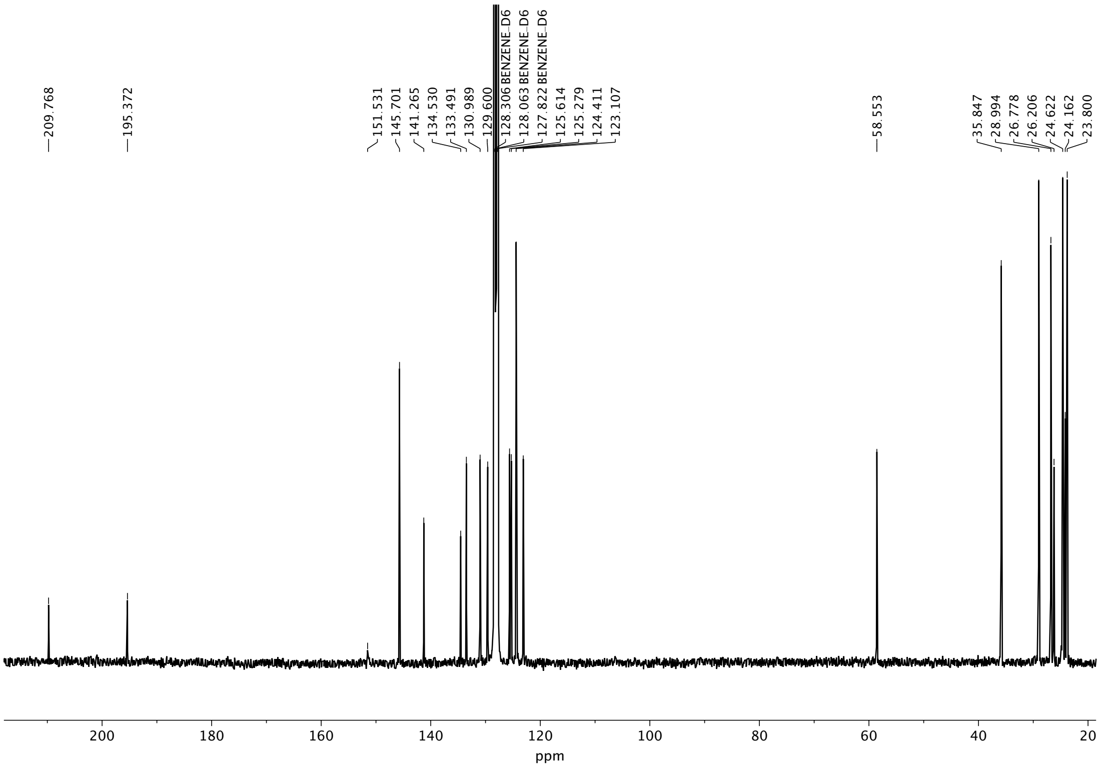

Figure S40. The ^{13}C NMR spectrum of **10**.

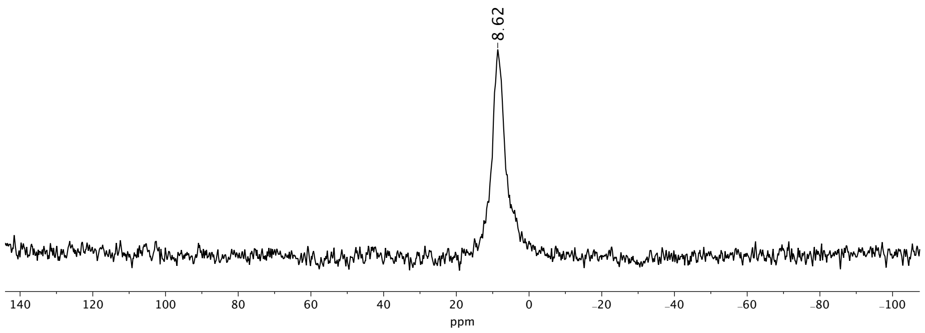

Figure S41. The ^{11}B NMR spectrum of **10**.

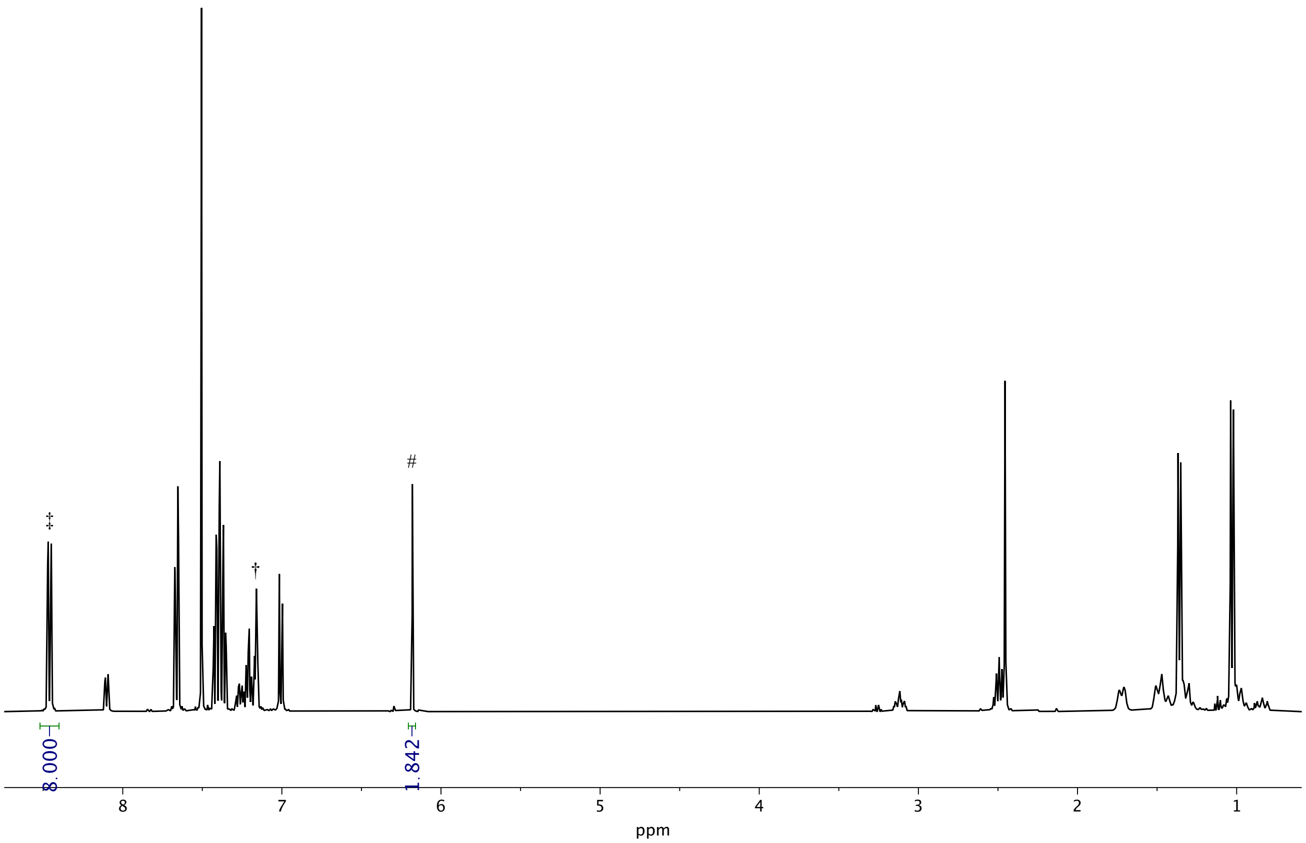

Figure S42. The ^{1}H NMR spectrum of the crude reaction mixture for the reaction of **2** with DCC [†: C_6D_5H, #: **10** (92% yield), ‡: phenanthrene].

Details for X-ray crystallography

Crystallographic data for **2**, **3a**, **3b**, **4**, **5**, **6**, **6-Mes**, **7**, **8**, **9**, and **10** are summarized in Table S1. The crystals were coated with immersion oil and put on a MicroMountTM (MiTeGen, LLC), and then mounted on diffractometer. Diffraction data were collected on a Rigaku XtaLAB Synergy diffractometer equipped with a HyPix-6000 hybrid pixel detector using MoKα radiation. The Bragg spots were integrated using CrysAlisPro program package.[2] Absorption corrections were applied. All the following procedure for analysis, Yadokari-XG 2009[3] was used as a graphical interface. The structure was solved by a direct method with programs of SHELXS[4] and SIR-2014[5] refined by a full-matrix least squares method with the program of SHELXL-2016 or SHELXL-2018.[4] Anisotropic temperature factors were applied to all non-hydrogen atoms. The hydrogen atoms were put at calculated positions, and refined applying riding models. The detailed crystallographic data have been deposited with the Cambridge Crystallographic Data Centre: Deposition code CCDC 2020132-2020142. A copy of the data can be obtained free of charge via the following URL.

http://www.ccdc.cam.ac.uk/products/csd/request.

Table S1.

Crystallographic data and structure refinement details for **2**, **3a**, **3b**, **4**, **5**, **6**, **6-Mes**, **7**, **8**, **9**, and **10**.

compound #	**2**	**3a**	**3b**	**4**
CCDC deposit #	2020132	2020133	2020134	2020135
Empirical formula	$C_{41}H_{50}AuBN_2$	$C_{46}H_{55}AuBN_3$	$C_{48}H_{60}AuBN_4$	$C_{50}H_{59}AuBN_3$
Formula weight	778.60	857.70	900.77	909.77
T (K)	93(2)	213(2)	93(2)	93(2)
λ (Å)	0.71073	0.71073	0.71073	0.71073
Crystal system	*Triclinic*	*Triclinic*	*Monoclinic*	*Triclinic*
Space group	*P*-1	*P*-1	*Cc*	*P*-1
a (Å)	10.5714(3)	10.7603(4)	14.0210(2)	12.30650(10)
b (Å)	18.1828(6)	11.6039(4)	17.7771(3)	17.9900(2)
c (Å)	20.5890(9)	19.3138(6)	17.7051(3)	20.3257(2)
α (°)	72.838(3)	72.527(3)	90	83.3400(10)
β (°)	89.216(3)	85.454(3)	97.0981(15)	79.7420(10)
γ (°)	74.717(3)	66.378(3)	90	83.0840(10)
V (Å^3)	3638.8(2)	2105.2	4379.22(12)	4375.21(8)
Z	4	2	4	4
$D_{calc,}$ (Mg/m^3)	1.421	1.353	1.366	1.381
μ (mm^{-1})	4.073	3.527	3.396	3.399
F(000)	1576	872	1840	1856
Crystal size (mm)	0.130×0.080×0.050	0.156×0.104×0.038	0.11×0.60×0.40	0.078×0.069×0.018
2θ range (°)	2.065-25.350	1.984-30.900	1.859-27.499	1.690-27.500
reflns collected	21641	33150	24141	70437
Indep reflns/R_{int}	21641/0.061	10697/0.0507	8573/0.0252	19794/0.0534
param	935	470	499	1015
GOF on F^2	1.054	0.968	1.043	1.001
R_1, wR_2 [I>2σ(I)]	0.0431, 0.1230	0.0329, 0.0614	0.0195, 0.0420	0.0317, 0.0559
R_1, wR_2 (all data)	0.0547, 0.1313	0.0510, 0.0651	0.0219, 0.0426	0.0520, 0.0595

compound #	**5**	**6**	**6-Mes**	**7**
CCDC deposit #	2020136	2020137	2020138	2020139
Empirical formula	$C_{63}H_{78}AuBN_4O$	$C_{59}H_{68}AuBN_4$	$C_{60}H_{70}AuBN_4$	$C_{54}H_{62}AuBN_2O$
Formula weight	1115.07	1040.95	1054.97	962.83
T (K)	93(2)	93(2)	93(2)	93(2)
λ (Å)	0.71073	0.71073	0.71073	0.71073
Crystal system	*Monoclinic*	*Triclinic*	*Triclinic*	*Triclinic*
Space group	$P2_1/n$	P-1	P-1	P-1
a (Å)	13.6039(2)	11.5634(3)	11.9510(3)	11.8822(4)
b (Å)	23.0648(3)	12.8016(4)	12.9306(3)	12.5800(2)
c (Å)	18.7378(3)	18.2287(5)	18.7852(6)	17.5818(5)
α (°)	90	86.266(2)	96.941(2)	99.538(2).
β (°)	105.489(2)	72.324(2)	104.913(3)	106.748(3)
γ (°)	90	83.521(2)	104.703(2)	103.924(2)
V (Å^3)	5665.85(15)	2553.22(13)	2658.94(13)	2363.27(12)
Z	4	2	2	2
$D_{calc,}$ (Mg/m^3)	1.307	1.354	1.318	1.353
μ (mm^{-1})	2.640	2.922	2.807	3.151
F(000)	2304	1068	1084	984
Crystal size (mm)	0.019×0.050×0.071	0.130×0.040×0.010	0.114×0.091×0.033	0.146×0.131×0.036
2θ range (°)	2.865-31.579	1.857-30.796	1.661-30.916	1.856-30.855
reflns collected	68817	40361	45273	36827
Indep reflns/R_{int}	16189/0.0433	12948/0.0453	13468/0.0756	11853/0.0468
param	647	600	685	542
GOF on F^2	1.014	1.022	0.988	1.057
R_1, wR_2 [I>2σ(I)]	0.0344, 0.0631	0.0287, 0.0561	0.0450, 0.0948	0.0336, 0.0827
R_1, wR_2 (all data)	0.0543, 0.0675	0.0382, 0.0580	0.0801, 0.1037	0.0412, 0.0857

compound #	**8**	**9**	**10**
CCDC deposit #	2020140	2020141	2020142
Empirical formula	$C_{54}H_{60}AuBN_2O$	$C_{48}H_{54}AuBClFN_2O$	$C_{54}H_{72}AuBN_4$
Formula weight	960.81	980.24	984.93
T (K)	93(2)	93(2)	93(2)
λ (Å)	0.71073	0.71073	0.71073
Crystal system	*Orthorhombic*	*Monoclinic*	*Monoclinic*
Space group	*Pbca*	$P2_1/n$	C_2/c
a (Å)	18.4407(4)	10.7485(2)	12.3058(4)
b (Å)	23.6412(5)	22.6769(4)	26.0004(6)
c (Å)	21.2380(5)	19.5737(4)	16.2840(3)
α (°)	90	90	90
β (°)	90	104.387(2)	100.267(2)
γ (°)	90	90	90
V (Å^3)	9258.9(4)	4621.33(16)	5126.7(2)
Z	8	4	4
$D_{calc,}$ (Mg/m^3)	1.379	1.409	1.276
μ (mm^{-1})	3.217	3.283	2.906
F(000)	3920	1996	2032
Crystal size (mm)	0.15×0.06×0.04	0.11×0.10×0.07	0.166×0.095×0.051
2θ range (°)	1.697-27.499	2.093-27.499	1.566-31.684
reflns collected	63633	43766	25286
Indep reflns/R_{int}	10630/0.0546	10604/0.0380	7173/0.0471
param	542	560	278
GOF on F^2	1.021	1.051	1.053
R_1, wR_2 [I>2σ(I)]	0.0303, 0.0530	0.0267, 0.0557	0.0411, 0.0857
R_1, wR_2 (all data)	0.0482, 0.0570	0.0345, 0.0577	0.0507, 0.0887

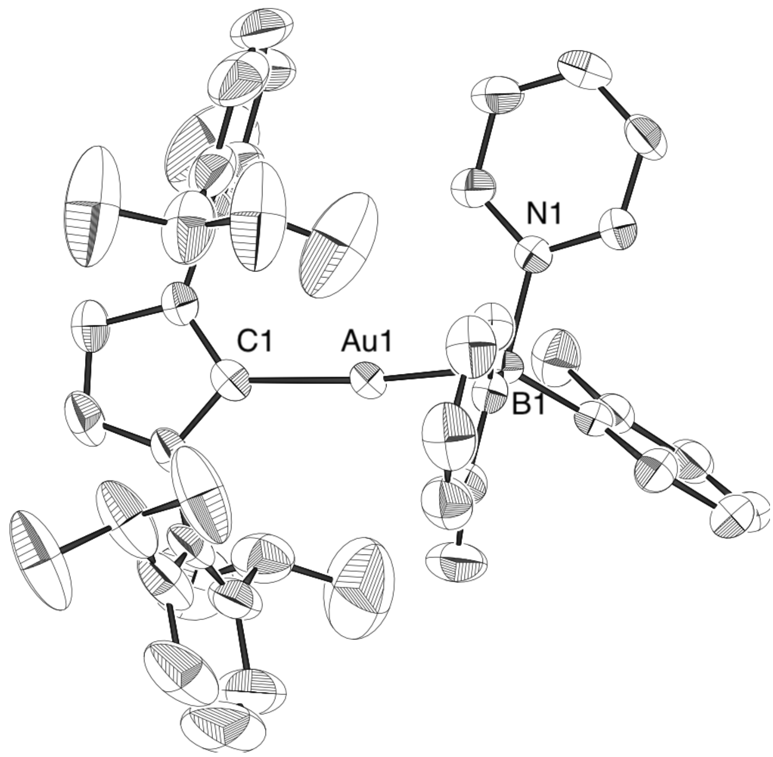

Figure S43. Molecular structures of **3a** with thermal ellipsoids at 50% probability (213 K); hydrogen atoms omitted for clarity.

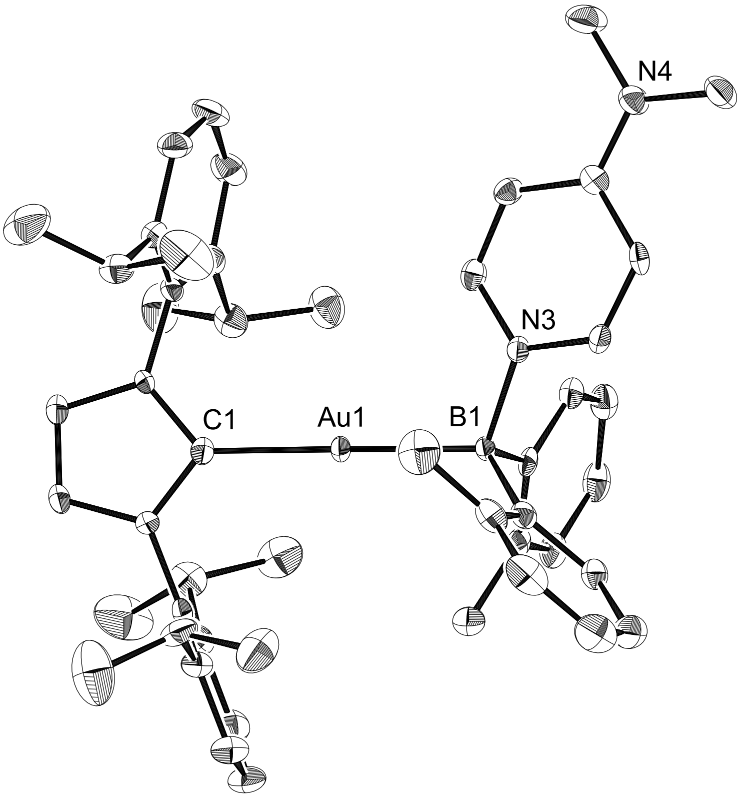

Figure S44. Molecular structures of **3b** with thermal ellipsoids at 50% probability (213 K); hydrogen atoms omitted for clarity.

3. **UV-Vis Absorption**

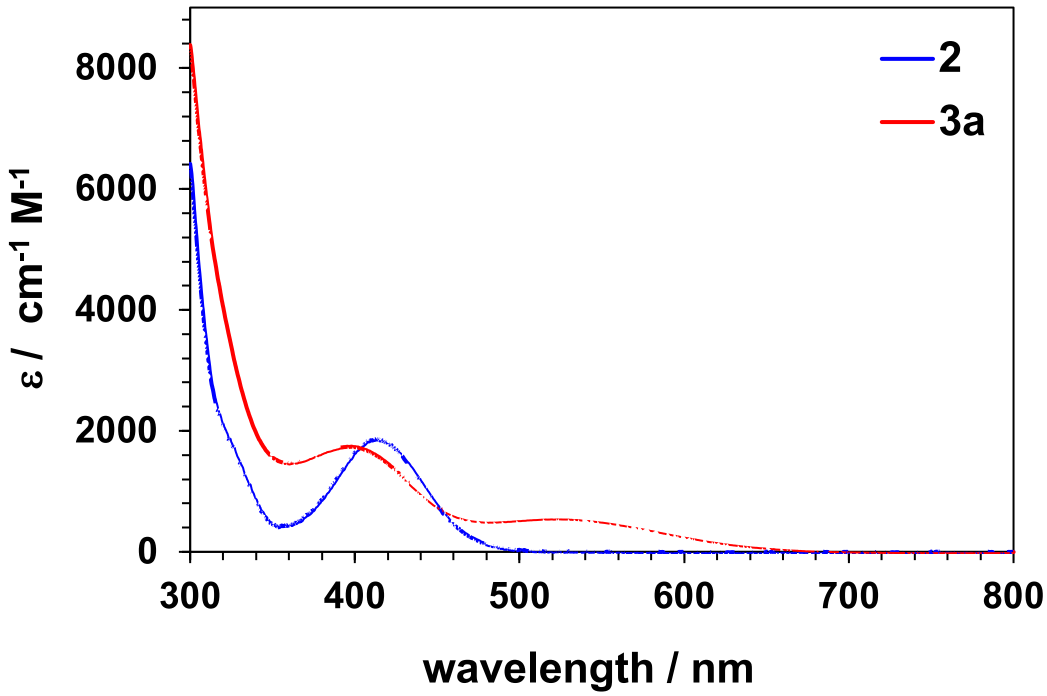

Figure S45. UV-Vis absorption spectra **2**, **3a** (toluene solution)

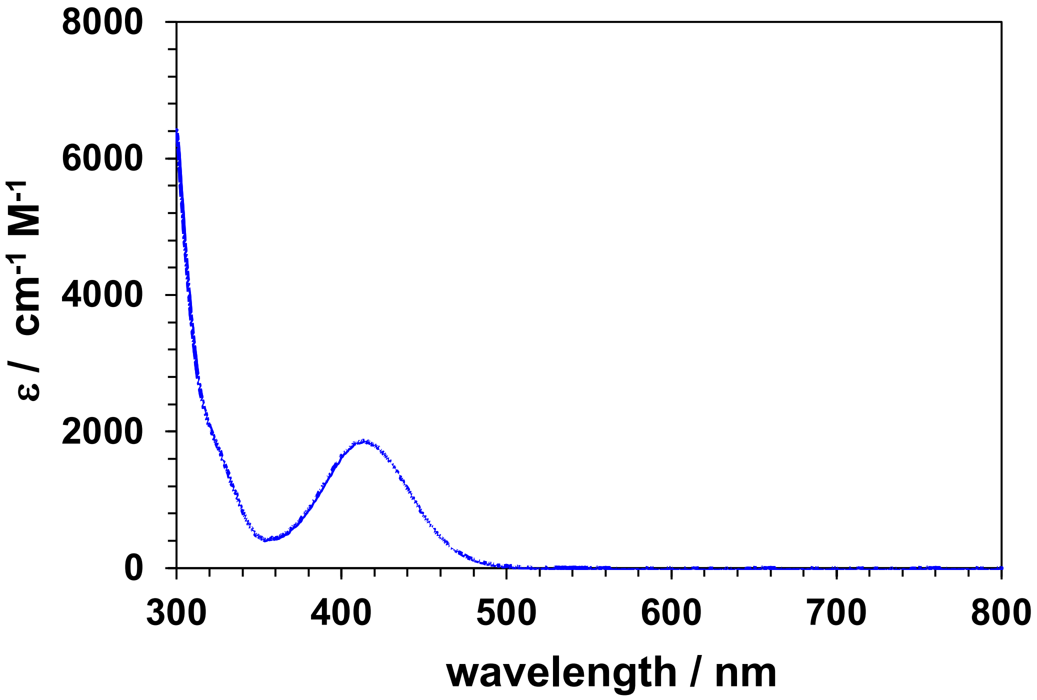

Figure S46. UV-Vis absorption spectrum [solid line, λ_{max} = 413 nm (ε = 1.9×10^3 cm^{-1} M^{-1})] of **2** in toluene solution.

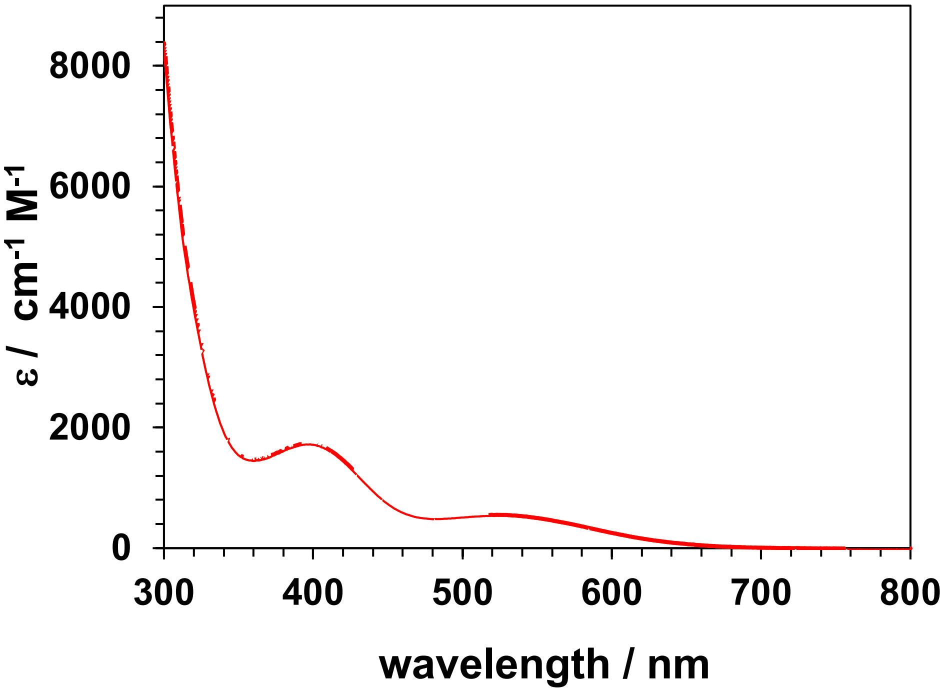

Figure S47. UV-Vis absorption spectrum [solid line, λ_{max} = 398 nm (ε = 1.7×10^3 cm^{-1} M^{-1}), λ_{max} = 523 nm (ε = 5.5×10^2 cm^{-1} M^{-1})] of **3a** in toluene solution.

Details for computational study

Optimization of ground state structures for real molecules 2, 3a, 3b, 4 and (IPr)Au–Bpin

Part of the computations were performed using workstation at Research Center for Computational Science, National Institutes of Natural Sciences, Okazaki, Japan. The theoretical approach is based on the framework of density functional theory (DFT). DFT calculations were performed by using Gaussian 16, Revision C.01 program package.[6] The geometry optimizations of **2**, **(IPr)Au–Bpin**, **3a**, **3b**, and **4** were performed at the PBE0[7]/6-311G(d)[8] (C, H, B, N, O), SDD[9] (Au) level of theory. All local minima were confirmed by their vibrational frequency calculations (with zero imaginary frequencies). QTAIM analysis was carried out using AIMALL software package[10] at the level of PBE0[7]/cc-pVDZ[11] (C,H,N,B), WTBS[12] (Au) level of theory.

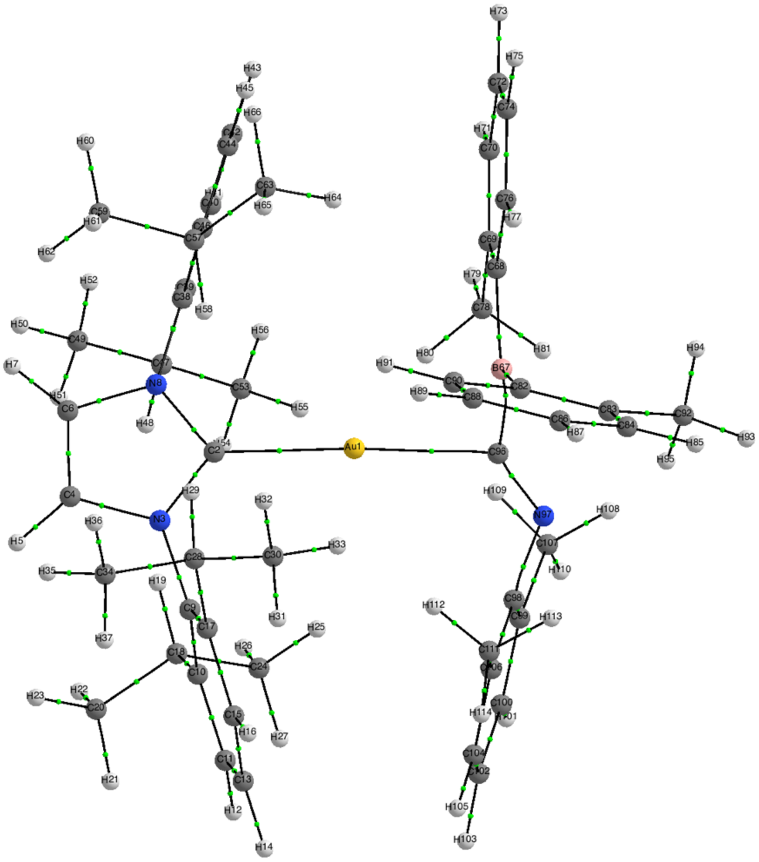

Figure S48. Molecular graphs of **4** with bond critical points (green spheres) and bond paths (solid lines: electron density at BCP $\geq$ 0.025 au) calculated by QTAIM analysis.

Searching the potential energy surface to find energy diagrams for Scheme 6-10 in the main text

All the calculations were performed with the Gaussian 09 program.[13] Geometry optimization was carried out using the PBE0 functional.[7, 14] In the DFT calculations, the 6-31G** basis set was used for all the other atoms while the triple-ζ SDD basis set[9a, 15] with the Stuttgart-Dresden ECP was employed for Au with polarization functions (ζ_f = 1.050)[16] being added. Frequency analysis was performed at the same level of theory to identify the nature of all the stationary points as minima (zero imaginary frequencies) or transition states (one imaginary frequency) and to provide free energies at 298.15 K which include entropic contributions by taking into account the vibrational,

rotational, and translational motions of the species under consideration. For each transition state, the intrinsic reaction coordinates (IRC)[17] analysis was conducted to ensure that it indeed connects two relevant local minima.

Table S2. Comparison between the PBE0 and PBE0-D3 energetic results (relative free energies in kcal/mol)

For the reaction of **2'** with PhCHO shown in Scheme 7

	PBE0	PBE0-D3
**2' + PhCHO**	0.0	0.0
**TS-coord_PhCHO**	15.3	7.3
**Coord_PhCHO**	11.4	3.1
**TS-add_PhCHO**	18.4	9.5
**Adduct_PhCHO**	-17.8	-27.0
**7'**	-27.5	-32.3

For the reaction of **2'** with Ph_2CO shown in Scheme 8

	PBE0	PBE0-D3
**2' + Ph_2CO**	0.0	0.0
**TS-coord_Ph_2CO**	15.1	5.7
**Coord_Ph_2CO**	11.2	1.2
**TS-add_Ph_2CO**	21.8	8.2
**8'**	-17.9	-29.3

For the reaction of **2'** with *p*-FC_6H_4COCl shown in Scheme 9

	PBE0	PBE0-D3
**2' + *p*-FC_6H_4COCl**	0.0	0.0
**TS-coord_ArCOCl**	18.6	10.2
**Coord_ArCOCl**	18.1	9.6
**TS-add_ArCOCl**	22.6	11.9
**Adduct_ArCOCl**	-19.0	-28.0
**TS-Cl-mig_ArCOCl**	-12.0	-20.7
**9'**	-34.2	-43.5

The results in the PBE0-D3 column are obtained from single-point calculations.

Remarks on Table S2:

One reviewer commented that the PBE0-calculated barriers seem relatively high for a reaction that is complete in under 1 minute. It is likely a result that PBE0 underestimates the dispersion attractive interactions between the diarylborylgold(I) complex and substrate molecules. To verify this reasoning, we carried out single-point energy calculations by including Grimme's D3 dispersion corrections[18] for those species shown in **Schemes 7-9**. The PBE0-D3 results, given in Table S1, indeed give noticeably smaller barriers, while qualitative conclusions made remain unchanged. However, the data obtained by the PBE0-D3 calculations seem to imply an over-correction by the method. Since the PBE0 results reproduce and explain well our experimental findings, plus inclusion of the corrections does

not alter the relative reactivity of the diarylborylgold(I) complex toward different unsaturated substrates, we did not further spend effort to find a better method to obtain more accurate absolute reaction barriers.

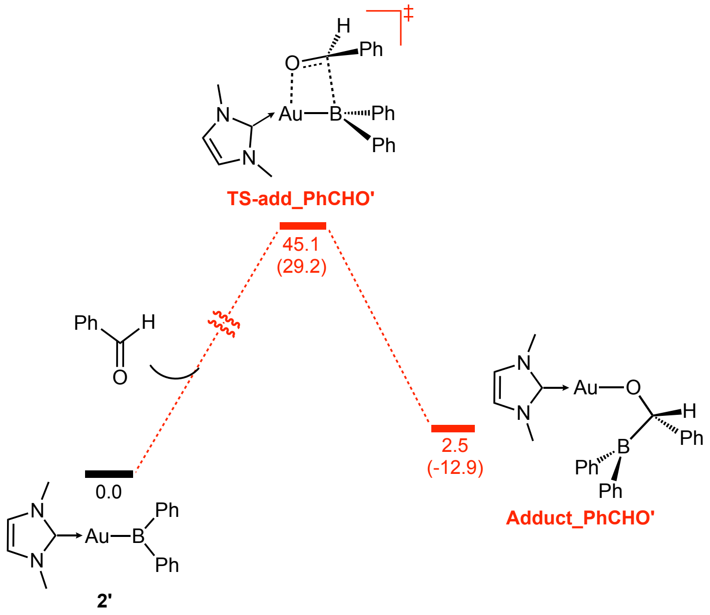

Figure S49. Energy profile calculated for the alternative pathway in which the O atom in the PhCHO coordinates to Au of model compound **2’**. The barrier is inaccessibly high, excluding such a possibility. Relative free energies and electronic energies (in parentheses) are given in kcal/mol.

References

1. N. Tsukahara, H. Asakawa, K.-H. Lee, Z. Lin and M. Yamashita, *J. Am. Chem. Soc.*, 2017, **139**, 2593-2596.
2. CrysAlisPRO, *CrysAlisPRO* Oxford Diffraction/Agilent Technologies UK Ltd, **2015**.
3. C. Kabuto, S. Akine and E. Kwon, *J. Cryst. Soc. Jpn.*, 2009, **51**, 218-224.
4. G. Sheldrick, *Act. Cryst. Sec. C*, 2015, **71**, 3-8.
5. M. C. Burla, R. Caliandro, B. Carrozzini, G. L. Cascarano, C. Cuocci, C. Giacovazzo, M. Mallamo, A. Mazzone and G. Polidori, *J. Appl. Crystallogr.*, 2015, **48**, 306-309.
6. M. J. Frisch, G. W. Trucks, H. B. Schlegel, G. E. Scuseria, M. A. Robb, J. R. Cheeseman, G. Scalmani, V. Barone, G. A. Petersson, H. Nakatsuji, X. Li, M. Caricato, A. V. Marenich, J. Bloino, B. G. Janesko, R. Gomperts, B. Mennucci, H. P. Hratchian, J. V. Ortiz, A. F. Izmaylov, J. L. Sonnenberg, Williams, F. Ding, F. Lipparini, F. Egidi, J. Goings, B. Peng, A. Petrone, T. Henderson, D. Ranasinghe, V. G. Zakrzewski, J. Gao, N. Rega, G. Zheng, W. Liang, M. Hada, M. Ehara, K. Toyota, R. Fukuda, J. Hasegawa, M. Ishida, T. Nakajima, Y. Honda, O. Kitao, H. Nakai, T. Vreven, K. Throssell, J. A. Montgomery Jr., J. E. Peralta, F. Ogliaro, M. J. Bearpark, J. J. Heyd, E. N. Brothers, K. N. Kudin, V. N. Staroverov, T. A. Keith, R. Kobayashi, J. Normand, K. Raghavachari, A. P. Rendell, J. C. Burant, S. S. Iyengar, J. Tomasi, M. Cossi, J. M. Millam, M. Klene, C. Adamo, R. Cammi, J. W. Ochterski, R. L. Martin, K. Morokuma, O. Farkas, J. B. Foresman and D. J. Fox, *Gaussian 16, Revision C.01* **2016**.
7. C. Adamo and V. Barone, *J. Chem. Phys.*, 1999, **110**, 6158-6170.
8. (a) S. Huzinaga, J. Andzelm, M. Klobukowski, E. Radzio-Andzelm, Y. Sakai and H. Tatewaki, *Gaussian basis sets*

for molecular calculations, Elsevier, 1984; (b) A. D. McLean and G. S. Chandler, *J. Chem. Phys.*, 1980, **72**, 5639-5648; (c) R. Krishnan, J. S. Binkley, R. Seeger and J. A. Pople, *J. Chem. Phys.*, 1980, **72**, 650-654.

9. (a) D. Andrae, U. Häußermann, M. Dolg, H. Stoll and H. Preuß, *Theor. Chim. Acta*, 1990, **77**, 123-141; (b) P. Schwerdtfeger, M. Dolg, W. H. E. Schwarz, G. A. Bowmaker and P. D. W. Boyd, *J. Chem. Phys.*, 1989, **91**, 1762-1774.
10. T. A. Keith, *AIMALL* (version 14.04.17), TK Gristmill Software, Overland Park KS, USA, **2014**.
11. D. E. Woon and T. H. D. Jr., *J. Chem. Phys.*, 1993, **98**, 1358-1371.
12. (a) S. Huzinaga and B. Miguel, *Chem. Phys. Lett.*, 1990, **175**, 289-291; (b) S. Huzinaga and M. Klobukowski, *Chem. Phys. Lett.*, 1993, **212**, 260-264.
13. M. J. Frisch, G. W. Trucks, H. B. Schlegel, G. E. Scuseria, M. A. Robb, J. R. Cheeseman, G. Scalmani, V. Barone, B. Mennucci, G. A. Petersson, H. Nakatsuji, M. Caricato, X. Li, H. P. Hratchian, A. F. Izmaylov, J. Bloino, G. Zheng, J. L. Sonnenberg, M. Hada, M. Ehara, K. Toyota, R. Fukuda, J. Hasegawa, M. Ishida, T. Nakajima, Y. Honda, O. Kitao, H. Nakai, T. Vreven, J. A. Montgomery Jr, J. E. Peralta, F. Ogliaro, M. J. Bearpark, J. Heyd, E. N. Brothers, K. N. Kudin, V. N. Staroverov, R. Kobayashi, J. Normand, K. Raghavachari, A. P. Rendell, J. C. Burant, S. S. Iyengar, J. Tomasi, M. Cossi, N. Rega, N. J. Millam, M. Klene, J. E. Knox, J. B. Cross, V. Bakken, C. Adamo, J. Jaramillo, R. Gomperts, R. E. Stratmann, O. Yazyev, A. J. Austin, R. Cammi, C. Pomelli, J. W. Ochterski, R. L. Martin, K. Morokuma, V. G. Zakrzewski, G. A. Voth, P. Salvador, J. J. Dannenberg, S. Dapprich, A. D. Daniels, Ö. Farkas, J. B. Foresman, J. V. Ortiz, J. Cioslowski and D. J. Fox, *Gaussian09, Revision D.01*, Gaussian, Inc., Wallingford, CT, 2009.
14. J. P. Perdew, K. Burke and M. Ernzerhof, *Phys. Rev. Lett.*, 1996, **77**, 3865-3868.
15. M. Dolg, U. Wedig, H. Stoll and H. Preuss, *J. Chem. Phys.*, 1987, **86**, 866-872.
16. A. W. Ehlers, M. Böhme, S. Dapprich, A. Gobbi, A. Höllwarth, V. Jonas, K. F. Köhler, R. Stegmann, A. Veldkamp and G. Frenking, *Chem. Phys. Lett.*, 1993, **208**, 111-114.
17. K. Fukui, *Acc. Chem. Res.*, 1981, **14**, 363-368.
18. S. Grimme, J. Antony, S. Ehrlich and H. Krieg, *J. Chem. Phys.*, 2010, **132**, 154104.
